# Investigating the causal effect of smoking on hay fever and asthma: a Mendelian randomization meta-analysis in the CARTA consortium

**DOI:** 10.1038/s41598-017-01977-w

**Published:** 2017-05-22

**Authors:** Tea Skaaby, Amy E. Taylor, Rikke K. Jacobsen, Lavinia Paternoster, Betina H. Thuesen, Tarunveer S. Ahluwalia, Sofus C. Larsen, Ang Zhou, Andrew Wong, Maiken E. Gabrielsen, Johan H. Bjørngaard, Claudia Flexeder, Satu Männistö, Rebecca Hardy, Diana Kuh, Sarah J. Barry, Line Tang Møllehave, Charlotte Cerqueira, Nele Friedrich, Tobias N. Bonten, Raymond Noordam, Dennis O. Mook-Kanamori, Christian Taube, Leon E. Jessen, Alex McConnachie, Naveed Sattar, Mark N. Upton, Charles McSharry, Klaus Bønnelykke, Hans Bisgaard, Holger Schulz, Konstantin Strauch, Thomas Meitinger, Annette Peters, Harald Grallert, Ellen A. Nohr, Mika Kivimaki, Meena Kumari, Uwe Völker, Matthias Nauck, Henry Völzke, Chris Power, Elina Hyppönen, Torben Hansen, Torben Jørgensen, Oluf Pedersen, Veikko Salomaa, Niels Grarup, Arnulf Langhammer, Pål R. Romundstad, Frank Skorpen, Jaakko Kaprio, Marcus R Munafò, Allan Linneberg

**Affiliations:** 1 Research Centre for Prevention and Health, Centre for Health, Capital Region of Denmark, Copenhagen, Denmark; 2 MRC Integrative Epidemiology Unit (IEU) at the University of Bristol, Bristol, UK; 3 UK Centre for Tobacco and Alcohol Studies, School of Experimental Psychology, University of Bristol, Bristol, UK; 4 Copenhagen Prospective Studies on Asthma in Childhood (COPSAC), Herlev and Gentofte Hospital, University of Copenhagen, Copenhagen, Denmark; 5 The Novo Nordisk Foundation Center for Basic Metabolic Research, Section on Metabolic Genetics, Faculty of Health and Medical Sciences, University of Copenhagen, Copenhagen, Denmark; 6 Steno Diabetes Center Copenhagen, Gentofte, 2820 Denmark; 7 Research unit for Dietary Studies, the Parker Institute, Frederiksberg and Bispebjerg Hospitals, The Capital Region, Frederiksberg Denmark; 8 Centre for Population Health Research, School of Health Sciences and Sansom Institute of Health Research, University of South Australia, Adelaide, Australia; 9 MRC Unit for Lifelong Health and Ageing at UCL, London, UK; 10 K.G. Jebsen Center for Genetic Epidemiology, Department of Public Health and Nursing, Faculty of Medicine and Health Sciences, Norwegian University of Science and Technology, NTNU, Trondheim, Norway; 11 Department of laboratory medicine, children’s and women’s health, Faculty of Medicine and Health Sciences, Norwegian University of Science and Technology, NTNU, Trondheim, Norway; 12 Forensic Department and Research Centre Bröset St. Olav’s University Hospital Trondheim, Trondheim, Norway; 13 Department of Public Health and Nursing, Faculty of Medicine and Health Sciences, Norwegian University of Science and Technology (NTNU), Trondheim, Norway; 14 Institute of Epidemiology I, Helmholtz Zentrum München – German Research Center for Environmental Health, Neuherberg, Germany; 15 Department of Health, National Institute for Health and Welfare, Helsinki, Finland; 16 Robertson Centre for Biostatistics, Institute of Health and Wellbeing, University of Glasgow, Glasgow, UK; 17 Institute of Clinical Chemistry and Laboratory Medicine, University Medicine Greifswald, Greifswald, Germany; 18 Department of Pulmonology, Leiden University Medical Center, Leiden, The Netherlands; 19 Department of Public Health and Primary Care, Leiden University Medical Center, Leiden, The Netherlands; 20 Department of Gerontology and Geriatrics, Leiden University Medical Center, Leiden, The Netherlands; 21 Department of Clinical Epidemiology, Leiden University Medical Center, Leiden, The Netherlands; 22 Department of BESC, Epidemiology Section, King Faisal Specialist Hospital and Research Centre, Riyadh, Saudi Arabia; 23 Department of Pulmonary Medicine, Ruhrlandklinik, West German Lung Center, University Hospital Essen, University Duisburg-Essen, Essen, Germany; 24 Institute of Cardiovascular and Medical Sciences & Institute of Health and Wellbeing, University of Glasgow, Glasgow, UK; 25 Helmsley Medical Centre, Helmsley, York UK; 26 Institute of Infection, Immunity and Inflammation, University of Glasgow, Glasgow, UK; 27 Comprehensive Pneumology Center Munich (CPC-M), Member of the German Center for Lung Research, Munich, Germany; 28 Institute of Genetic Epidemiology, Helmholtz Zentrum München – German Research Center for Environmental Health, Neuherberg, Germany; 29 Institute of Medical Informatics, Biometry and Epidemiology, Chair of Genetic Epidemiology, Ludwig-Maximilians-Universität, Munich, Germany; 30 Institute of Human Genetics, Helmholtz Zentrum München – German Research Center for Environmental Health, Neuherberg, Germany; 31 Institute of Human Genetics, Technische Universität München, Munich, Germany; 32 German Center for Cardiovascular Research (DZHK e.V.), Partner Site Munich Heart Alliance, München, Germany; 33 Research Unit Molecular Epidemiology, Helmholtz Zentrum München – German Research Center for Environmental Health, Neuherberg, Germany; 34 German Center for Diabetes Research, Neuherberg, Germany; 35 Institute of Epidemiology II, Helmholtz Zentrum München – German Research Center for Environmental Health, Neuherberg, Germany; 36 Research Unit for Gynaecology and Obstetrics, Institute of Clinical Research, University of Southern Denmark, Odense, Denmark; 37 Department of Epidemiology & Public Health, University College London, London, UK; 38 ISER, University of Essex, Colchester, UK; 39 Interfaculty Institute for Genetics and Functional Genomics, University Medicine and Ernst-Moritz-Arndt University Greifswald, Greifswald, Germany; 40 Institute for Community Medicine, University Medicine Greifswald, Greifswald, Germany; 41 Population, Policy and Practice, University College London Institute of Child Health, London, UK; 42 South Australian Health and Medical Research Institute, Adelaide, Australia; 43 Department of Public Health, Faculty of Health and Medical Sciences, University of Copenhagen, Copenhagen, Denmark; 44 Faculty of Medicine, Aalborg University, Aalborg, Denmark; 45 HUNT Research Centre, Department of Public Health and General Practice, Faculty of Medicine, Norwegian University of Science and Technology, Norwegian, Norway; 46 University of Helsinki, Dept. of Public Health, Helsinki, Finland; 47 National Institute for Health and Welfare, Dept. of Health, Helsinki, Finland; 48 University of Helsinki, Institute for Molecular Medicine, Helsinki, Finland; 49 Department of Clinical Experimental Research, Rigshospitalet, Glostrup Denmark; 50 Department of Clinical Medicine, Faculty of Health and Medical Sciences, University of Copenhagen, Copenhagen, Denmark

## Abstract

Observational studies on smoking and risk of hay fever and asthma have shown inconsistent results. However, observational studies may be biased by confounding and reverse causation. Mendelian randomization uses genetic variants as markers of exposures to examine causal effects. We examined the causal effect of smoking on hay fever and asthma by using the smoking-associated single nucleotide polymorphism (SNP) rs16969968/rs1051730. We included 231,020 participants from 22 population-based studies. Observational analyses showed that current vs never smokers had lower risk of hay fever (odds ratio (OR) = 0·68, 95% confidence interval (CI): 0·61, 0·76; P < 0·001) and allergic sensitization (OR = 0·74, 95% CI: 0·64, 0·86; P < 0·001), but similar asthma risk (OR = 1·00, 95% CI: 0·91, 1·09; P = 0·967). Mendelian randomization analyses in current smokers showed a slightly lower risk of hay fever (OR = 0·958, 95% CI: 0·920, 0·998; P = 0·041), a lower risk of allergic sensitization (OR = 0·92, 95% CI: 0·84, 1·02; P = 0·117), but higher risk of asthma (OR = 1·06, 95% CI: 1·01, 1·11; P = 0·020) per smoking-increasing allele. Our results suggest that smoking may be causally related to a higher risk of asthma and a slightly lower risk of hay fever. However, the adverse events associated with smoking limit its clinical significance.

## Introduction

Smoking is one of the most common modifiable risk factors for disease in adults. It has been suggested that smoking affects the risk of allergic respiratory disease and asthma^1–3^. Some studies have shown a positive association between smoking and asthma^4–6^, while others have found no or even an inverse association^7–9^, The effect of smoking on hay fever (allergic rhinitis) is also not clearly established although a systematic review and meta-analysis from 2014 of 34 observational studies (concerning active smoking and hay fever) found no association^1^. Allergic sensitization to inhalant allergens can be assessed by skin prick testing and/or measurements of serum specific IgE. These are generally accepted objective markers of allergic respiratory disease that can be used both in clinical assessment and epidemiological studies. Some but not all studies have observed a lower prevalence of allergic sensitization among current smokers compared to never smokers^1^, and we recently confirmed this in a meta-analysis of more than 20,000 participants from seven population-based studies^10^. However, inferring causal relationships between smoking and allergic respiratory disease from observational data is difficult due to confounding and reverse causation.

Mendelian randomization is a method for examining possible causal associations by using genetic variants with well-known effects on exposure patterns as proxies for exposure^11^. Mendelian randomization is based on the assumption of random allocation of alleles from parent to child. It takes advantage of the fact that the genetic variants will not be associated with the confounding factors and reverse causality inherent in conventional observational studies.

The rs16969968 single nucleotide polymorphism (SNP) is associated with smoking heaviness within smokers. The risk allele, here the minor allele, is associated with an average increase in smoking amount of one cigarette per day in smokers^12–14^. The rs16969968 SNP is in perfect linkage disequilibrium with rs1051730, and they are used interchangeably. These genetic proxies for smoking, unlike smoking heaviness itself, are not associated with confounding factors that may distort associations with health outcomes, for example, socioeconomic status and education level^15^. To test the causal nature of the associations between smoking and hay fever, asthma, and allergic sensitization, we performed a Mendelian randomization meta-analysis combining data from 22 studies in the Causal Analysis Research in Tobacco and Alcohol (CARTA) consortium and the UK Biobank.

## Methods

### Study populations

The study was performed as a meta-analysis within the CARTA consortium (http://www.bris.ac.uk/expsych/research/brain/targ/research/collaborations/carta). We used data on 231,020 participants of self-reported European ancestry and aged ≥16 years from 22 studies from the CARTA consortium: The British 1958 Birth Cohort (1958BC), the Avon Longitudinal Study of Parents and Children (ALSPAC) Mothers, ALSPAC Children, COPSAC2000, the Danish Monitoring of trends and determinants in Cardiovascular Diseases (MONICA) study (the Dan-Monica10 study), the English Longitudinal Study of Ageing (ELSA), the National FINRISK Study (FINRISK), Genomics of Overweight in Young Adults (GOYA) Females, GOYA Males, Health2006, Health2008, the second wave of the Nord-Trøndelag health study (HUNT2), Inter99, the Cooperative Health Research in the Region of Augsburg (KORA) study, the Middle-aged Span-of-Life (MIDSPAN) Family Study, the MRC National Survey of Health and Development (NSHD), the 1936 Cohort, the UK Biobank, the Netherlands Epidemiology of Obesity (NEO) study, Whitehall II, the Study of Health in Pomerania (SHIP) and SHIP-TREND (See online supplementary material).

The British 1958 Birth Cohort was approved by the South-East Multi-Centre Research Ethics Committee and the joint UCL/UCLH Committees on the Ethics of Human Research. The ALSPAC Mothers and Children were approved by the ALSPAC Ethics and Law Committee and the Research Ethics Committee. COPSAC2000 was approved by Copenhagen Ethics Committee and the Danish Data Protection Agency. The Dan-Monica10 study, the Health2006 Study, the Health2008 Study, the Inter99 Study, and the 1936 Cohort were approved by the Ethics Committee of Copenhagen County and the Danish Data Protection Agency. ELSA was approved by the National Research Ethics Service. FINRISK was approved by the Coordinating Ethics Committee for the Uusimaa Hospital District. GOYA Females was approved by the Ethical Committee of Copenhagen and the Danish Data Protection Board. GOYA Males was approved by the Ethics Committee for Copenhagen and the Danish Data Protection Board. HUNT2 was approved by the Regional Committee for Medical Research Ethics. KORA was approved by the Ethics Committee of the Bavarian Medical Association. MIDSPAN Family Study was approved by the Argyll and Clyde Health Board Local Research Ethics Committee. NSHD was approved by the Central Manchester Research Ethics Committee. UK Biobank was approved by the Ethics and Governance Council. The NEO study was approved by the Medical Ethical Committee of the Leiden University Medical Center. Whitehall II was approved by the University College London Medical School committee on the ethics of human research. SHIP and SHIP-TREND were approved by the Ethics Committee of the University of Greifswald. All participants gave their informed consent, and all methods were carried out in accordance with relevant guidelines and regulations (more information in the Supplementary).

### Genotype

Each participant was genotyped for either rs16969968 or rs1051730. Both are located in the *CHRNA5-A3-B4* nicotinic receptor subunit gene cluster and in perfect linkage disequilibrium in Europeans (R^2^ = 1·00 in HapMap 3, http://hapmap.ncbi.nlm.nih.gov/). Description of the method for genotyping within each study is provided in the online supplementary material.

### Measures of hay fever, asthma, and allergic sensitization

Data on hay fever and asthma were based on self-report. Our first choice was lifetime/ever diagnoses, but alternatively we used a diagnosis in the past 12 months or longer. Allergic sensitization was defined as serum specific IgE positivity to at least one of the tested inhalant allergens. The study-specific measures of hay fever, asthma and allergic sensitization are provided in Table S3.

### Smoking status

Smoking status classified as never, former, current or ever (former and current smokers) cigarette smokers was assessed at the same time as the outcome if available. Smoking heaviness was measured as cigarettes smoked per day or recoded to the midpoint of the category. More information is provided in the online supplemental material and the protocol: http://www.bris.ac.uk/expsych/research/brain/targ/research/collaborations/carta/.

### Statistical analyses

Analyses were conducted within each contributing study according to the same pre-specified analysis protocol: http://www.bris.ac.uk/expsych/research/brain/targ/research/collaborations/carta/.

We restricted the analyses to participants with data on disease outcomes (at least one of the three outcomes), smoking status and rs16969968/rs1051730 genotype. Sex- and age-adjusted associations of smoking status (never [reference group], former, current, ever) and smoking heaviness with dichotomous measures of hay fever, asthma, and allergic sensitization were assessed using logistic regression. The smoking heaviness analyses were restricted to current smokers and to studies with continuous measures of cigarettes per day. Hence, odds ratios (ORs) represent differences in odds of the outcome measure per additional cigarette consumed per day.

The genotype frequencies were tested for deviation from Hardy-Weinberg equilibrium (HWE) using a χ^2^ exact test within each study. Mendelian randomization analyses of the association between rs16969968/rs1051730 and dichotomous measures of hay fever, asthma and allergic sensitization were performed using logistic regression, both unadjusted and adjusted for age and sex. We stratified the analyses by smoking status (never, former, current and ever), because the variant only influences smoking heaviness in smokers^16^. We assumed an additive genetic model which means that ORs represent the ratio in odds of the outcome per additional copy of the smoking-increasing allele.

The results were meta-analyzed in Stata, version 12.1 (StataCorp LP, College Station, Texas, USA) using the ‘metan’ command where heterogeneity was evaluated by the I-square test^17, 18^. If there was evidence of heterogeneity between studies (I^2^ > 50%), we performed both fixed and random effect analyses (Figs 1–3 and Supplementary Figures S1–S24). The random effects model was based on the method of DerSimonian & Laird and the estimate of heterogeneity from the Mantel-Haenszel model^18^.

**Figure 1:**
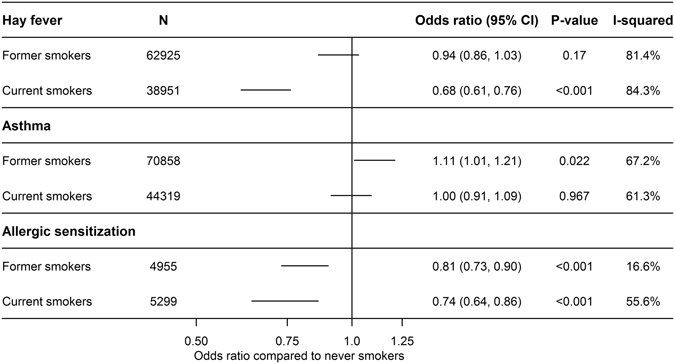
Age- and sex-adjusted associations of smoking status with hay fever, asthma and allergic sensitization using random effect meta-analysis.

**Figure 2:**
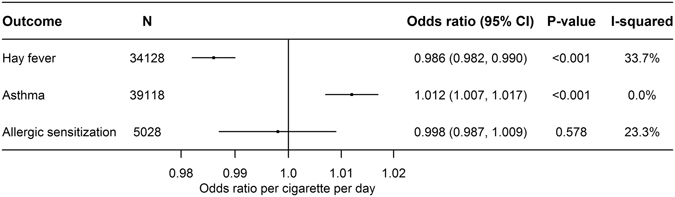
Age- and sex-adjusted associations of smoking heaviness with hay fever, asthma and allergic sensitization using fixed effect meta-analysis.

**Figure 3:**
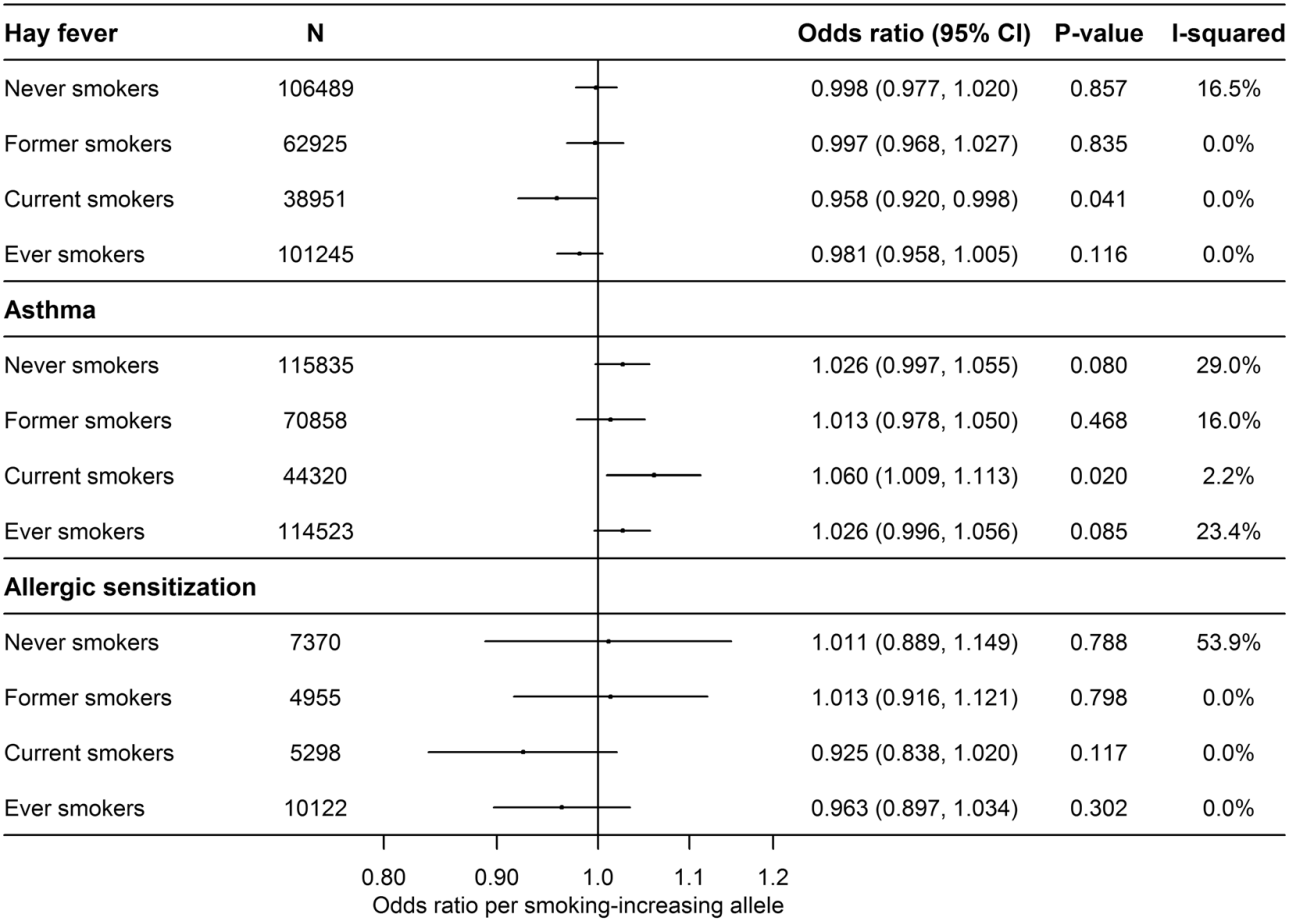
Mendelian randomization analysis of the age- and sex-adjusted associations of rs1051730 or rs16969968 and hay fever (N = 208,365), asthma (N = 231,013) and allergic sensitization (N = 17,623) using fixed effect meta-analysis, except for allergic sensitization where we used random effect meta-analysis. Please note that the sum of former and current smokers is not equal to the number of ever smokers since GOYA Females are included in current smokers but not in ever smokers.

The results from meta-analyses are summarized in Figs 1–3. The detailed meta-analyses showing age- and sex-adjusted study-specific estimates are shown in Supplementary Figures S1–S8 (the crude associations are shown in S9–S16). We also performed analyses with and without UK Biobank (Figures S17–S20), and without ALSPAC Mothers and ALSPAC Children (Figures S21–S22 and Figures S23–S24, respectively).

### Role of the funding source

The study sponsors were not involved in study design; in the collection, analysis, and interpretation of data; in the writing of the report; and in the decision to submit the paper for publication. The corresponding author had full access to all the data in the study and had final responsibility for the decision to submit for publication.

## Results

### Descriptive statistics

In total, we had data on 231,020 participants, including 115,839 never smokers, 70,858 former smokers and 44,323 current smokers. Overall, 45·5% of the combined study population were males (N_males_ = 105,203). The median age within the contributing studies ranged from 18 to 64 years. Descriptive statistics for each of the study populations are found in the Supplemental Table S1. Minor allele frequency for rs16969968/rs1051730 ranged between 0·32 and 0·37 (Supplemental Table S2). The genotype distribution did not deviate from Hardy Weinberg Equilibrium in any of the studies (P-values all ≥ 0·05) (Supplemental Table S2). For the Mendelian randomization analyses, the number of participants were for hay fever: N = 208,365, asthma: N = 231,013, and allergic sensitization: N = 17,623. The percentage with hay fever (N_hayfever_ = 41,170), asthma (N_asthma_ = 24,199) and allergic sensitization (N_allergic sensitization_ = 4,573) varied between 8·0–54·0%, 3·7–61·7%, and 13·8–50·3%, respectively (Table S1).

### Observational analyses

Figures 1 and 2 show the estimates from meta-analyses of the age- and sex-adjusted associations of smoking status and smoking heaviness with hay fever, asthma and allergic sensitization, respectively. For current smokers (but not former smokers) compared to never smokers, we found a lower risk of hay fever (OR = 0·68, 95% CI: 0·61, 0·76; P < 0·001) and, accordingly, we found an inverse dose-response relationship between smoking heaviness and hay fever (OR = 0·99 per cigarette per day, 95% CI: 0·98, 0·99; P < 0·001) (Fig. 2). We found a higher risk of asthma in former smokers compared with never smokers (OR = 1·11, 95% CI: 1·01, 1·21; P = 0·022), but not for current smokers (OR = 1·00, 95% CI: 0·91, 1·09; P = 0·967) (Fig. 1). However, there was a positive dose-response relationship between smoking heaviness and asthma in current smokers (OR = 1·01, 95% CI: 1·01, 1·02; P < 0·001) (Fig. 2). For allergic sensitization, we found a lower risk in former (OR = 0·81, 95% CI: 0·73, 0·90; P < 0·001) and current smokers (OR = 0·74, 95% CI: 0·64, 0·86; P < 0·001) compared with never smokers (Fig. 1), but no association with smoking heaviness (OR = 1·00, 95% CI: 0·99, 1·01; P = 0·578) (Fig. 2).

The heterogeneity in the analyses of smoking status was considerable (>80%, Fig. 1), and the estimates presented are therefore based on random effect meta-analysis. The smoking heaviness analyses showed little evidence of heterogeneity and were analysed with fixed effect meta-analysis (Fig. 2).

### Mendelian randomization analyses

Our results confirmed that the smoking-increasing allele of rs1051730/rs16969968 is associated with an increase in smoking heaviness of approximately 1 cigarette/day in current smokers (Figure S8). Figure 3 shows the Mendelian randomization analyses of the age- and sex-adjusted associations of rs1051730/rs16969968 and hay fever, asthma, and allergic sensitization. We found some evidence that the smoking-increasing allele was associated with lower odds of hay fever in current smokers (OR = 0·958, 95% CI: 0·920, 0·998; P = 0·041). There were no associations of genotype with disease in never (OR = 1·00, 95% CI: 0·98, 1·02; P = 0·857) or former smokers (OR = 1·00, 95% CI: 0·97, 1·03; P = 0·835) (Fig. 3). In line with this, the smoking-increasing allele tended to associate with lower risk of allergic sensitization in current smokers (OR = 0·92, 95% CI: 0·84, 1·02; P = 0·117). In contrast, the smoking-increasing allele was associated with a higher risk of asthma in current smokers (OR = 1·06, 95% CI: 1·01, 1·11; P = 0·020) (Fig. 3). The same tendency was found in the never smokers (OR = 1·03, 95% CI: 1·00, 1·06; P = 0·080).

The analyses of smoking heaviness according to genotype showed little heterogeneity between studies and were analysed with fixed effect meta-analysis (Figure S8). Except for allergic sensitization showing moderate heterogeneity, the heterogeneity in the analyses of rs1051730/rs16969968 in relation to disease outcomes was low (Fig. 3). Therefore, the analyses for hay fever/asthma and allergic sensitization were performed with fixed and random effect meta-analysis, respectively. In general, the unadjusted Mendelian randomization analyses yielded similar results which were expected since the genetic variant should be independent of confounding factors (Figures S13–S15).

### Supplementary analyses

Since UK Biobank data represented the largest sample and because approximately one third of the UK Biobank sample with genetic data was selected on smoking status, and lung function (including criteria related to asthma), analyses without UK Biobank data (Figures S17–S18) and of UK Biobank samples alone (Figures S19–S20) were performed^19^. ALSPAC Mothers and ALSPAC Children were analyzed as separate samples in the main analyses although the mothers and children were related. However, the results from analyses excluding ALSPAC Mothers and ALSPAC Children, respectively, showed similar results (Figures S21–S22 and figures S23–S24). In analyses of UK Biobank data only, we adjusted the observational analysis of the association of smoking status with hay fever and asthma in addition for total household income. This additional adjustment for household income changed the odds ratios for current and former smokers compared to never smokers less than 5% (0.9–4.9%). In addition, the smoking associated SNP was not associated with total household income in the UK Biobank Study (Chi^2^-test: p = 0.99).

## Discussion

A meta-analysis of 22 population-based studies was performed including 231,020 participants of European ancestry using both observational and Mendelian randomization analyses. In the latter, we found a slightly lower risk of hay fever and higher risk of asthma in current smokers with genetically determined heavier smoking. However, these associations were not evident among ever smokers. We also observed a tendency toward a lower risk of allergic sensitization associated with smoking. However, the power to show an effect was much lower for allergic sensitization, and there may still be a small to moderate effect of smoking on the risk of allergic sensitization. In general, our MR findings supported our observational analyses.

The observed inverse associations of smoking and hay fever among current smokers in both observational and Mendelian randomization analyses are somewhat in contrast with a meta-analysis by Saulyte *et al*. who found no association between smoking and allergic rhinitis^1^. The inverse association between smoking and allergic respiratory disease may reflect an immunosuppressive effect of smoking but could also (at least in the observational analysis) be due to reverse causality^20, 21^. It is possible that allergic smokers are more likely not to smoke or to quit smoking, sometimes referred to as the healthy smoker effect, i.e. the observed inverse association of smoking and hay fever may not be causal. Our results from the Mendelian randomization approach seem to support the results of the traditional observational approach in that smoking increases risk of asthma but decreases risk of hay fever. As regards observational analyses, they show that ex-smokers have lower risk of atopy and it is also important to note that longitudinal analyses have shown lower incidence of atopy in current smokers^20^, findings that are difficult to reconcile with the idea that smokers who develop an allergy tend to quit smoking.

The observed positive association between smoking and asthma among current smokers in our Mendelian randomization analysis and observational analysis of smoking heaviness is in line with a study by Coogan *et al*. who found that active smoking increased the incidence of adult-onset asthma and evidence for a dose-response relationship in a study of more than 45,000 African-American women with a 16 year follow-up^6^. Other studies have found similar associations^5^. However, some studies have found a lack of or an inverse association^7–9^. Possible mechanisms include a smoking-induced increased Th2 response and airway hyper-reactivity^22^. Studies in humans and animals have reported several effects of tobacco smoke on the airways (e.g., increased permeability, inflammation, and changes in gene expression)^23, 24^. The inhaled smoke comes into direct contact with the airway epithelium. Beyond being a barrier, the epithelium is important in immune regulation: it influences inflammatory cell recruitment through cytokine and chemokine secretion and influences remodeling of the tissue through growth factors^25^. In addition, increased exposure to passive smoking among children carrying and “sharing” the smoking-increasing allele with their parents may affect early immune programming and the risk of asthma in the offspring^26, 27^. This may also potentially explain the borderline (positive) association in never smokers. In addition, the borderline effect in never smokers may also be due to, for example, former smokers being falsely classified as never smokers, and differential underreporting of smoking habits. However, the three studies that have potentially included both never and ex-smokers in the never smokers category (COPSAC2000, GOYA Females, and NSHD) do not seem to explain the borderline association among never smokers. Smokers in general tend to underreport their smoking habits, and smokers with asthma may be even more likely to underreport their smoking compared to smokers without asthma and smokers with diseases that are not believed to be related to smoking. This will tend to falsely increase the effect in never smokers but only if the current smokers with asthma underreporting smoking are unequally distributed regarding the SNP; otherwise it will not make a difference. However, it will tend to underestimate the effect in current smokers.

The observed tendency toward a lower risk of allergic sensitization associated with smoking is somewhat comparable to the observational association as well as to several previous studies^20, 28, 29^. Recently, we found a lower prevalence of allergic sensitization among current smokers versus never smokers in 20,048 participants from seven Danish studies^10^ that may reflect an immunosuppressive effect of smoking^20^. Compared with hay fever and asthma, we had substantially less data on allergic sensitization and thus may lack power to show a moderate or weak effect of smoking on allergic sensitization, so caution must be taken in ruling out an effect on allergic sensitization.

The reasons why the observed effects of the smoking-increasing allele were mainly seen in current smokers and not in former smokers are not clear. It is plausible that an effect of any given exposure decreases with increasing time following ceased exposure. We did not have data to investigate that hypothesis. Assuming that the effects of smoking on hay fever and asthma go through immunological pathways it may also be hypothesized that the effects decrease relatively fast following smoking cessation, which could explain why the smoking increasing allele does not have any effects among former smokers.

The major strengths of this study are the large sample size and the inclusion of different populations. We used objective markers of allergic sensitization (i.e., serum specific IgE positivity against inhalant allergens) that may be more reliable than self-reported diagnoses and symptoms. We performed a number of supplementary analyses with the results largely unchanged. Using a genetic marker of exposure should support stronger causal inference because genetic variants should not be associated with the usual confounding factors, they will indicate long-term levels of exposure, and are not affected by the onset of disease and thus protected from reverse causation. The smoking-associated rs16969968/rs1051730 genotype is strongly and consistently associated with smoking heaviness among smokers, has shown to be a solid instrument for smoking, and has shown the expected causal associations with increased all-cause mortality, decreased lung function, and BMI^30–35^. However, using more than a single SNP, e.g., SNPs reflecting different pathways to the exposure, may reduce the risk of pleiotropy, but we know of no other smoking-associated SNP with strength and consistency similar to the rs16969968/rs1051730 genotype, so this might introduce weak instrument bias^36^.

A limitation of the current study is the use of self-reported hay fever and asthma rather than clinical doctor-verified diagnoses or objective markers and that different questionnaires were used across studies. Further, we did not have information about the disease severity. The use of “ever” phenotypes is potentially problematic, since the outcome could precede smoking behavior, and it does not allow us to see whether smoking worsens symptoms of allergy/asthma. The prevalence of outcomes varied between populations possibly due to differences between populations in age, socioeconomic factors, and year of examination. This could influence our results and induce heterogeneity, which we also observed in the observational analyses. However, the heterogeneity of the MR analyses was relatively low suggesting that the variation in outcome prevalence did not introduce substantial heterogeneity in those analyses. Lacking a longitudinal design, we were unable to distinguish between the incidence, persistence or recurrence of asthma. Regarding asthma in particular, it is difficult to distinguish between chronic obstructive pulmonary disease (COPD) and asthma using self-report, and it is possible that some of those who reported to have asthma may have had COPD. In addition, some may suffer from the overlap syndrome of asthma and COPD^37^. Misclassification of participants with COPD as having asthma would tend to inflate the observed association between smoking and asthma. The studies including only persons younger than 50 years may represent a more precise asthma group. However, all studies with participants younger than 50 years (1958 BC, ALSPAC Children, and GOYA Females) and the studies where participants are below 52 years (ALSPAC Mothers, and COPSAC2000) have odds ratios larger than one. Given the size of the current study, it may be reasonable to conclude that smoking is associated with a higher risk of asthma, in spite of some potential misclassification. Collider bias of Mendelian randomization analyses may arise from stratification if the instrument is predictive of the stratification parameter. However, since our instrument rs16969968/rs1051730 is associated with smoking heaviness in smokers rather than with smoking initiation, we consider this to be a minor risk. The reason for not performing a formal instrumental variable analysis is the imprecision in self-reported cigarettes/day as a measure of exposure that may lead to severely biased estimates^38^. However, this does not affect the causal insights of the Mendelian randomization approach.

The effects of smoking on the immune system in general are not clear but accumulating evidence suggests that smoking compromises the immune response^39^. Of the more than 45,000 chemicals contained in cigarette smoke, tar and nicotine are believed to be the most important regarding smoking’s effect on the immune system. Studies suggest that they suppress the immune response and increase the susceptibility to infections in various ways. Nicotine has been found to negatively affect antigen mediated signal transduction in lymphocytes and induces a state of T cell anergy^39^. Chronic exposure to tobacco smoke has been found to decrease levels of surfactant in the lungs, impair the ciliary epithelium, and reduce phagocytic function of macrophages to clear inflammation and debris from the lungs. In animal studies, chronic exposure to cigarette smoke or nicotine inhibits the responsiveness of T cells with decreased antibody response. Smoking has also been found to alter the function of neutrophils and the immune response of the lymphocytes^39^.

Mendelian randomization is a powerful tool for strengthening causal inference in epidemiological studies. It is becoming increasingly popular as a supplement to observational studies and an alternative to randomized controlled trials (RCTs), and it is clarifying a number of previously misconceived associations^40, 41^. Compared to RCTs, Mendelian randomization studies require no random treatment allocation, are more feasible, and often have fewer ethical concerns. MR studies can like other observational studies be performed in a representative sample in contrary to RCTs that are frequently carried out in otherwise healthy adults. However, potential violators of the inherent Mendelian randomization assumptions include canalization (i.e., developmental changes trying to compensate for the genetic variation), linkage disequilibrium between the SNP and other causal variants, and biological pleiotropy where the genetic variant has diverse biological functions.

This large Mendelian randomization meta-analysis suggests that smoking may be causally related to a higher risk of asthma, and asthma should maybe be added to the long list of smoking-induced diseases. Thus, our results strengthen advice against smoking to reduce incidence and burden of chronic diseases. On the other hand, our results are somewhat supportive of a minor preventive effect of smoking on hay fever. However, this hypothesis needs confirmation and further investigation of the possible pathogenic pathways. The high frequency of adverse events associated with smoking, to a great extent limits its clinical significance.

## Electronic supplementary material

Supplementary Information
