# Supplementary material for: Investigating the causal effect of smoking on hay fever and asthma: a Mendelian randomization meta-analysis in the CARTA consortium
Source: Sci Rep. 2017 May 22;7:2224. doi: 10.1038/s41598-017-01977-w (PMC5440386; doi:10.1038/s41598-017-01977-w)
Supplement: Supplementary file 1 — Supplementary Information [file 41598_2017_1977_MOESM1_ESM.pdf]

Supplementary material

Investigating the causal effect of smoking on hay fever and asthma: a Mendelian randomization meta-analysis in the CARTA consortium

Authors

Tea Skaaby, PhD[1], Amy E. Taylor, PhD[2,3], Rikke K. Jacobsen, MSc[1], Lavinia Paternoster, PhD[2], Betina H. Thuesen, PhD[1], Tarunveer S. Ahluwalia, PhD[4,5,6], Sofus C. Larsen, PhD[7], Ang Zhou, PhD[8], Andrew Wong, PhD[9], Maiken E. Gabrielsen, PhD[10,11], Johan H. Bjørngaard, PhD[12, 13], Claudia Flexeder, MSc[14], Satu Männistö, PhD[15], Rebecca Hardy, PhD[9], Diana Kuh, PhD[9], Sarah J. Barry, PhD[16], Line Tang Møllehave, MSc[1], Charlotte Cerqueira, PhD[1], Nele Friedrich, PhD[1,17], Tobias N. Bonten, PhD[18,19] Raymond Noordam, PhD[20],

1 Research Centre for Prevention and Health, Centre for Health, Capital Region of Denmark, Copenhagen, Denmark.
2 MRC Integrative Epidemiology Unit (IEU) at the University of Bristol, Bristol, UK
3 UK Centre for Tobacco and Alcohol Studies, School of Experimental Psychology, University of Bristol, Bristol, UK
4 Copenhagen Prospective Studies on Asthma in Childhood (COPSAC), Herlev and Gentofte Hospital, University of Copenhagen, Copenhagen, Denmark
5 The Novo Nordisk Foundation Center for Basic Metabolic Research, Section on Metabolic Genetics, Faculty of Health and Medical Sciences, University of Copenhagen, Copenhagen, Denmark
6 Steno Diabetes Center Copenhagen, Gentofte 2820, Denmark
7 Research unit for Dietary Studies, the Parker Institute, Frederiksberg and Bispebjerg Hospitals, The Capital Region, Frederiksberg, Denmark
8 Centre for Population Health Research, School of Health Sciences and Sansom Institute of Health Research, University of South Australia, Adelaide, Australia
9 MRC Unit for Lifelong Health and Ageing at UCL, London, UK
10 K.G. Jebsen Center for Genetic Epidemiology, Department of Public Health, Faculty of Medicine, NTNU, Norwegian University of Science and Technology, Trondheim, Norway
11 Department of laboratory medicine, children’s and women’s health, Faculty of Medicine, NTNU Norwegian University of Science and Technology, Trondheim, Norway
12 Forensic Department and Research Centre Bröset St. Olav’s University Hospital Trondheim, Trondheim, Norway
13 Department of Public Health, Norwegian University of Science and Technology (NTNU), Norway
14 Institute of Epidemiology I, Helmholtz Zentrum München – German Research Center for Environmental Health, Neuherberg, Germany
15 Department of Health, National Institute for Health and Welfare, Helsinki, Finland
16 Robertson Centre for Biostatistics, Institute of Health and Wellbeing, University of Glasgow
17 Institute of Clinical Chemistry and Laboratory Medicine, University Medicine Greifswald, Germany
18 Department of Pulmonology, Leiden University Medical Center, Leiden, the Netherlands

Dennis O. Mook-Kanamori, PhD[19,21,22], Christian Taube, MD[23], Leon E. Jessen, PhD[4], Alex McConnachie, PhD[16], Naveed Sattar, PhD[24], Mark N. Upton, MD[25], Charles McSharry, PhD[26], Klaus Bønnelykke, PhD[4], Hans Bisgaard, DMSc[4], Holger Schulz, MD[14, 27], Konstantin Strauch, PhD[28,29], Thomas Meitinger, MD[30,31,32], Annette Peters, PhD[32,33] , Harald Grallert, PhD[32 ,34, 35], Ellen A. Nohr, PhD[36], Mika Kivimaki, PhD[37], Meena Kumari, PhD[38], Uwe Völker, PhD[39], Matthias Nauck, MD[17], Henry Völzke, PhD[40], Chris Power, PhD[41], Elina Hyppönen, PhD[8,41,42], Torben Hansen, PhD[5], Torben Jørgensen, Dr. Med.[1,43,44], Oluf

---

19 Department of Public Health and Primary Care, Leiden University Medical Center, Leiden, the Netherlands
20 Department of Gerontology and Geriatrics, Leiden University Medical Center, Leiden, the Netherlands
21 Department of Clinical Epidemiology, Leiden University Medical Center, Leiden, the Netherlands
22 Department of BESC, Epidemiology Section, King Faisal Specialist Hospital and Research Centre, Riyadh, Saudi Arabia
23 Department of Pulmonary Medicine, Ruhrlandklinik, West German Lung Center, University Hospital Essen, University Duisberg-Essen, Essen, Germany
24 Institute of Cardiovascular and Medical Sciences & Institute of Health and Wellbeing, University of Glasgow, UK
25 Helmsley Medical Centre, Helmsley, York, UK
26 Institute of Infection, Immunity and Inflammation, University of Glasgow, Glasgow, UK
27 Comprehensive Pneumology Center Munich (CPC-M), Member of the German Center for Lung Research, Munich, Germany
28 Institute of Genetic Epidemiology, Helmholtz Zentrum München – German Research Center for Environmental Health, Neuherberg, Germany
29 Institute of Medical Informatics, Biometry and Epidemiology, Chair of Genetic Epidemiology, Ludwig-Maximilians-Universität, Munich, Germany
30 Institute of Human Genetics, Helmholtz Zentrum München – German Research Center for Environmental Health, Neuherberg, Germany
31 Institute of Human Genetics, Technische Universität München, Munich, Germany
32 German Center for Cardiovascular Research (DZHK e.V.), Partner Site Munich Heart Alliance, München, Germany
33 Research Unit Molecular Epidemiology, Helmholtz Zentrum München – German Research Center for Environmental Health, Neuherberg, Germany
34 German Center for Diabetes Research, Neuherberg, Germany
35 Institute of Epidemiology II, Helmholtz Zentrum München – German Research Center for Environmental Health, Neuherberg, Germany
36 Research Unit for Gynaecology and Obstetrics, Institute of Clinical Research, University of Southern Denmark, Odense, Denmark
37 Department of Epidemiology & Public Health, University College London, London, UK
38 ISER, University of Essex, Colchester, UK
39 Interfaculty Institute for Genetics and Functional Genomics, University Medicine and Ernst-Moritz-Arndt University Greifswald, Germany
40 Institute for Community Medicine, University Medicine Greifswald, Germany
41 Population, Policy and Practice, University College London Institute of Child Health, London, UK
42 South Australian Health and Medical Research Institute, Adelaide, Australia
43 Department of Public Health, Faculty of Health and Medical Sciences, University of Copenhagen, Denmark
44 Faculty of Medicine, Aalborg University, Denmark

Pedersen, DMSc[5], Veikko Salomaa, PhD[47], Niels Grarup, PhD[5], Arnulf Langhammer, PhD[45], Pål R. Romundstad, PhD[13], Frank Skorpen, PhD[11], Jaakko Kaprio, PhD[46,47,48], Marcus R. Munafò, PhD[2,3], Allan Linneberg, PhD[1,49,50]

45 HUNT Research Centre, Department of Public Health and General Practice, Faculty of Medicine, Norwegian University of Science and Technology, Norway
46 University of Helsinki, Dept. of Public Health, Helsinki, Finland
47 National Institute for Health and Welfare, Dept. of Health, Helsinki, Finland
48 University of Helsinki, Institute for Molecular Medicine, Helsinki, Finland
49 Department of Clinical Experimental Research, Rigshospitalet, Glostrup, Denmark
50 Department of Clinical Medicine, Faculty of Health and Medical Sciences, University of Copenhagen, Copenhagen, Denmark

CONTENTS

STUDY DESCRIPTIONS 6
British 1958 Birth Cohort 6
ALSPAC 7
ALSPAC Mothers 8
ALSPAC Children 8
COPSAC2000 9
The Dan-Monica10 study 10
ELSA 11
FINRISK 13
GOYA Females 14
GOYA Males 15
Health2006 16
Health2008 17
HUNT2 18
Inter99 19
KORA 20
MIDSPAN Family Study 21
The NEO study 22
NSHD 23
The 1936 Cohort 24
UK Biobank 25
Whitehall II 27
SHIP 27
SHIP TREND 28

MAIN SUPPLEMENTARY TABLES 30
Table S1 30
Table S2. 33
Table S3. 34

MAIN SUPPLEMENTARY FIGURES 37
Figure S1-S3 37
Figure S4 40
Figure S5-S7 41
Figure S8 45

CRUDE ANALYSES 46
Figure S9-S11 46
Figure S12 49

Figure S13-S15 50
Figure S16 54

ADDITIONAL ANALYSES 55
Figure S17-S18 55
Figure S19-S20 58
Figure S21-S22 60
Figure S23-S24 63

REFERENCES 66

Study descriptions

British 1958 Birth Cohort

The British 1958 Birth Cohort (1958 BC) is a longitudinal population based cohort study that includes all births during one week in March in 1958 in England, Scotland and Wales [1]. Approximately 17,000 participants were recruited at birth and were subsequently followed up at ages 7, 11, 16, 23, 33, 42 and 45 years. At each follow-up, information on socioeconomic status, health and development, and familial and education factors were obtained. At 33 and 42 years, diet, lifestyle and occupational factors were also collected. Information on smoking status, asthma and hay fever were collected at the age of 42. At 45 years of age, 11,971 participants currently living in Britain were invited to take part in a biomedical survey, of whom 9,377 (78%) filled in a questionnaire and 8,302 (89%) also provided a blood sample, in which serum IgE concentration were measured and DNA were extracted for genotyping.

Genotyping
Genetic information was obtained from blood samples collected at 45 years, through two sub-studies from case-control studies that had used the 1958BC as a source for population controls: 3000 samples were randomly selected as part of the Wellcome Trust Case Control Consortium (WTCCC2 [2]) and 2592 distinct samples were randomly selected as part of the Type 1 Diabetes Genetics Consortium (T1DGC [3]). The WTCCC2 samples were genotyped on the Affymetrix 6.0 platform, whereas T1DGC samples were genotyped using the Illumina Infinium 550 K chip. The SNP rs16969968 was imputed both in T1DGC and in WTCCC2, with average posterior call rate >0·99 in both studies.

Hay fever, asthma, and allergic sensitization
At age 42, participants were asked whether they had ever had hay fever. There were 3·20% missing hay fever data (percentage of participants with missing information on the outcome among those with the genotyping information). At age 42, participants were asked whether they had ever had asthma. There were 3·22% missing asthma data (percentage of participants with missing information on the outcome among those with the genotyping information). Allergic sensitization was defined as specific IgE ≥0·30 kU/l in blood serum for any of the following 3 inhalant allergens including dust, cat and grass. Of note, specific IgE were only measured if total IgE was greater than 30 kU/l. There 3·33% missing data regarding allergic sensitization (percentage of participants with missing information on the outcome among those with the genotyping information).

Smoking status
Cigarette smoking was recorded at age 42 by Computer Aided Personal Interviewing, and was classified as never, ex- or current smoker. Reports of never smoking were verified using data from surveys at ages 23 and 33. Number of cigarettes smoked per day at 42 years was also reported for current smokers. Pipe and cigar smokers were excluded from analyses.
In the current analyses, we included 4,882 participants with rs1051730 genotype, allergic respiratory disease, and smoking data available.

Ethics
Written consent was obtained from participants for the use of information in medical studies. The 45-year biomedical survey and genetic studies were approved by the South-East Multi-Centre Research Ethics Committee (ref: 01/1/44) and the joint UCL/UCLH Committees on the Ethics of Human Research (Ref: 08/H0714/40).

Funding and acknowledgements
Data collection was funded by grant no. G0000934 from the UK Medical Research Council and cell-line creation by Wellcome Trust grant 068545/Z/02. This research used resources provided by the Type 1 Diabetes Genetics Consortium, a collaborative clinical study sponsored by the National Institute of Diabetes and Digestive and Kidney Diseases (NIDDK), National Institute of Allergy and Infectious diseases, National Human Genome Research Institute, National Institute of Child Health and Human Development, and Juvenile Diabetes Research Foundation International (JDRF) and supported by U01DK062418. This study makes use of data generated by the Wellcome Trust Case-Control Consortium. A full list of investigators who contributed to generation of the data is available from the Wellcome Trust Case-Control Consortium website. Funding for the project was provided by the Wellcome Trust under award 076113. Support was provided by the National Institute for Health Research Biomedical Research Centre at Great Ormond Street Hospital for Children NHS Foundation Trust and University College London.

Additional information
The analyses are adjusted for principal component. The observational or genetic data have previously been published.

ALSPAC

The Avon Longitudinal Survey of Parents and Children is a prospective cohort study which recruited pregnant women residing in Avon, United Kingdom, with expected dates of delivery between 1 April 1991 and 31 December 1992. Full details of the study recruitment and methodology have been published previously [4,5]. A total of 14,541 pregnancies were included in the initial sample, resulting in 14,062 live births and 13,988 children who were alive at one year of age. Detailed information on mothers and their partners (during and after pregnancy) and the children (since birth) has been collected from self-report questionnaires and attendance at clinics. Please note that the study website contains details of all the data that is available through a fully searchable data dictionary (http://www.bristol.ac.uk/alspac/researchers/data-access/data-dictionary/).

Funding and acknowledgements
The UK Medical Research Council and the Wellcome Trust (Grant ref: 092731) and the University of Bristol provide core support for ALSPAC. The genetic data for the ALSPAC mothers were funded by a grant from the Wellcome Trust (WT088806). Funding from British Heart Foundation, Cancer Research UK, Economic and Social Research Council, Medical Research Council, and the National Institute for Health Research, under the auspices of the UK Clinical Research Collaboration, is gratefully acknowledged. This work was supported by the Wellcome Trust (grant number 086684) and the Medical Research Council (grant numbers MR/J01351X/1, G0800612, G0802736, MC_UU_12013/1, MC_UU_12013/6). We are extremely grateful to all the families who took part in this study, the midwives for their help in recruiting them, and the whole ALSPAC team, which includes interviewers, computer and laboratory technicians, clerical workers, research scientists, volunteers, managers, receptionists and nurses.

ALSPAC Mothers

Genotyping

Rs1051730 was directly genotyped as part of the genomewide SNP genotyping using the Illumina human660W-quad array. Genotypes were called with Illumina GenomeStudio. PLINK (v1.07) was used to carry out quality control measures on an initial set of 10,015 subjects and 557,124 directly genotyped SNPs. A total of 8,340 subjects and 526,688 SNPs passed quality control filters. Further details of the genotyping methods have been published previously [6]. SNPs with more than 5% missingness were removed during quality control.

Hay fever, asthma, and allergic sensitization

Self-reported hay fever in the mothers was evaluated in a questionnaire completed when the study child was 97 months old. The mothers were asked “Have you ever had any of the following problems: hay fever. The choices were “Yes- had it recently (in the past year)”, “Yes, in the past, not recently”, or “No, never”. If the mothers had had hay fever recently or in the past, they were classified as having hay fever. If they answered never having had hay fever, they were classified as not having hay fever. Asthma was evaluated in the same way. The mothers were asked “Have you ever had any of the following problems: asthma. The choices were “Yes- had it recently (in the past year)”, “Yes, in the past, not recently”, or “No, never”. If the mothers had had asthma recently or in the past, they were classified as having asthma. If they answered never having had asthma, they were classified as not having asthma. Allergic sensitization was not determined.

Smoking status

The mothers’ self-reported smoking status was also evaluated in a questionnaire sent out when their participating children were 97 months old (on average). Women were asked whether they had ever smoked and whether they were currently smokers. Former and current smokers who reported not being daily smokers were excluded from the analyses. No questions were asked about cigar or pipe smoking. Smoking heaviness (cigarettes per day) was reported as a continuous variable. In the current analyses, we included 4,834 participants with rs1051730 genotype, allergic respiratory disease and smoking data available.

Ethics

Ethics approval for the study was obtained from the ALSPAC Ethics and Law Committee and the Local Research Ethics Committee.

Additional information

The analyses are not adjusted for principal component. The associations have not been published before.

ALSPAC Children

Genotyping

Participants (N=9,912) were genotyped using the Illumina HumanHap550 quad genome-wide SNP genotyping platform by 23andMe subcontracting the Wellcome Trust Sanger Institute, Cambridge, UK and the Laboratory Corporation of America, Burlington, NC, USA. Individuals were excluded from further analysis on the basis of having incorrect gender assignments; minimal or excessive heterozygosity, disproportionate levels of individual missingness (>3%); evidence of cryptic relatedness (>10% IBD) and being of non-European

ancestry. SNPs with more than 5% missingness were removed during quality control. After quality control, 8,365 unrelated individuals were available for analysis.

Hay fever, asthma, and allergic sensitization
Hay fever and asthma were evaluated when the participants were 18 years old. They completed a questionnaire with the question: "Have you ever had hay fever?". Likewise, participants completed a questionnaire with the question: "Have you ever had asthma?". Allergic sensitization was not determined.

Smoking status
At the age of 18 years, participants were asked about their lifetime smoking behaviour. From these, two categories of smoking status were created: never smokers and current daily smokers. Never smokers reported never having tried a cigarette in their lifetime and current daily smokers smoked at least one cigarette per day. Individuals reporting less frequent smoking were excluded from analyses. In the current analyses, we included 1,549 participants with rs1051730 genotype, allergic respiratory disease and smoking data available.

Ethics
Ethics approval for the study was obtained from the ALSPAC Ethics and Law Committee and the Local Research Ethics Committee.

Additional information
The analyses are not adjusted for principal component. The associations have not been published before.

COPSAC2000

The Copenhagen Prospective study on Asthma in Childhood (COPSAC2000) cohort is a population based single-center prospective clinical birth cohorts consisting of 411 children with phenotypic and genotypic information available in their parents. The children in the COPSAC2000-cohort were born to asthmatic mothers enrolled from the greater Copenhagen area in the period from August 1998 to December 2001. Non-missing phenotype data (from questionnaires or Doctors diagnosis) for parents of COPSAC2000 cohort (smoking, asthma, dermatitis, and allergic sensitization) and genotype data constituted 543 participants for the current study.

Genotyping
Genotyping of 951,117 genetic markers was carried out on the Illumina Infinium HumanOmniExpressExome bead chip at the AROS Applied Biotechnology AS center, in Aarhus, Denmark. Genotypes were called with Illumina's Genome Studio software. We excluded individuals with gender miss-matches, genetic duplicates, outlying heterozygosity $>0{\cdot}27$ and $<0{\cdot}037$, and those individuals not clustering with the CEU individuals (Utah residents with ancestry from northern and Western Europe) through a multi-dimensional clustering analyses (MDS) seeded with individuals from the International Hap Map Phase 3. SNP data was extracted from the Genome wide array data for the current study and the genotyping call rate was 99.8%.

Hay fever, asthma, and allergic sensitization
Evaluation of hay fever, asthma and allergic sensitization was done (an average) of 4 years before child birth. Participants with a doctor diagnosed hay fever or a positive answer to the question: "Have you ever had hay fever?" were considered to have hay fever. Participants with a doctor-diagnosed asthma were considered to have asthma. The serum levels were tested for IgE specificity, and allergic sensitization was defined as serum specific IgE positivity against allergens.

Smoking status
Smoking information was collected 2 years after child birth. Participants were divided into smokers and non-smokers since there was no information on former smoking. There was also no information on smoking heaviness.

Ethics
The study was conducted in accordance with the guiding principles of the Declaration of Helsinki and was approved by the Local Ethics Committee (COPSAC2000: KF 01-289/96, COPSAC2010: H-B-2008-093), and the Danish Data Protection Agency (COPSAC2000: 2008-41-1754, COPSAC2010: 2008-41-2599). Both parents gave written informed consent before enrolment.

Funding and acknowledgements
COPSAC is funded by private and public research funds all listed on www.copsac.com. The Lundbeck Foundation (Grant no R16-A1694); The Danish Ministry of Health (Grant no 903516); Danish Council for Strategic Research (Grant no 0603-00280B); The Danish Council for Independent Research (Grant no 10-082884 and271-08-0815); The Capital Region Research Foundation (No grant number) and NIH-NHLBI R01 HL129735 have provided core support for COPSAC. We express our gratitude to the children and families of the COPSAC cohorts for their support and commitment to our studies and acknowledge and appreciate the unique efforts of the COPSAC research team.

Additional information
The analyses are not adjusted for principal component. The observational or genetic data have previously been published [7].

The Dan-Monica10 study

In 1982–1984, a random sample of 4807 participants from the referral area of Glostrup County Hospital, Copenhagen, was invited to participate in the Danish MONICA I health survey [8]. The study was a part of an international World Health Organization (WHO) co-ordinated study, MONItoring of trends and determinants in CArdiovascular Diseases (MONICA). The sample was selected to represent an equal number of men and women born in 1922, 1932, 1942 or 1952 (age 30, 40, 50 and 60 years). A total of 226 Individuals, who were not of Danish nationality, were excluded. Of the remaining 4581 Danes, 3608 participated (79%). We used data from a follow-up examination performed in 1993-1994, called Dan-MONICA10. Since the first examination, 428 subjects had died and 23 had moved and could not be reached. The remaining 4130 Danes were invited to a new examination, when the participants were aged 41, 51, 61, or 71 years old. A total of 2656 (64%) participated in the Dan-MONICA10 follow-up study. The health examinations took

place a Research Centre for Prevention and Health, Glostrup. Participants completed a self-administered questionnaire on cardiovascular risk factors, medical history and lifestyle habits, including smoking and physical activity. A physical examination was performed by trained staff.

Hay fever, asthma, and allergic sensitization
Hay fever was defined as a positive answer to the question: “Has a doctor ever told you that you had allergic hay fever?” Asthma was defined as a positive answer to the question: “Has a doctor ever told you that you had asthma?”Allergic sensitisation, allergic sensitization, was defined by determination of serum specific IgE as described in previous studies [9-12]. In the Monica1 study, serum specific IgE positivity was tested using the ADVIA Centaur Allergy Screen assay (Bayer HealthCare Diagnostics division, Tarrytown, N.Y., USA) [13] that is a multi-allergen assay to detect specific serum IgE antibodies to 19 common inhalant allergens. Allergic sensitization was defined as one or more positive results according to the manufacturer’s instructions.

Smoking status
Information on smoking status was collected by a self-administered questionnaire to be filled in at home prior to the health examination. Smoking status was recorded as never, former, occasional (<1 cigarette, cheroot, cigar, or pipe per day) and daily smokers. Occasional smokers as well as daily smokers, who exclusively smoke cheroots, cigars, or pipe, were excluded from all analyses. Smoking heaviness among daily smokers was recorded as number of cigarettes per day. In the current study, we included 2,054 participants between 41 and 73 years of age.

Genotyping
Genotyping of rs1051730 was performed using KBiosciences allele-specific PCR (KASPar) (KBioscience, Hoddesdon, UK).

Ethics
Ethics approval was given by the local research ethics committee. All participants gave written consent and the study was conducted in accordance with the Second Helsinki Declaration.

Funding and acknowledgements
The Dan-MONICA10 was sponsored by The Danish Heart Foundation; the Danish Medical Research Council; The Danish Hospital Foundation of Medical Research, region of Copenhagen, the Faroe Islands and Greenland; The Danish Health Insurance Foundation; The Foundation of E. & M. Wedel-Wedellsborg; Landsforeningen til Bekæmpelse af Kredsløbssygdomme; The Augustinus Foundation; The Becket Foundation; and The Foundation of senior registrar J. & L. Boserup.

Additional information
The analyses are not adjusted for principal component. The genetic data used in this study have not previously been published.

ELSA
The English Longitudinal Study of Ageing (ELSA) is a population based national cohort of participants (48% men) aged over 50 years recruited from the Health Surveys for England in

1998, 1999 and 2001 as previously described [14]. The sample has been followed up every 2 years and data have been collected via computer-assisted personal interviews and self-completion questionnaires. A wide range of phenotypic measures relevant to ageing are available. These measures were done at Wave 0 of the study (1998, 1999 and 2001) and at follow up (2004/5). Data on health behaviors and a wide range of health outcomes are available. Nearly all participants (97%) have agreed to let us collect other register based data which allows for the assessment of health outcomes and cause specific mortality. More information can be found at http://www.ifs.org.uk/elsa/.

Genotyping
In Wave 2 (2004/2005) of the study, 5,633 participants provided blood samples for DNA extraction. Genotyping was performed by KBioscience using in-house KaSPAR technology.

Hay fever, asthma, and allergic sensitization
Whether the participants had asthma or not was evaluated at wave 1, where they were asked: "Has a doctor ever told you that you have/have had any of the following conditions (asthma)?". Hay fever and allergic sensitization were not determined.

Smoking status
Individuals were classified as current smokers if they reported smoking at least one cigarette per day or at least one gram of tobacco per day on weekdays. Former smokers were individuals who reported ever having smoked cigarettes but who were not current smokers. Never smokers were individuals who had never reported smoking cigarettes. Current or former pipe and cigar smokers who did not also smoke cigarettes were excluded from all analyses. In the current analyses, we included 5,263 participants with rs16969968 genotype and smoking data available.

Ethics
ELSA has been approved by the National Research Ethics Service and all participants have given informed consent.

Funding and acknowledgements
ELSA is funded by the National Institute on Aging in the US (R01 AG017644; R01AG1764406S1) and by a consortium of UK Government departments (including: Department for Communities and Local Government, Department for Transport, Department for Work and Pensions, Department of Health, HM Revenue and Customs and Office for National Statistics).

Additional information
The analyses are not adjusted for principal component. The results have not been published previously.

FINRISK

The National Finland Cardiovascular Risk Study (FINRISK) is a large population survey on risk factors of non-communicable diseases in Finland [15]. Every five years since 1972, area, sex and age stratified random samples of population have been drawn from the Population Register. In these analyses, data from FINRISK 1992, 1997, 2002 and 2007 surveys were used. Age range of the participants was from 25 to 64 years in study years 1992 and 1997 and from 25 to 74 years in study years 2002 and 2007. Surveys have included a self-administered questionnaire, physical examination and blood draw for laboratory analyses and extraction of DNA.

Genotyping
DNA was derived from whole blood samples, which were frozen immediately at the clinical study sites. The samples were transferred to the National Institute of Health and Welfare, where the DNA was extracted. Genotyping of rs16969968 (CHRNA5 D398N) was done under standard protocols of iPLEX Gold technology on the MassARRAY System (Sequenom, San Diego, CA, USA). The success rate was >0·99 and it was in HWE. Minor allele frequency was 0·32.

Hay fever, asthma, and allergic sensitization
Diagnoses of hay fever and asthma were based on self-report. Participants who answered "last week", "last month" or "last year" to the question: "When have you last used hay fever medication" were classified as having hay fever. Likewise, participants with a positive answer to the question: "Has a doctor ever told you that you had asthma?" were classified as having asthma. Data on hay fever was available in FINRISK 1997, 2002 and 2007 surveys, whereas asthma data was available in FINRISK 1992 survey as well. Allergic sensitization was not determined.

Smoking status
In the same questionnaire, respondents were asked whether they had ever smoked. Those stating that they had never smoked were categorized as never smokers and skipped the other smoking-related questions. Ever smokers were defined as those who had smoked at least 100 cigarettes in their lifetime. More questions were used to classify ever smokers as current and former smokers. Former smokers reported having been either regular or occasional smokers but were not smoking currently. For the current analyses, only those who had quit over 6 months ago were included in the former smoker category. Current smokers reported regular or daily smoking having smoked on the day of the assessment or the previous day. Exclusive pipe or cigar smokers were excluded from all analyses. In order to create a variable for smoking quantity the participants were asked to indicate the average number of both manufactured and self-rolled cigarettes they smoked or had smoked per day before quitting. Manufactured and self-rolled cigarettes were totalled for the analysis.

Ethics
Surveys have obtained permissions from the local Ethics Committees that have varied over time, such as the Coordinating Ethics Committee for the Uusimaa Hospital District in 2007. From 1997 onwards a written informed consent has been obtained from each participant.

Funding
This study was supported by the Academy of Finland Center of Excellence in Complex Disease Genetics (grant numbers 213506, 129680), the Academy of Finland (grant numbers

139635, 129494, 136895, 263836 and 141054), the Sigrid Juselius Foundation, and ENGAGE – European Network for Genetic and Genomic Epidemiology, FP7-HEALTH-F4-2007, grant agreement number 201413. Finnish Foundation for Cardiovascular Research.

Additional information
The analyses are not adjusted for principal component. The data concerning the smoking associated SNP and allergy have not been published previously.

GOYA Females
The GOYA females consist of mothers from a birth cohort randomly selected according to body mass index (BMI) distribution. The GOYA females were derived from the Danish genome-wide association study GOYA (Genomics of Overweight in Young Adults), nested within the Danish National Birth Cohort (DNBC) [16,17]. The Danish National Birth Cohort (DNBC) is a collection of data on 92,274 pregnant women recruited between 1996 and 2002, from their first antenatal visit to their general practitioner. Women participated in four telephone interviews (16 and 30 weeks gestation and 6 and 18 months after birth) and in a questionnaire-based follow-up 7 years after birth. They also provided two blood samples during pregnancy. The GOYA females used in this study were drawn as a random cohort sample from the 67,863 women within the DNBC who provided information about pre-pregnancy BMI, gave birth to a live born singleton infant and provided a blood sample during pregnancy. This comprised 2,542 women.

Genotyping
Genotyping of rs1051730 was carried out on the Illumina Human610-Quad v1.0 BeadChip (545,350 SNPs) at the Centre National de Génotypage (CNG), Evry, France. Call rate>0·99.

Hay fever, asthma, and allergic sensitization
Women were asked at app. 16 weeks gestation if they had ever had allergic rhinitis diagnosed by a doctor (0·6% missing). Women were asked at app. 16 weeks gestation if they had ever had asthma diagnosed by a doctor (5·3% missing). Allergic sensitization was not determined.

Smoking status
Women were asked about their present smoking status and any smoking during pregnancy at app. 16 weeks of gestation. Women were therefore classified as ’current’ (current + any smoking in pregnancy) or ’never/former’ smokers (no smoking at any time in pregnancy). In the current analyses, there were 2,016 participants with data on rs1051730 genotype and smoking habits and of these, 2,009 women had information on asthma and 1,897 had information on hay fever available.

Ethics
The study was approved by the regional scientific ethics committee and by the Danish Data Protection Board. All participants provided written informed consent.

Funding and acknowledgements
GOYA (Genomics of Obesity in Young Adults) was sampled as a case-cohort study within the Danish National Birth Cohort. The establishment of this cohort was based on a major grant from the Danish National Research Foundation. Additional support for the Danish National Birth Cohort was obtained from the Pharmacy Foundation, the Egmont Foundation, the March of Dimes Birth Defects Foundation and the Augustinus Foundation. Genotyping

for the GOYA Study was funded by the Wellcome Trust (Grant ref: 084762MA). The MRC Integrative Epidemiology Unit is supported by the Medical Research Council (MC_UU_12013/1-9) and the University of Bristol.

Additional information
The analyses are not adjusted for principal component. The data concerning the smoking associated SNP and allergy has not been published previously.

GOYA Males

Genomics of Overweight in Young Adults (GOYA) males is a longitudinal case-cohort (obese, non-obese) study comprising a randomly (1%) selected control group and all extremely overweight men identified among 362,200 Caucasian men examined at the mean age of 20 years at the draft boards in Copenhagen and its surrounding areas during 1943–1977. Obesity was defined as 35% overweight relative to a local standard in use at the time (mid 1970's), corresponding to a BMI ≥31·0 kg/m2, which proved to be above the 99th percentile. All of the obese and 50% of the random sampled controls, who were still living in the region, were invited to a follow-up survey in 1992–94 at the mean age of 46 years, at which time the blood samples were taken and genotyping were performed for a total of 673 extremely overweight and 792 controls. With a sampling fraction of 0·5% (50% of 1%), the controls represent about 158,000 men among whom the case group was the most obese. In the current study, information from cohort part comprising 789 individuals with non-missing data was utilized.

Genotype
Genome-wide genotyping on the Illumina 610 k quad chip was carried out at the Centre National de Ge´notypage (CNG), Evry, France. We excluded SNPs with minor allele frequency, 1%, 0.5% missing genotypes or which failed an exact test of Hardy-Weinberg equilibrium (HWE) in the controls. We also excluded any individual who did not cluster with the CEU individuals (Utah residents with ancestry from northern and western Europe) in a multidimensional scaling analysis seeded with individuals from the International HapMap release 22. Rs1051730 was extracted from the GWAS dataset with a call rate of 99·9%.

Hay fever, asthma, and allergic sensitization
Asthma was evaluated by questionnaire with the question: ”Does food, medicine, or grass give you asthma?” Hay fever was evaluated by the question: “Does food, medicine or grass give you hay fever?” Allergic sensitization was not determined.

Ethics
The study was approved by the regional scientific ethics committee and the Danish Data Protection Board with consent from the participants.

Funding and acknowledgements
The GOYA study was conducted as part of the activities of the Danish Obesity Research Centre (DanORC, www.danorc.dk) and The MRC centre for Causal Analyses in Translational Epidemiology (MRC CAiTE). The genotyping for GOYA was funded by the Wellcome Trust (WT 084762). GOYA is a nested study within The Danish National Birth Cohort which was established with major funding from the Danish National Research Foundation. Additional support for this cohort has been obtained from the Pharmacy

Foundation, the Egmont Foundation, The March of Dimes Birth Defects Foundation, the Augustinus Foundation, and the Health Foundation. TSA was supported by the Gene Diet Interactions in Obesity (GENDINOB, www.gendinob.dk) postdoctoral fellowship grant. LP is funded by an MRC Population Health Scientist Fellowship (MR/J012165/1).

Additional information
The analyses are not adjusted for principal components, but ethnic outliers and related individuals were already excluded during post genotyping quality control. Some data were previously published [16].

Health2006

The Health2006 study took place during 2006-2008 and consisted of a random sample of 7,931 Danish (Danish nationality and born in Denmark) men and women aged 18-69 years invited to participate in a health examination [18]. A total of 3,471 (43·8%) participated. Potential participants living in the Copenhagen area were identified in the central Danish Civil Registration System, and then recruited by invitation. The aim was to investigate the prevalence and risk factors of chronic diseases such as mental health, asthma, allergies, cardiovascular disease, and diabetes.

Genotyping
Blood samples were taken from all participants as part of their health examination. The buffy coat was frozen for DNA extraction, and later genomic DNA was extracted using a Qiagen AutoPure LS system. Genotyping was performed using KBiosciences allele-specific PCR (KASPar) (KBiosciences, Hoddesdon, UK). The call rate for this SNP (rs1051730) was > 99·2%. No errors were observed in 370 duplicate samples.

Hay fever, asthma, and allergic sensitization
We used the ADVIA Centaur sIgE assay (Bayer Corporation, New York, NY) to test serum specific IgE to mite (Dermatophagoides [D.] pteronyssinus), grass, cat, and birch[19]. The specific IgE analysis was positive if the measurement was ≥0·35 kU/l. Allergic sensitization, allergic sensitization, was defined as one or more positive tests for specific IgE against the allergens. Classification of hay fever and asthma was done by the questions: ”Has a doctor ever told you that you had/have hay fever?” and ”Has a doctor ever told you that you had/have asthma”.

Smoking status
Information on smoking status was collected by a self-administered questionnaire to be filled in at home prior to the health examination. Smoking status was recorded as never, former, occasional (<1 cigarette, cheroot, cigar, or pipe per day) and daily smokers. Occasional smokers as well as daily smokers smoking exclusively cheroots, cigars, or pipe were excluded from all analyses. Smoking heaviness among daily smokers was recorded as number of cigarettes per day. In the current analyses, we included 3143 participants with rs1051730 genotype, allergic respiratory disease and smoking data available.

Ethics
The Health2006 study was approved by the Ethical Committee of Copenhagen (KA-20060011) and the Danish Data Protection Agency. Informed written consent was obtained from all participants..

Funding and acknowledgements
The Health2006 study was financially supported by grants from the Velux Foundation; the Danish Medical Research Council, Danish Agency for Science, Technology and Innovation; the Aase and Ejner Danielsens Foundation; ALK-Abello´ A/S (Hørsholm, Denmark), Timber Merchant Vilhelm Bangs Foundation, MEKOS Laboratories (Denmark) and Research Centre for Prevention and Health, the Capital Region of Denmark.

Additional information
The analyses are not adjusted for principal component. The data concerning the smoking associated SNP and allergy has not been published previously.

Health2008

The Health2008 study is a study of health and chronic disease initiated in 2008 and completed in 2009 [20-22]. Participants were recruited from the Danish Central Personal Register as random samples of the background population living in the Western part of the Copenhagen Region [22]. The total of 2218 persons 30-60 years of age was invited. Pregnant women, persons with known diabetes, chronic obstructive pulmonary disease, cardiovascular disease, hypertension, a history of blood clots, or unable to participate in physical activities such as climbing stairs were excluded from the study. Thus, a total of 795 participated (participation rate 36%). All studies included comprehensive questionnaire and interview data as well as clinical and biochemical data [22].

Genotyping
DNA was extracted from blood samples taken from all participants as part of their health examination. Genotyping was performed with the Illumina Human Exome BeadChip (version 1.2) using the Illumina HiScan (Illumina, San Diego, CA). Genotypes were called using the genotyping module (version 1.9.4) of GenomeStudio software (version 2011.1; Illumina). The call rate for this SNP (rs16969968) was > 99%.

Hay fever, asthma, and allergic sensitization
Classification of hay fever and asthma was done by the questions: ”Has a doctor ever told you that you had hay fever?” and ”Has a doctor ever told you that you had asthma”. In the Health2008 study, we used the ADVIA Centaur sIgE assay (Bayer Corporation, New York, NY) to test serum specific IgE to mite (Dermatophagoides [D.] pteronyssinus), grass, cat, and birch. Allergic sensitization was defined as serum specific IgE positivity to one or more of the tested allergens.

Smoking status
Information on smoking status was collected by a self-administered questionnaire to be filled in at home prior to the health examination. Smoking status was recorded as never, former, occasional (<1 cigarette, cheroot, cigar, or pipe per day) and daily smokers. Occasional smokers as well as daily smokers smoking exclusively cheroots, cigars, or pipe were excluded from all analyses. Smoking heaviness among daily smokers was recorded as number of cigarettes per day. In the current analyses, we included 618 participants with rs16969968 genotype, allergic sensitization and smoking data available.

Ethics

The study was approved by the Ethics Committee of Copenhagen and the Danish Data Protection Agency. We followed the recommendations of the Declaration of Helsinki, and each participant gave informed written consent.

Funding and acknowledgements
This work was supported by the Timber Merchant Vilhelm Bang's Foundation, the Danish Heart Foundation (Grant number 07-10-R61-A1754-B838-22392F), and the Health Insurance Foundation (Grant number 2012B233). Tea Skaaby was supported by a grant from the Lundbeck Foundation (Grant number R165-2013-15410).

Additional information
The analyses are not adjusted for principal component. The data concerning the smoking associated SNP and allergy has not been published previously.

HUNT2

The second wave of the HUNT Study in Norway (HUNT 2) took place in 1995-97, where all adults aged 20 years and older in Nord Trøndelag County were invited to participate. A total of 65,237 (70%) accepted the invitation and gave written informed consent to use the data for medical research. The data collection included questionnaires, clinical measurements and blood samples (http://www.ntnu.edu/hunt/data/que).

Genotyping
Altogether 56,664 participants were genotyped for the rs1051730 single nucleotide polymorphism variant. DNA was extracted from blood samples for all participants of the HUNT 2 study and stored at the HUNT biobank. The rs1051730 polymorphism was genotyped at the HUNT biobank using TaqMan genotyping assays (Applied Biosystems, Foster City, CA, USA) and performed on an Applied Biosystems 7900HT Fast real-Time PCR System using 10 ng of genomic DNA. The call rate cut-off was set to 90%. The genotyping success rate was 98·6% and quality score for each individual genotype was >90 (mean 99·7). Genotype frequencies were in agreement with HapMap data.

Hay fever, asthma, and allergic sensitization
Hay fever was defined as a confirmative answer to the question: ”Do you have hay fever or nasal allergies?” Asthma was defined as a confirmative answer to the question: ”Do you have or have you had asthma?”. Allergic sensitization was not determined.

Smoking status
Smoking status was measured with self-completed questionnaire data with a categorical variable, and the participants were classified as never smokers, former smokers or current smokers. Current smokers were asked how many cigarettes they smoked per day, the age when starting to smoke and possibly age for smoking cessation. Exclusive pipe and/or cigar smokers were excluded from the analyses. In the current analyses, we included 43,211 participants between the ages 19 to 101 years with rs1051730 genotype, allergic respiratory disease and smoking data available.

Ethics

Use of data in the present study was approved by the Regional Committee for Medical Research Ethics (Reference nr. 2013/1127/REK midt). Participants gave written informed consent.

Funding and acknowledgements
Nord-Trøndelag Health Study (The HUNT Study) is a collaboration between HUNT Research Centre (Faculty of Medicine, Norwegian University of Science and Technology NTNU), Nord-Trøndelag County Council and the Norwegian Institute of Public Health.

Additional information
The analyses were not adjusted for principal component. Associations between the SNP and e.g., lung cancer have previously been published [23].

Inter99

The Inter99 study is a randomised controlled trial (CT00289237, ClinicalTrials.gov) investigating the effects of lifestyle intervention on CVD (N=61,301) [24]. We used baseline data from a random subsample of 12,934 men and women aged approximately 30, 35, 40, 45, 50, 55, or 65 years invited to participate in a health examination during 1999-2001. Participants were living in the Copenhagen area and were identified in the central Danish Civil Registration System, and recruited by invitation. Only participants with a Northern European origin (Denmark, Norway, Sweden, Iceland, and Faeroe Islands) were included in the present study.

Genotyping
DNA was extracted from blood samples taken from all participants as part of their health examination. Genotyping was performed using KBiosciences allele-specific PCR (KASPar) (KBiosciences, Hoddesdon, UK). The call rate for this SNP (rs1051730) was > 98·8%. No errors were observed in 353 duplicate samples.

Hay fever, asthma, and allergic sensitization
Asthma was defined as a positive answer to the question: “Has a doctor ever told you that you had asthma?” Serum samples were analyzed for specific IgE to mite (D. pteronyssinus), grass, cat, and birch by the IMMULITE 2000 Allergy Immunoassay System [25]. The specific IgE analysis was positive if the measurement was ≥0·35 kU/l. Allergic sensitization, allergic sensitization, was defined as a positive tests for specific IgE against any of allergens. Hay fever was not determined.

Smoking status
Information on smoking status was collected by a self-administered questionnaire to be filled in at home prior to the health examination. Smoking status was recorded as never, former, occasional (<1 cigarette, cheroot, cigar, or pipe per day) and daily smokers. Occasional smokers as well as daily smokers smoking exclusively cheroots, cigars, or pipe were excluded from all analyses. Smoking heaviness among daily smokers was recorded as number of cigarettes per day. In the current analyses, we included 4,991 participants with rs1051730 genotype, asthma, allergic sensitization, and smoking data available.

Ethics

Informed written consent was obtained from all participants. The study was approved by the Ethical Committee of Copenhagen.

Funding and acknowledgements
Data collection in the Inter99 study was supported economically by The Danish Medical Research Council, The Danish Centre for Evaluation and Health Technology Assessment, Novo Nordisk, Copenhagen County, The Danish Heart Foundation, The Danish Pharmaceutical Association, Augustinus foundation, Ib Henriksen foundation and Becket foundation.

Additional information
The analyses are not adjusted for principal component. The data concerning the smoking associated SNP and allergy has not been published previously.

KORA
The Cooperative Health Research in the Region of Augsburg (KORA) study is a population based case-control study. The study participants were recruited from the third MONICA survey (S3) which was conducted in 1994-1995 in Augsburg, Germany. The objective and protocols of the MONICA surveys have been previously described [26]. Briefly, four cross-sectional health surveys (MONICA S1 to S4) were performed in the population aged 25-74 years of the city of Augsburg and two surrounding counties. In total, 4856 participants were recruited in the S3 survey in 1994/1995. The study used for these analyses is a nested case-control study comprising 1537 participants, which was performed between September 1997 and December 1998 and in which cases were defined by sensitization status (SPT/RAST). Details of the sampling frame and study design have been published earlier [27].
Genotyping
The study participants underwent a standardized medical examination including blood draw. Genotyping was performed on the Illumina Omni 2.5 and the Illumina Omni Express platform. Genotypes were called with Genome Studio and annotated to NCBI build 37. The call rate for this SNP (rs1051730) was ≥ 98%. (Before imputation, SNPs with call rates <98% were excluded. Imputation was performed with IMPUTE v2.3.0 using the 1000G phase 1 (v3) reference panel) [28].

Hay fever, asthma, and allergic sensitization
Information on hay fever was requested using a self-administered questionnaire. The participants were asked whether hay fever was ever diagnosed by a doctor. Information on asthma was requested using a self-administered questionnaire. The participants were asked whether asthma was ever diagnosed by a doctor. Allergen specific IgE antibodies to common aeroallergens (grass and birch pollen, housedust mite, cat and *Cladosporium*) were determined by the fluorescence enzyme immunoassay technique (CAP-FEIA, Pharmacia, Uppsala, Sweden). Allergic sensitization was defined as serum specific IgE sensitivity (≥ 0·35 kU/l) against at least one inhalant allergen.

Smoking status
Information on smoking status was collected using a self-administered questionnaire. Smoking status was defined based on the following three questions: 1) “Do you currently smoke cigarettes?”, 2) “Have you ever smoked?” and 3) “Do you smoke regularly?” Current smoker, if the participant answered yes to questions 1) and 3); former smoker, if the participant answered no to question 1), but yes to question 2) and never smoker, if the

participant answered no to questions 1) and 2). Information on smoking heaviness was requested by the question “How many cigarettes per day?”.

Ethics
The study was approved by the ethics committee of the Bavarian Medical Association, and written informed consent was obtained from each participant.

Funding
The KORA study was initiated and financed by the Helmholtz Zentrum München – German Research Center for Environmental Health, which is funded by the German Federal Ministry of Education and Research (BMBF) and by the State of Bavaria. Furthermore, KORA research was supported within the Munich Center of Health Sciences (MC-Health), Ludwig-Maximilians-Universität, as part of LMUinnovativ.

Additional information
The analyses were not adjusted for principal component. The data has not been published before.

MIDSPAN Family Study

The Middle-aged Span-of-Life (MIDSPAN) Family Study is an offspring cohort of one of the original MIDSPAN cohorts. It is one of four MIDSPAN population cohort studies based in Scotland [29]. The three original studies took place between 1964 and 1976. Twenty years later, in 1996, the next generation was studied when offspring of couples in the original Renfrew/ Paisley Study were recruited into the Family Study. This latter group is the subject of the present analysis. Details of the study have been described previously [30]. All 2,120 participants used in the current project are of European ancestry and have data on rs1051730 genotype, smoking habits, age, sex, asthma and hay fever.

Genotyping
Genotyping was performed on an ABI PRISM 7900HT sequence detection system using a Taqman assay (Assay ID: C_9510307_20, Applied Biosystems), followed by allelic discrimination using software from Applied Biosystems (SDS V2.0).3.

Hay fever, asthma, and allergic sensitization
Classification of participant hay fever was done by the following question: “Do you suffer from, or have you ever suffered from hay fever? (Yes/No)”. Participants were classified as having asthma if they answered “Yes” to the question: “Do you suffer from, or have you ever suffered from asthma?” as well as “Yes” to at least one of the following two questions: “Have you suffered from an asthma attack in the last 12 months?” and “Are you currently taking medication (puffers or inhalers) for asthma?” Allergic sensitization was not determined.

Smoking status
The participants completed a questionnaire which included questions on smoking habit. Three categories of smoking habit were defined: never smoker, current smoker, and former smoker.

Ethics

Ethics approval was obtained from the Argyll and Clyde Health Board Local Research Ethics Committee.

Funding
The MIDSPAN Family Study was funded by The Wellcome Trust, and the NHS Research and Development Cardiovascular Research Programme. Participants gave their informed consent.

Additional information
The analyses are not adjusted for principal component. Data on the rs1051730 SNP have previously been published [31-35].

The NEO study

The NEO study was designed for extensive phenotyping to investigate pathways that lead to obesity-related diseases [36]. The NEO study is a population-based, prospective cohort study that includes 6,671 individuals aged 45–65 years, with an oversampling of individuals with overweight or obesity (BMI > 27 kg/m^2). At baseline, information on demography, lifestyle, and medical history has been collected by questionnaires. In addition, samples of 24-h urine, fasting and postprandial blood plasma and serum, and DNA were collected. Participants underwent an extensive physical examination, including anthropometry, electrocardiography, spirometry, and measurement of the carotid artery intima-media thickness by ultrasonography. In random subsamples of participants, magnetic resonance imaging of abdominal fat, pulse wave velocity of the aorta, heart, and brain, magnetic resonance spectroscopy of the liver, indirect calorimetry, dual energy X-ray absorptiometry, or accelerometry measurements were performed. The collection of data started in September 2008 and completed at the end of September 2012. Participants are currently being followed for the incidence of obesity-related diseases and mortality.

Genotyping
Genotyping was performed using the Illumina HumanCoreExome chip, which was subsequently imputed to the 1000 genome reference panal. Genotyping calling algorithm is GenCall. Genotyping and SNP call rates >98%.

Hay fever, asthma, and allergic sensitization
Participants were defined as having asthma if they had the general practitioner record code R96 (asthma) according to the International Classification of Primary Care. Hay fever and allergic sensitization were not determined.

Smoking status
Questionnaires on health and lifestyle factors were sent to all participants. Smoking status was classified as never-smoker, former smoker, or current smoker on the basis of a questionnaire, in which the participants could answer the question “Do you smoke?” as either: “No, I never smoked”; “No, but I did smoke in the past”; or “Yes, currently”. Long-term tobacco exposure was expressed in pack years of smoking, calculated by multiplying the number of packs of cigarettes smoked per day by the number of years the participant smoked.

Ethics
The Medical Ethical Committee of the Leiden University Medical Center (LUMC) approved the design of the study. All participants gave their written informed consent.

Funding and acknowledgements
The authors of the NEO study thank all individuals who participated in the Netherlands Epidemiology in Obesity study, all participating general practitioners for inviting eligible participants and all research nurses for collection of the data. We thank the NEO study group, Pat van Beelen, Petra Noordijk and Ingeborg de Jonge for the coordination, lab and data management of the NEO study. The genotyping in the NEO study was supported by the Centre National de Génotypage (Paris, France), headed by Jean-Francois Deleuze. The NEO study is supported by the participating Departments, the Division and the Board of Directors of the Leiden University Medical Center, and by the Leiden University, Research Profile Area Vascular and Regenerative Medicine. Dennis Mook-Kanamori is supported by Dutch Science Organization (ZonMW-VENI Grant 916.14.023).

Additional information
The analyses are adjusted for principal components (4). The data has not been published previously.

NSHD

The Medical Research Council National Survey of Health and Development (NSHD) is an on-going prospective population based birth cohort study consisting of all births in England, Scotland and Wales in one week in March 1946 [37]. The sample includes single births to married mothers whose fathers were in non-manual or agricultural occupations and a randomly selected one in four of all others, whose fathers were in manual labor. The original cohort, now 70 years of age, comprised 2,547 women and 2,815 men who have been followed-up over 20 times since their birth. The data collected to date include repeat cognitive function, physical, lifestyle and anthropomorphic measures, as well as blood analytes and other measures. In 2006-10 the cohort carried out a particularly intensive phase of clinical assessment and biological sampling with blood and urine sampling and analysis, and cardiac and vascular imaging [38].

Genotyping
DNA was extracted from blood samples collected in 1999 [39]. Genotyping of rs16969968 was carried out by LGC Genomics (Hoddesdon, UK; www.lgcgenomics.com) using fluorescence-based competitive allele-specific PCR (KASPar). Call rate was >95%.

Hay fever, asthma, and allergic sensitization
The study has data on self-reported hay fever and self-reported asthma. The outcome hay fever variable was derived from responses to questions about hay fever asked by research nurses at home visits in 1989 (age 43) and 1999 (age 53). In 1989, cohort members were asked whether they had ever had hay fever, and in 1999 they were asked whether they had had it in the last 10 years. The asthma variable was derived from responses to questions about asthma asked by the research nurses at home visits in 1989 and 1999. In 1989, cohort members were asked whether they had ever had asthma, and in 1999 they were asked whether they had had it in the last 10 years. Allergic sensitization was not determined.

Smoking status
Smoking status was collected during a home interview in 1999 at age 53 years by trained interviewers [40]. Current cigarette smoking status (“yes”, “no”) and the number of cigarettes

smoked per day was obtained. Study members who provided an affirmative response to being current cigarette smokers, regardless of the quantity of cigarettes smoked per day, were classified as "smokers", while those who provided a negative response were classified as "non-smoker". Pipe and cigar smokers who did not also report cigarette smoking were excluded from analyses. In the current analyses, we included 2,484 participants with rs16969968 genotype, allergic respiratory disease and smoking data available.

Additional information
The analyses are not adjusted for principal component. Some of the observational or genetic data have previously been published in CARTA papers or elsewhere [41].

Ethics
Ethical approval was given by the Central Manchester Research Ethics Committee, and the participants gave informed written consent.

Funding and acknowledgements
We are very grateful to the members of this birth cohort for their continuing interest and participation in the study. We would like to acknowledge the Swallow group at University College London, who performed the DNA extractions. This work was funded by the Medical Research Council [MC_UU_12019/1].

The 1936 Cohort

The 1936 Cohort is a longitudinal population-based study based on a random sample of 1,200 persons living in 1976 (aged 40 years at the time of the study) and living in 4 municipalities (Broendby, Glostrup, Herlev, and Ledoeje-Smoerum) of Copenhagen drawn from the Danish Civil Registration. They were invited by a letter for a health examination that focused on risk factors for cardiovascular disease. Enclosed was a questionnaire regarding medical history and health and lifestyle to be completed in advance. Between 1976 and 1977, a total of 1,052 participants were examined (participation rate=87·7%). In 1995–1996 all participants were invited for a re-examination where a total of 695 were examined (participation rate=66%). We use these re-examination data in the current study.

Genotyping
DNA was extracted and purified from leukocytes (LGC Genomics, Hoddlesdon, UK). All participants were genotyped with the Illumina Infinium HumanCoreExome-12 BeadChip (CoreExomeChip) using HiScan system (Illumina) at the Novo Nordisk Foundation Centre for Basic Metabolic Research, University of Copenhagen, Copenhagen, Denmark. The standard pipeline in Illumina Genome Studio software was used for the genotype calling.
A total of 538,448 markers on 684 individuals entered the Quality Control (QC) pipeline, where 9886 markers were removed due to a call-rate below 95% before QC on individuals could begin. 28 individuals were excluded due to: 1) a call-rate below 95%, 2) extreme positive or negative inbreeding coefficients 3) ethnic outliers using Principal Component Analysis (PCA) on ancestral markers, 4) unknown pedigree relation found by Identical By Descent (IBD) analysis, where the individual with the lowest call rate for each pedigree-pair was removed, 5) sample duplicates, and finally 6) sex discrepancy.

We used a combination of scripts written in Python (v2.7.3) and R (v3.0.1) together with the PLINK (v1.07) software in our QC pipeline. After QC, 656 individuals with 528,562 markers were ready for imputation.

Additional genotypes were imputed with 1,000 Genomes haplotypes Phase I integrated variant set release (SHAPE2) in NCBI build 37 (hg19) coordinates, using IMPUTE2 [42] on all markers that passed a Hardy-Weinberg Equilibrium (HWE) filter (pHWE<0·005), into the 1000 genomes phase 1 panel [43].

Hay fever, asthma, and allergic sensitization

The questionnaire used in 1995–1996 (at 60 years of age) included the following questions on atopic diseases: “Has a doctor ever told you that you had hay fever?” (self-reported hay fever if they answered “yes”)” and “Has a doctor ever told you that you had asthma?” (self-reported asthma if they answered “yes”).

Measurement of aeroallergen sensitization was performed using the ADVIA Centaur® Allergy Screen assay (Bayer HealthCare Diagnostics division, Tarrytown, N.Y., USA) that is a multi-allergen assay for the qualitative detection of specific serum IgE antibodies specific to common inhalant allergens in serum. The test includes a total of 19 common inhalant allergens. Allergic sensitization was defined as a positive result of the dichotomized assay output.

Smoking status

Smoking status assessed at the re-examination in 1995-1996 was classified as ”never smokers”, “former smokers”, and “current smokers” according to the answers to the questions “Do you smoke?” and “”If you don’t smoke now, have you smoked before?”. Smoking heaviness was calculated as the sum of self-reported cigarettes with and without filter. In the current analyses, we included 557 participants with rs1051730 genotype, allergic respiratory disease, and smoking data available.

Ethics

The study was conducted according to the principles of the Declaration of Helsinki. It was approved by the Local Ethics Committee, and participants gave written informed consent.

Additional information

The analyses are not adjusted for principal component. The current associations have not been previously published.

UK Biobank

The UK Biobank is a large prospective study with over 500,000 participants from across the United Kingdom and aged 40–69 years at recruitment in 2006–2010 [44]. The study has both data from questionnaires, physical measures, sample assays, accelerometry, multimodal imaging, genome-wide genotyping and longitudinal follow-up for a large number of health-related outcomes. In the current study, we use the interim UK Biobank genetic data that comprise more than 150,000 samples. We have restricted the analyses to individuals genetically defined as Caucasian and to participants that are unrelated.

In additional analyses, we excluded the approximately one third of the participants who participated in the UK BiLEVE Study because they were selected according to smoking habits and lung function [45].

Genotyping

Approximately 450,000 of the participants have been/are being genotyped using the UK Biobank Axiom array from Affymetrix. There are approximately 800,000 markers on this array. The other approximately 50,000 samples were genotyped on the closely related UK BiLEVE array. These are two very similar arrays with more than 95% common marker content.

The rs16969968 SNP was directly genotyped and did not demonstrate evidence for deviation from Hardy Weinberg Equilibrium. The analysis sample was restricted to unrelated individuals, based on a threshold of 0·05 estimated from genetic kinships, and to individuals of Caucasian genetic ancestry using principal components analyses (PCA).

Hay fever, asthma, and allergic sensitization

Hay fever was defined as a positive answer to the question: ”Has a doctor ever told you that you have had any of the following conditions? Hayfever, allergic rhinitis, or eczema”. Asthma was defined as a positive answer to the question: “Has a doctor ever told you that you have had any of the following conditions? Asthma” Of note, the data includes information about age at first diagnosis which enabled us to exclude participants below 16 years at diagnosis in additional analyses. Allergic sensitization was not determined.

Smoking status

Participants were asked about current and past tobacco (cigarette, pipe, cigar or other) smoking behavior in a computerized questionnaire. A full list of the questions is available at: http://biobank.ctsu.ox.ac.uk/crystal/docs/TouchscreenQuestionsMainFinal.pdf. Two questions were asked about current and past smoking status: “Do you smoke tobacco now?” (Yes, on most or all days, Only occasionally, No, Prefer not to answer) and “In the past, how often have you smoked tobacco?” (Smoked on most or all days, Smoked occasionally, Just tried once or twice, I have never smoked, Prefer not to answer). From the answers to these two questions, individuals were categorized as current (current daily or occasional smokers), former (past daily or occasional smokers) or never smokers (individuals who had never tried tobacco or had smoked tobacco once or twice).

Additional information

The analyses are adjusted for 15 principal components. The allergy- and smoking-associated SNP associations have not been previously published.

Ethics

Each participant has given informed consent. An independent Ethics and Governance Council oversees adherence to the Ethics and Governance Framework [44].

Funding and acknowledgements

UK Biobank has received funding from the UK Medical Research Council, Wellcome Trust, Department of Health, British Heart Foundation, Diabetes UK, Northwest Regional Development Agency, Scottish Government, and Welsh Assembly Government. As described in the manuscript, the MRC and Wellcome Trust played a key role in the decision to establish UK Biobank, a large, population-based, prospective, open access resource that would allow detailed investigations of the genetic and environmental determinants of the diseases of middle and old age. The MRC, Wellcome Trust, Department of Health, and Scottish Chief Scientist Office each have a representative on the UK Biobank Board. The MRC and Wellcome Trust fund the independent Ethics and Governance Council [44].

Whitehall II

Whitehall II is a cohort study with recruitment of 10,308 participants (70% men) between 1985 and 1988 involved 20 London based Civil service departments [83]. Genetic samples were collected in 2004 from over 6,000 participants. The study is highly phenotyped for cardiovascular and other ageing related health outcomes, with 9 phases of follow up (5 with clinical assessment and biological sampling), over 20 years of follow up. A wide variety of health behaviour and environmental data are also collected and the participants are consented for linkage to recorded clinical data such as Hospital Episode Statistics (HES), the Office of National Statistics mortality data and the national registry of acute coronary syndromes in England and Wales (Myocardial Ischaemia National Audit Project).

Genotyping
Genotyping of rs16969968 was performed as part of genotyping using the Metabochip [46]

Hay fever, asthma, and allergic sensitization
Hay fever and asthma were measured at phase 1. Hay fever was defined as a positive answer to the question: "There are some kinds of health problems that keep recurring and some that people have all the time. In the last 12 months have you suffered from any of the following health problems? Hay fever" Asthma was defined as a positive answer to the question: "There are some kinds of health problems that keep recurring and some that people have all the time. In the last 12 months have you suffered from any of the following health problems? Asthma" Allergic sensitization was not determined.

Smoking status
Information on smoking status was collected by questionnaire during the first phase of data collection. Individuals who reported smoking cigars or pipes but not cigarettes were excluded from all analyses.

Additional information
The analyses are not adjusted for principal components.

Ethics
Ethical approval for the Whitehall II study was obtained from the University College London Medical School committee on the ethics of human research. Informed consent was gained from every participant.

Funding and acknowledgements
The Whitehall II study has been supported by grants from the Medical Research Council (K013351); British Heart Foundation; Health and Safety Executive; Department of Health; National Heart Lung and Blood Institute (NHLBI: HL36310) and National Institute on Aging (AG13196), US, NIH; Agency for Health Care Policy Research (HS06516); and the John D and Catherine T MacArthur Foundation Research Networks on Successful Midlife Development and Socio-economic Status and Health.

SHIP

The Study of Health in Pomerania (SHIP) is a population-based cohort study in the German region of West Pomerania. SHIP was at first planned as a cross-sectional study [47]. Examinations were performed in centers located at Stralsund and Greifswald between the 16th of October 1997 and the 19th of May 2001. SHIP-0 had a response of 68·8%. The response in women was slightly higher (69·4%) than in men (68·2%). Participation in the different age groups for women ranged from 76·6% (participants aged between 50 and 60) to 49·5% (participants aged between 70 and 80) and for men from 74·3% (participants aged between 50 and 60) to 63·2% (participants aged between 70 and 80) [48].

Genotyping
Genotyping was performed using the Human SNP 6.0 Array (Affymetrix, Santa Clara, CA, USA). Hybridization of genomic DNA was genotyped according to the manufacturer's standard recommendations. Genotypes were determined using the Birdseed2 clustering algorithm. All remaining arrays had a sample call rate > 92 %.

Hay fever, asthma, and allergic sensitization
Participants were classified as having hay fever if they answered confirmatory to the question: "Do you sometimes or all the time suffer from hay fever?" The participants were defined as having allergic asthma or not according to self-reported questionnaire information. Allergic sensitization was not determined.

Smoking status
The participants completed an interview-based questionnaire which included questions on smoking habit. Three categories of smoking habits were defined: never, former and current smokers.

Additional information
The analyses are not adjusted for principal components.

Ethics
SHIP was planned and accompanied with support and advice from an external Data Safety and Monitoring Committee (DSMC). Each participant gave written informed consent. The study conformed to the principles of the Declaration of Helsinki and was approved by the Ethics Committee of the University of Greifswald.

Funding and acknowledgements
The Study of Health in Pomerania (SHIP) is part of the Research Network of Community Medicine (www.community-medicine.de).

SHIP TREND

SHIP-TREND is the second cohort of the population project Study of Health in Pomerania (SHIP). Baseline data were collected between 2008 and 2012 [48].

Genotyping
Genotyping was performed using the Human SNP 6.0 Array (Affymetrix, Santa Clara, CA, USA). Hybridization of genomic DNA was genotyped according to the manufacturer's standard recommendations. Genotypes were determined using the Birdseed2 clustering algorithm. All remaining arrays had a sample call rate > 92 %.

Hay fever, asthma, and allergic sensitization
In the standardized interview, participants were asked whether a physician had ever diagnosed them with allergy, and if so, which type of allergy. Participants who answered "allergy to house dust mite" or "pollen allergy" were defined as having hay fever in the current study. A diagnosis of lung asthma was defined as the participants that reported to have bronchial asthma. Allergic sensitization was not determined.

Smoking status
The participants completed an interview-based questionnaire which included questions on smoking habit. Three categories of smoking habits were defined: never, former and current smokers.

Additional information
The analyses are not adjusted for principal components.

Ethics
SHIP was planned and accompanied with support and advice from an external Data safety and Monitoring Committee (DSMC). Each participant gave written informed consent. The study conformed to the principles of the Declaration of Helsinki and was approved by the Ethics Committee of the University of Greifswald.

Funding and acknowledgements
The Study of Health in Pomerania (SHIP) is part of the Research Network of Community Medicine (www.community-medicine.de).

Main supplementary tables

Table S1. Descriptive statistics of study populations.

						Hay fever		Asthma		Allergic sensitization	
	**Smoking status**	**Smoking heaviness**	**N**	**% Male**	**Median age (IQR)**	**N**	**%**	**N**	**%**	**N**	**%**
1958 BC	Never		2,308	50·6	42 (42, 42)	535	23·2	186	8·1	473	20·5
	Former		1,400	51·9	42 (42, 42)	264	18·9	123	8·8	233	16·6
	Current	X	1,174	47·3	42 (42, 42)	168	14·3	97	8·3	161	13·7
	Total		4,882	50·1	42 (42, 42)	967	19·8	406	8·3	867	17·8
ALSPAC Children	Never		1,275	39·8	18·6 (18·2,19)	532	41·7	283	22·2	NA	NA
	Current	X	274	32·1	18·7 (18·2,19·1)	133	48·5	77	28·1	NA	NA
	Total		1,549	38·4	18·6 (18·2,19)	665	42·9	360	23·2	NA	NA
ALSPAC Mothers	Never		2,715	0	37·2 (35·1,40·1)	1,068	39,3	433	16·0	NA	NA
	Former		1,238	0	38·1 (35·1,41·1)	478	38,6	198	16·0	NA	NA
	Current	X	881	0	35·3 (32·2,39·1)	298	33,8	160	18·2	NA	NA
	Total		4,834	0	37·1 (34·2,40·1)	1,844	38,1	791	16·4	NA	NA
COPSAC2000	Never		412	43·0	33·1 (29·6, 36·5)	237	57·5	262	63·6	217	52·7
	Current		131	53·4	32·4 (28·9, 36·9)	56	42·8	73	55·8	56	42·8
	Total		543	45·5	33·0 (29·3, 36·6)	293	54·0	335	61·7	273	50·3
Dan-Monica10	Never		614	36·8	51·8 (42·1, 61·7)	80	13·0	31	5·1	115	18·7
	Former		514	63·4	61·4 (51·3, 71·2)	65	12·6	48	9·3	89	17·3
	Current	X	926	46·9	51·8 (41·9, 61·6)	88	9·5	63	6·8	172	18·6
	Total		2,054	48·0	52·0 (42·0, 61·8)	233	11·3	142	6·9	376	18·3
ELSA	Never		1,816	33·3	62 (55, 70)	NA	NA	200	11·0	NA	NA
	Former		2,606	54·6	64 (56, 72)	NA	NA	326	12·5	NA	NA
	Current	X	841	45·1	59 (54, 66)	NA	NA	88	10·5	NA	NA
	Total		5,263	45·7	62 (55, 70)	NA	NA	614	11·7	NA	NA
FINRISK*	Never		13,631	35·3	49 (37,59)	NA	NA	653	4·8	NA	NA
	Former		5,308	60·9	53 (42, 62)	NA	NA	286	5·4	NA	NA
	Current	X	6,424	57·1	44 (34,53)	NA	NA	227	3·5	NA	NA
	Total		25,363	46·2	48 (37,59)	NA	NA	1,166	4·6	NA	NA
FINRISK**	Never		9,909	36·1	50 (38,61)	1,550	15·6	NA	NA	NA	NA
	Former		4,106	61·0	54 (44, 63)	596	14·5	NA	NA	NA	NA
	Current	X	4,567	57·5	45 (35,54)	633	13·9	NA	NA	NA	NA
	Total		18,582	46·9	49 (38,60)	2,779	15·0	NA	NA	NA	NA
GOYA Females***	Never		1,492	0	29·7 (27·1, 32·6)	272	19·5	131	8·8	NA	NA
	Current		524	0	28·5 (25·6, 32·0)	68	13·6	51	9·8	NA	NA
	Total		2,016	0	29·5 (26·7, 32·5)	340	17·9	182	9·1	NA	NA
GOYA Males	Never		178	100	44 (40, 49)	35	19·7	12	6·7	NA	NA
	Former		217	100	48 (42, 54)	24	11·1	7	3·2	NA	NA
	Current	X	394	100	47 (42, 55)	42	10·7	16	4·1	NA	NA
	Total		789	100	46 (41, 53)	101	12·8	35	4·4	NA	NA
Health2006	Never		1,363	44·5	48 (38, 60)	280	20·5	138	10·1	375	27·5
	Former		1,063	45·7	53 (44, 61)	192	18·1	134	12·6	215	20·2

	Current	X	717	40·2	50 (41, 58)	99	13·8	70	9·8	140	19·5
	Total		3,143	43·9	50 (41, 60)	571	18·2	342	10·9	730	23·2
Health2008	Never		279	47·7	44·0 (38·2,51·8)	76	27·2	37	13·3	93	33·3
	Former		218	39·9	50·1 (42·9, 55·1)	37	17·0	18	8·3	53	24·3
	Current	X	121	45·4	48·0 (40·6, 54·5)	16	13·2	17	14·5	20	16·5
	Total		618	44·5	46·8 (40·6, 53·6)	129	20·9	72	11·6	166	26·9
HUNT2	Never		19,353	39·5	47 (33,64)	3,903	20·2	1,451	7·5	NA	NA
	Former		11,241	57·4	53 (42,67)	2,175	19·4	1,078	9·6	NA	NA
	Current	X	12,617	44·3	46 (36,57)	2,146	17·0	1,109	8·8	NA	NA
	Total		43,211	45·6	48 (36,63)	8,224	19·0	3,638	8·4	NA	NA
Inter99	Never		1,825	45·6	45·0(39·9,50·2)	NA	NA	176	9·6	690	37·8
	Former		1,347	50·8	49·7(40·1,54·8)	NA	NA	112	8·3	427	31·7
	Current	X	1,819	49·2	45·1 (40·0, 50·2)	NA	NA	151	8·3	565	31·1
	Total		4,991	48·3	45·1 (40·0, 50·3)	NA	NA	439	8·8	1,682	33·7
KORA	Never		369	39·6	45 (28, 76)	84	22·8	21	5·7	176	47·8
	Former		259	54·1	46 (29, 73)	56	21·6	19	7·3	123	47·5
	Current	X	209	54·6	41 (29, 60)	32	15·3	13	6·2	103	49·6
	Total		837	47·8	44 (28, 76)	172	20·6	53	6·3	402	48·1
MIDSPAN	Never		1003	42·6	44 (40, 49)	240	23·9	50	5·0	NA	NA
	Former		578	49·3	47 (42, 50)	116	20·1	30	5·2	NA	NA
	Current	X	539	44·0	45 (40, 49·5)	86	16·0	23	4·3	NA	NA
	Total		2120	44·8	45 (41, 49)	442	20·8	103	4·9	NA	NA
NEO	Never		1892	42·9	56 (50, 61)	NA	NA	211	11·2	NA	NA
	Former		2778	49·1	57 (53, 62)	NA	NA	315	11·3	NA	NA
	Current	X	831	57·3	55 (50, 60)	NA	NA	61	7·3	NA	NA
	Total		5501	48·2	57 (51, 61)	NA	NA	587	10·7	NA	NA
NSHD	Never		733	42·7	53 (53, 53)	194	26·5	83	11·3	NA	NA
	Former		1,186	53·4	53 (53, 53)	283	23·9	116	9·8	NA	NA
	Current	X	565	49·2	53 (53, 53)	118	20·9	52	9·2	NA	NA
	Total		2,484	49·3	53 (53, 53)	595	23·9	251	10·1	NA	NA
The 1936 Cohort	Never		201	34·3	60·5 (60·2, 60·8)	20	10·0	18	9·0	26	12·9
	Former		154	62·3	60·5 (60·2, 60·8)	20	13·0	8	5·2	27	17·5
	Current	X	202	50·5	60·5 (60·2, 60·8)	9	4·5	14	6·9	24	11·9
	Total		557	47·9	60·5 (60·2, 60·8)	49	8·8	40	7·2	77	13·8
UK Biobank	Never		61,315	43·6	57 (50, 63)	15,268	24·9	7,726	12·6	NA	NA
	Former		38,296	50·6	60 (53, 64)	8,075	21·1	4,801	12·5	NA	NA
	Current	X	13,344	49·0	55 (48, 61)	2,320	17·4	1·521	11·4	NA	NA
	Total		112,955	49·0	58 (51, 63)	25,663	22·7	14,048	12·4	NA	NA
Whitehall II	Never		1,218	71·9	43 (39, 49)	242	19·9	45	3·7	NA	NA
	Former		730	79·2	44 (40, 49)	133	18·2	31	4·3	NA	NA
	Current	X	351	72·9	43 (39, 49)	46	13·1	10	2·9	NA	NA
	Total		2,299	74·4	43 (39, 49)	421	18·3	86	3·7	NA	NA
SHIP	Never		1,428	29·0	54 (39, 64)	134	9·4	157	11·0	NA	NA
	Former		1,374	65·4	56 (42, 68)	110	8·0	181	13·2	NA	NA

	Current		1,225	54·5	40 (31, 51)	76	6·2	130	10·6	NA	NA
	Total		4,027	49·1	50 (36, 63)	320	8·0	468	11·6	NA	NA
SHIP TREND	Never		419	32·7	53 (41, 63)	55	13·1	17	4·1	NA	NA
	Former		351	56·1	53 (44, 63)	55	15·7	13	3·7	NA	NA
	Current		214	45·3	43 (34, 51)	31	14·5	11	5·1	NA	NA
	Total		984	43·8	50 (40, 61)	141	14·3	41	4·2	NA	NA

[*]For asthma. [**] For hay fever. [***] A total of 2,016 women had information on asthma or hay fever, whereas only 2,009 and 1,897, respectively, had information on asthma and hay fever.

Abbreviations: 1958 BC, British 1958 Birth Cohort; ALSPAC, Avon Longitudinal Survey of Parents and Children; COPSAC2000, Copenhagen Prospective study on Asthma in Childhood; ELSA, English Longitudinal Study of Ageing; FINRISK, Finland Cardiovascular Risk Study; GOYA, Genomics of extremely Overweight in Young Adults; HUNT, Nord-Trøndelag Health Study; Inter99, Intervention 1999; IQR, interquartile range; KORA, Cooperative Health Research in the Region of Augsburg; MIDSPAN, the Middle-aged Span-of-Life; Monica, Monitoring of trends and determinants in Cardiovascular Diseases; NA, not available; NEO, Netherlands Epidemiology of Obesity; NSHD, National Survey of Health and Development; SHIP, Study of Health in Pomerania; UK Biobank, United Kingdom Biobank.

Table S2. Allele frequencies for rs1051730/rs16969968 in the CARTA studies

Study	SNP	Major homozygotes	Heterozygotes	Minor homozygotes	MAF	HW, p-value	Total
1958 BC	rs16969968	2,191	2,167	596	0·34	0·25	4,882
ALSPAC Children	rs1051730	675	705	169	0·34	0·45	1,549
ALSPAC Mothers	rs1051730	2,160	2,144	530	0·33	0·95	4,834
COPSAC2000	rs1051730	243	240	60	0·33	0·95	543
Dan-Monica10	rs1051730	915	900	239	0·34	0·61	2,054
ELSA	rs16969968	2,400	2,296	567	0·33	0·61	5,263
FINRISK*	rs16969968	11,527	11,159	2,677	0·33	0·76	25,363
GOYA Females	rs1051730	217	902	897	0·33	0·66	2,016
GOYA Males	rs1051730	348	340	101	0·34	0·21	789
Health2006	rs1051730	1,411	1,394	338	0·33	0·82	3,143
Health2008	rs16969968	287	268	63	0·32	0·97	618
HUNT2	rs1051730	19,294	19,039	4,878	0·33	0·080	43,211
Inter99	rs1051730	2,195	2,242	554	0·34	0·61	4,991
KORA	rs1051730	321	416	100	0·37	0·053	837
MIDSPAN	rs1051730	964	939	217	0·32	0·58	2,120
NEO	Rs1051730	2,588	2,385	528	0·31	0·82	5,501
NSHD	rs16969968	1,160	1,081	243	0·32	0·70	2,484
The 1936 Cohort	rs1051730	243	262	52	0·33	0·12	557
UK Biobank	rs1051730	49,911	50,524	12,520	0·33	0·12	112,955
Whitehall II	rs16969968	1,063	1,008	233	0·32	0·79	2,304
SHIP	rs16969968	1,743	1,784	500	0·35	0·19	4,027
SHIP TREND	rs16969968	452	419	113	0·33	0·30	984

* For asthma. The study population for hay fever is smaller, please see Table S1.

Abbreviations: 1958 BC, British 1958 Birth Cohort; ALSPAC, Avon Longitudinal Survey of Parents and Children; COPSAC2000, Copenhagen Prospective study on Asthma in Childhood; ELSA, English Longitudinal Study of Ageing; FINRISK, Finland Cardiovascular Risk Study; GOYA, Genomics of extremely Overweight in Young Adults; HW, Hardy Weinberg Equilibrium; HUNT, Nord-Trøndelag Health Study; Inter99, Intervention 1999; KORA, Cooperative Health Research in the Region of Augsburg; MIDSPAN, the Middle-aged Span-of-Life; MAF, minor allele frequency, Monica, Monitoring of trends and determinants in Cardiovascular Diseases; NEO, Netherlands Epidemiology of Obesity; NSHD, National Survey of Health and Development; SHIP, Study of Health in Pomerania; UK Biobank, United Kingdom Biobank.

Table S3.

Study-specific measures of hay fever, asthma and allergic sensitization.

Study	Hay fever	Asthma	Allergic sensitisation
1958 BC	A positive answer to the question: “Have you ever had hay fever?”	A positive answer to the question: “Have you ever had hay asthma?”	Serum specific IgE positivity against inhalant allergens
ALSPAC Children	A positive answer to the question: “Have you ever had hay fever?”	A positive answer to the question: “Have you ever had asthma?”	NA
ALSPAC Mothers	Answering ”Yes- had it recently (in the past year)” or ”Yes, in the past, not recently” to the question: ” Have you ever had any of the following problems: hay fever?”	Answering ”Yes- had it recently (in the past year)” or ”Yes, in the past, not recently” to the question: ” Have you ever had any of the following problems: asthma?”	NA
COPSAC2000	Doctor-diagnosed hay fever or a positive answer to the question: “Have you ever had hay fever?”	Doctor-diagnosed asthma	Serum specific IgE positivity against inhalant allergens
Dan-Monica10	A positive answer to the question: “Has a doctor ever told you that you had allergic hay fever?”	A positive answer to the question: “Has a doctor ever told you that you had asthma?”	Serum specific IgE positivity against inhalant allergens
ELSA	NA	A positive answer to the question: “Has a doctor ever told you that you have/have had any of the following conditions (asthma)?”	NA
FINRISK	Answering last week, last month or last year to the question: “When have you last used hay fever medication”	A positive answer to the question: “Has a doctor ever told you that you had asthma?”	NA
GOYA Females	A positive answer to the question: “Has a doctor ever told you that you had allergic rhinitis?”	A positive answer to the question: “Has a doctor ever told you that you had asthma?”	NA
GOYA Males	A positive answer to the question: “Does food, medicine or grass give you hay fever”	A positive answer to the question: “Does food, medicine, or grass give you asthma?”	NA
Health2006	A positive answer to the question: “Has a doctor ever told you that you had hay fever?”	A positive answer to the question: “Has a doctor ever told you that you had asthma?”	Serum specific IgE positivity against inhalant allergens
Health2008	A positive answer to the question: “Has a doctor ever told you that you had hay fever?”	A positive answer to the question: “Has a doctor ever told you that you had asthma?”	Serum specific IgE positivity against inhalant allergens
HUNT2	A positive answer to the question: “Do you have hay fever or nasal allergies?”	A positive answer to the question: “Do you have or have you had asthma?”	NA
Inter99	NA	A positive answer to the question: “Has a doctor ever told you that you had asthma?”	Serum specific IgE positivity against inhalant allergens
KORA	A positive answer to the question: “Has a doctor	A positive answer to the question: “Has a doctor	Serum specific IgE positivity against inhalant

	ever told you that you had hay fever?”	ever told you that you had asthma?”	allergens
MIDSPAN	A positive answer to the question: “Do you suffer from, or have you ever suffered from hay fever?”	A positive answer to the question: “Do you suffer from, or have you ever suffered from asthma?” and a positive answer to at least one of the following two questions: “Have you suffered from an asthma attack in the last 12 months?” and “Are you currently taking medication (puffers or inhalers) for asthma?”	NA
NEO	NA	Asthma recorded by general practitioner	NA
NSHD	A positive answer to the question: “Have you ever had hay fever?” or “Have you had hay fever in the last 10 years?”	A positive answer to the question: “Have you ever had asthma?” or “Have you had asthma in the last 10 years?”	NA
The 1936 Cohort	A positive answer to the question: “Has a doctor ever told you that you had hay fever?”	A positive answer to the question: “Has a doctor ever told you that you had asthma?”	Serum specific IgE positivity against inhalant allergens
UK Biobank	A positive answer to the question: ”Has a doctor ever told you that you have had any of the following conditions? Hay fever, allergic rhinitis or eczema”	A positive answer to the question: “Has a doctor ever told you that you have had any of the following conditions? Asthma“	NA
Whitehall II	A positive answer to: “In the last 12 months have you suffered from any of the following health problems? Hay fever”	A positive answer to: “In the last 12 months have you suffered from any of the following health problems? Asthma”	NA
SHIP	A confirmative answer to the question: “Do you sometimes or all the time suffer from hay fever?”	The participants were defined as having allergic asthma or not according to self-reported questionnaire information.	NA
SHIP TREND	Self-reported doctor-diagnosed “allergy to house dust mite” or “pollen allergy”	Participants who reported to have bronchial asthma	NA

Abbreviations: 1958 BC, British 1958 Birth Cohort; ALSPAC, The Avon Longitudinal Survey of Parents and Children; COPSAC2000, Copenhagen Prospective study on Asthma in Childhood; ELSA, the English Longitudinal Study of Ageing; FINRISK, Finland Cardiovascular Risk Study; GOYA, Genomics of extremely Overweight in Young Adults; HUNT, Nord-Trøndelag Health Study; IgE, immunoglobulin E; ICPC, International Classification of Primary Care; Inter99, Intervention 1999; KORA, The Cooperative Health Research in the Region of Augsburg; MIDSPAN, the Middle-aged Span-of-Life; Monica, Monitoring of trends and determinants in Cardiovascular Diseases; NA, not available; NEO, Netherlands Epidemiology of Obesity; NSHD, National Survey of Health and Development; SHIP, Study of Health in Pomerania; UK Biobank, United Kingdom Biobank.

Main supplementary figures

Figure S1-S3

Age- and sex- adjusted association of smoking status with hay fever, asthma and allergic sensitization.

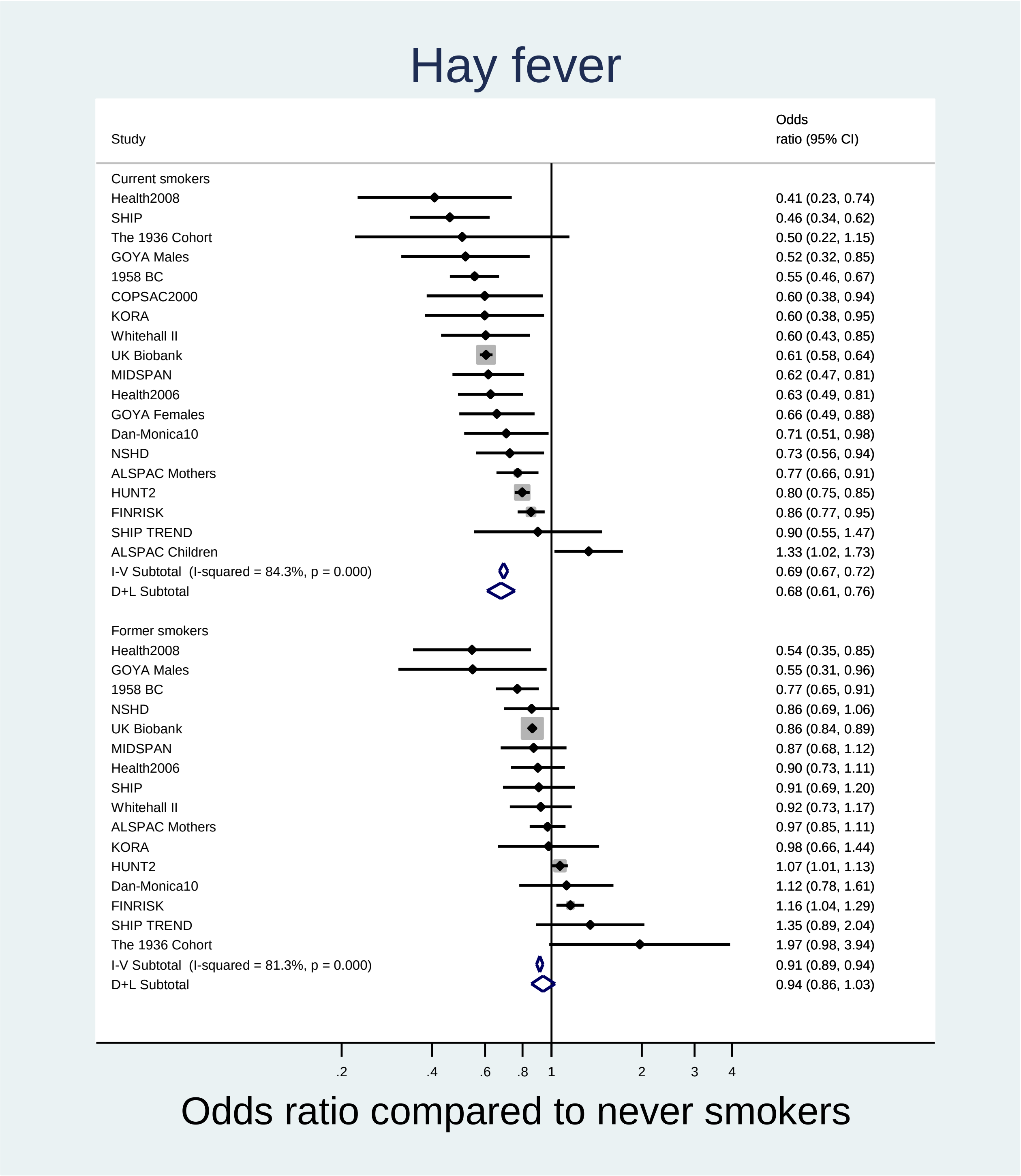

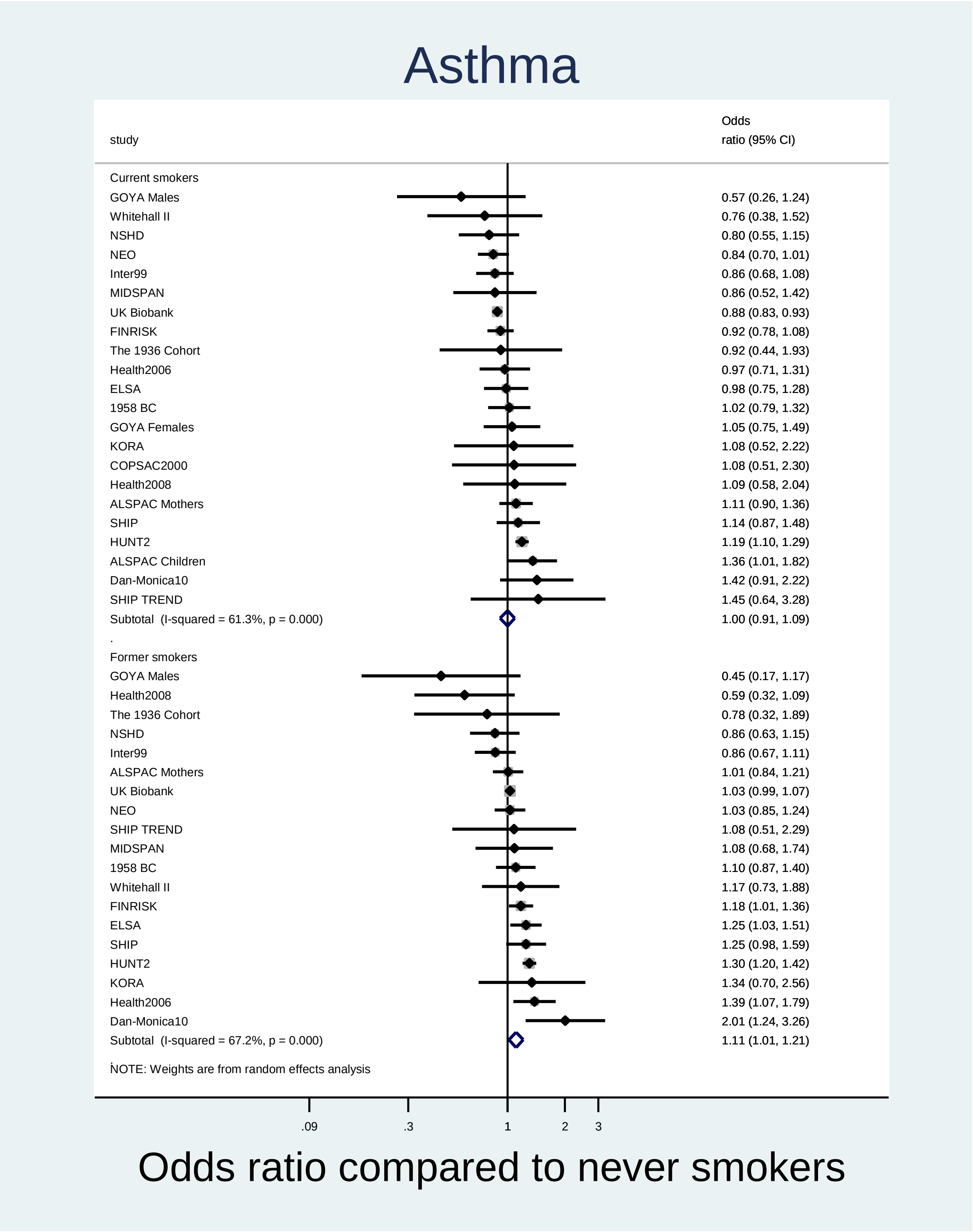
Asthma
study
Odds
ratio (95% CI)
Current smokers
GOYA Males 0.57 (0.26, 1.24)
Whitehall II 0.76 (0.38, 1.52)
NSHD 0.80 (0.55, 1.15)
NEO 0.84 (0.70, 1.01)
Inter99 0.86 (0.68, 1.08)
MIDSPAN 0.86 (0.52, 1.42)
UK Biobank 0.88 (0.83, 0.93)
FINRISK 0.92 (0.78, 1.08)
The 1936 Cohort 0.92 (0.44, 1.93)
Health2006 0.97 (0.71, 1.31)
ELSA 0.98 (0.75, 1.28)
1958 BC 1.02 (0.79, 1.32)
GOYA Females 1.05 (0.75, 1.49)
KORA 1.08 (0.52, 2.22)
COPSAC2000 1.08 (0.51, 2.30)
Health2008 1.09 (0.58, 2.04)
ALSPAC Mothers 1.11 (0.90, 1.36)
SHIP 1.14 (0.87, 1.48)
HUNT2 1.19 (1.10, 1.29)
ALSPAC Children 1.36 (1.01, 1.82)
Dan-Monica10 1.42 (0.91, 2.22)
SHIP TREND 1.45 (0.64, 3.28)
Subtotal (I-squared = 61.3%, p = 0.000) 1.00 (0.91, 1.09)
.
Former smokers
GOYA Males 0.45 (0.17, 1.17)
Health2008 0.59 (0.32, 1.09)
The 1936 Cohort 0.78 (0.32, 1.89)
NSHD 0.86 (0.63, 1.15)
Inter99 0.86 (0.67, 1.11)
ALSPAC Mothers 1.01 (0.84, 1.21)
UK Biobank 1.03 (0.99, 1.07)
NEO 1.03 (0.85, 1.24)
SHIP TREND 1.08 (0.51, 2.29)
MIDSPAN 1.08 (0.68, 1.74)
1958 BC 1.10 (0.87, 1.40)
Whitehall II 1.17 (0.73, 1.88)
FINRISK 1.18 (1.01, 1.36)
ELSA 1.25 (1.03, 1.51)
SHIP 1.25 (0.98, 1.59)
HUNT2 1.30 (1.20, 1.42)
KORA 1.34 (0.70, 2.56)
Health2006 1.39 (1.07, 1.79)
Dan-Monica10 2.01 (1.24, 3.26)
Subtotal (I-squared = 67.2%, p = 0.000) 1.11 (1.01, 1.21)
.
NOTE: Weights are from random effects analysis
.09
.3
1
2
3
Odds ratio compared to never smokers

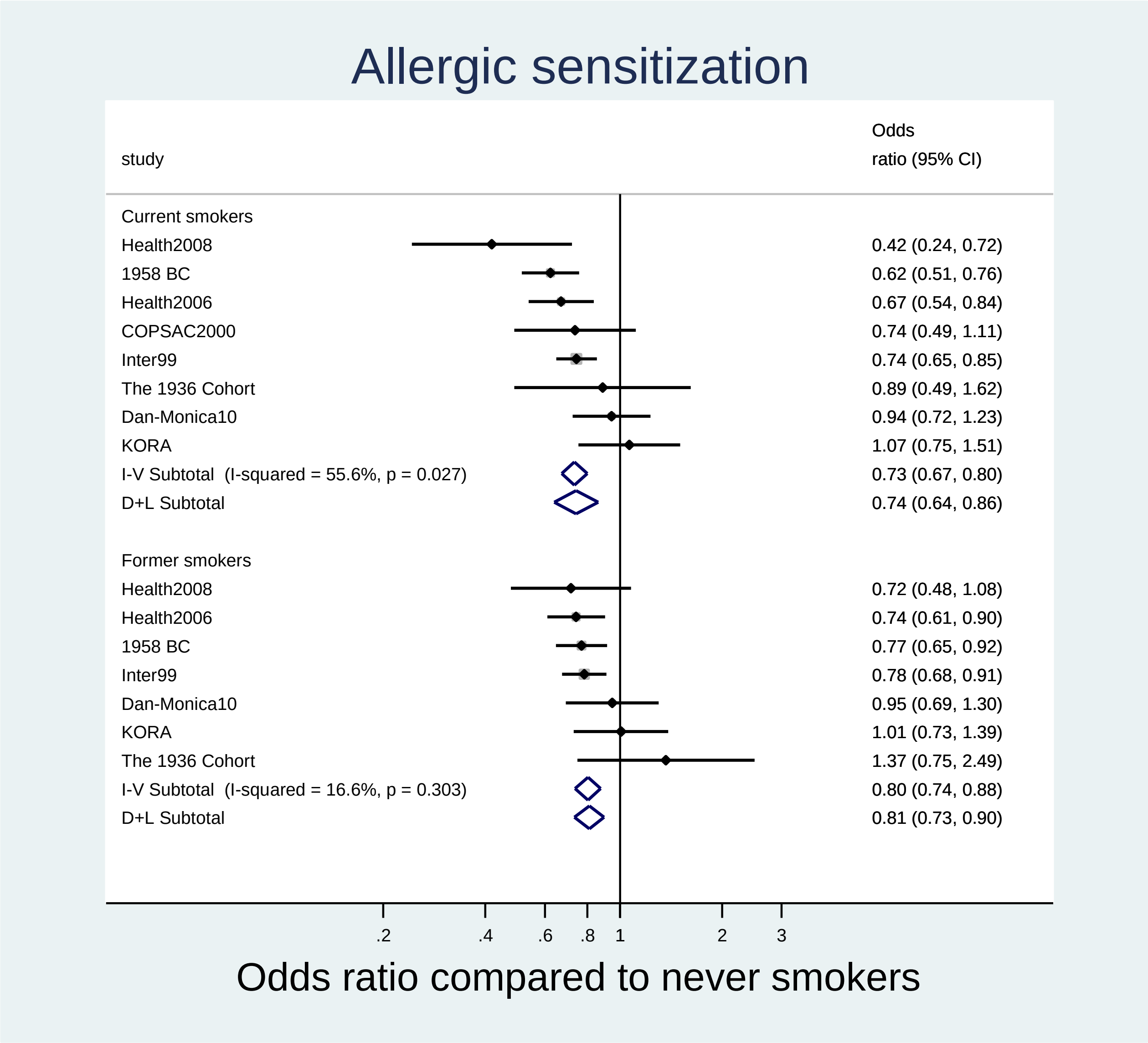
Allergic sensitization
study
Odds ratio (95% CI)
Current smokers
Health2008 0.42 (0.24, 0.72)
1958 BC 0.62 (0.51, 0.76)
Health2006 0.67 (0.54, 0.84)
COPSAC2000 0.74 (0.49, 1.11)
Inter99 0.74 (0.65, 0.85)
The 1936 Cohort 0.89 (0.49, 1.62)
Dan-Monica10 0.94 (0.72, 1.23)
KORA 1.07 (0.75, 1.51)
I-V Subtotal (I-squared = 55.6%, p = 0.027) 0.73 (0.67, 0.80)
D+L Subtotal 0.74 (0.64, 0.86)
Former smokers
Health2008 0.72 (0.48, 1.08)
Health2006 0.74 (0.61, 0.90)
1958 BC 0.77 (0.65, 0.92)
Inter99 0.78 (0.68, 0.91)
Dan-Monica10 0.95 (0.69, 1.30)
KORA 1.01 (0.73, 1.39)
The 1936 Cohort 1.37 (0.75, 2.49)
I-V Subtotal (I-squared = 16.6%, p = 0.303) 0.80 (0.74, 0.88)
D+L Subtotal 0.81 (0.73, 0.90)
.2
.4
.6
.8
1
2
3
Odds ratio compared to never smokers

Figure S4

Age- and sex-adjusted association of smoking heaviness with hay fever, asthma and allergic sensitization.

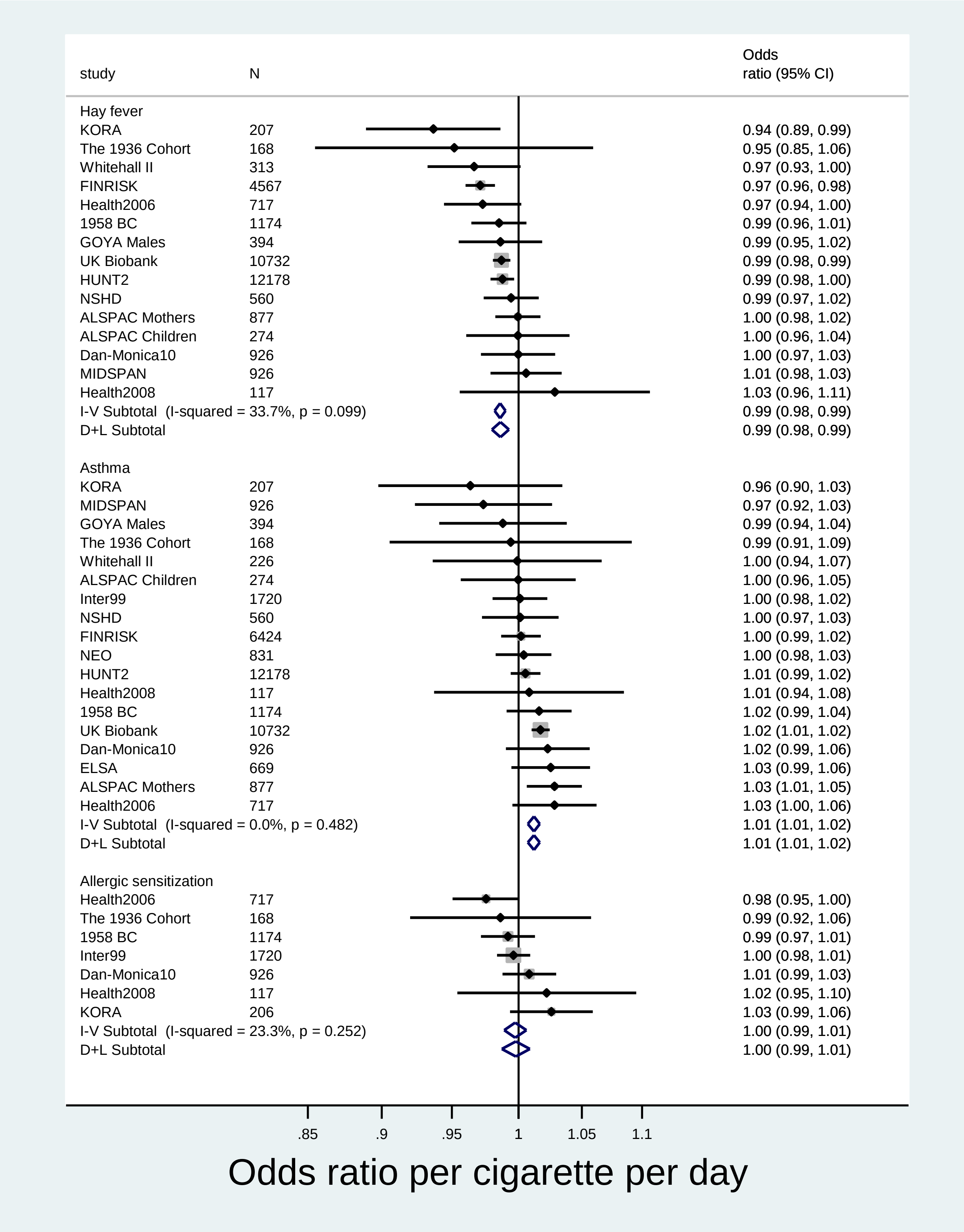

Figure S5-S7

Mendelian randomisation analysis of the age- and sex-adjusted associations of rs1051730/rs16969968 and hay fever, asthma and allergic sensitization.

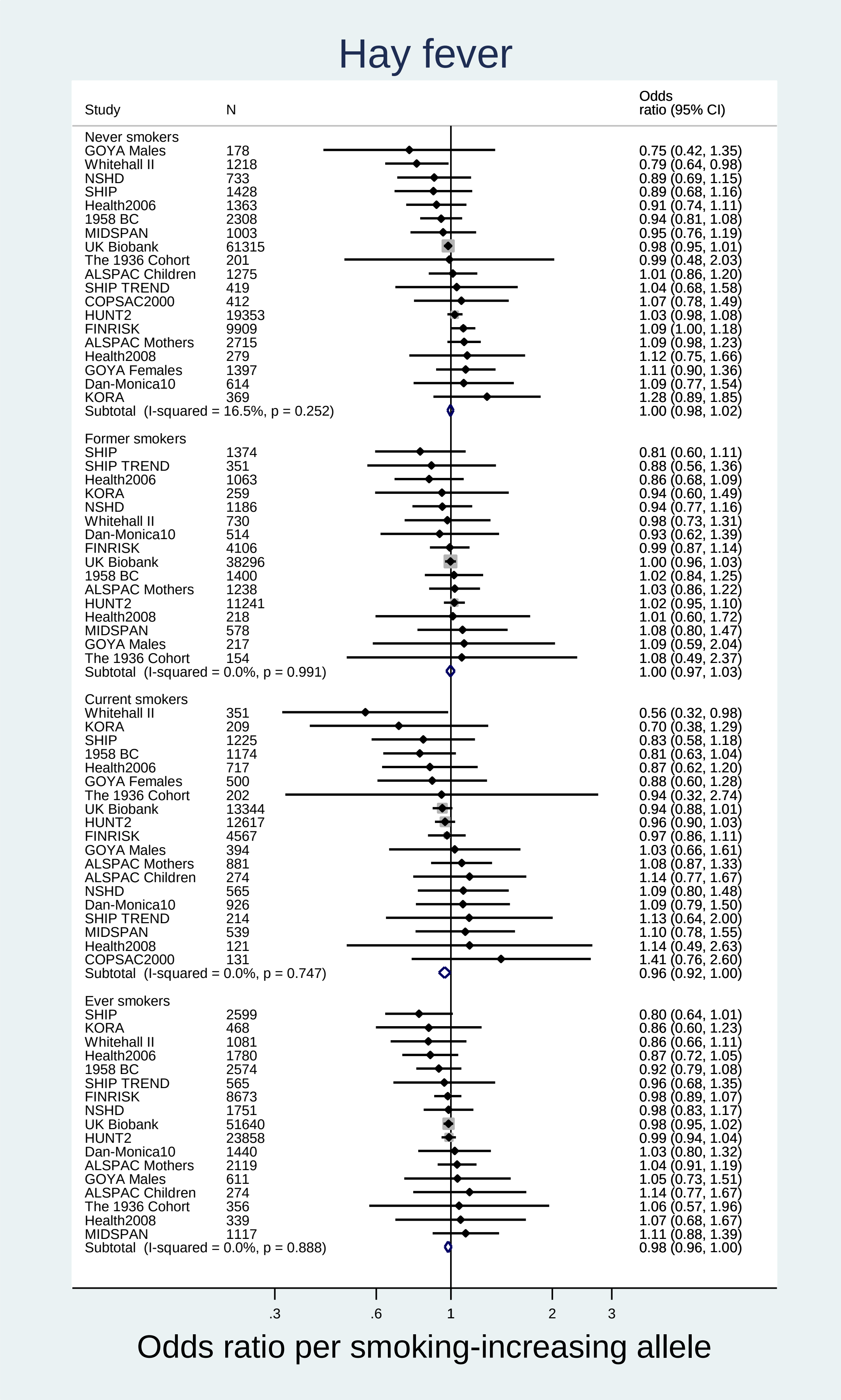
Hay fever
Study
N
Odds ratio (95% CI)
Never smokers
GOYA Males 178 0.75 (0.42, 1.35)
Whitehall II 1218 0.79 (0.64, 0.98)
NSHD 733 0.89 (0.69, 1.15)
SHIP 1428 0.89 (0.68, 1.16)
Health2006 1363 0.91 (0.74, 1.11)
1958 BC 2308 0.94 (0.81, 1.08)
MIDSPAN 1003 0.95 (0.76, 1.19)
UK Biobank 61315 0.98 (0.95, 1.01)
The 1936 Cohort 201 0.99 (0.48, 2.03)
ALSPAC Children 1275 1.01 (0.86, 1.20)
SHIP TREND 419 1.04 (0.68, 1.58)
COPSAC2000 412 1.07 (0.78, 1.49)
HUNT2 19353 1.03 (0.98, 1.08)
FINRISK 9909 1.09 (1.00, 1.18)
ALSPAC Mothers 2715 1.09 (0.98, 1.23)
Health2008 279 1.12 (0.75, 1.66)
GOYA Females 1397 1.11 (0.90, 1.36)
Dan-Monica10 614 1.09 (0.77, 1.54)
KORA 369 1.28 (0.89, 1.85)
Subtotal (I-squared = 16.5%, p = 0.252) 1.00 (0.98, 1.02)
Former smokers
SHIP 1374 0.81 (0.60, 1.11)
SHIP TREND 351 0.88 (0.56, 1.36)
Health2006 1063 0.86 (0.68, 1.09)
KORA 259 0.94 (0.60, 1.49)
NSHD 1186 0.94 (0.77, 1.16)
Whitehall II 730 0.98 (0.73, 1.31)
Dan-Monica10 514 0.93 (0.62, 1.39)
FINRISK 4106 0.99 (0.87, 1.14)
UK Biobank 38296 1.00 (0.96, 1.03)
1958 BC 1400 1.02 (0.84, 1.25)
ALSPAC Mothers 1238 1.03 (0.86, 1.22)
HUNT2 11241 1.02 (0.95, 1.10)
Health2008 218 1.01 (0.60, 1.72)
MIDSPAN 578 1.08 (0.80, 1.47)
GOYA Males 217 1.09 (0.59, 2.04)
The 1936 Cohort 154 1.08 (0.49, 2.37)
Subtotal (I-squared = 0.0%, p = 0.991) 1.00 (0.97, 1.03)
Current smokers
Whitehall II 351 0.56 (0.32, 0.98)
KORA 209 0.70 (0.38, 1.29)
SHIP 1225 0.83 (0.58, 1.18)
1958 BC 1174 0.81 (0.63, 1.04)
Health2006 717 0.87 (0.62, 1.20)
GOYA Females 500 0.88 (0.60, 1.28)
The 1936 Cohort 202 0.94 (0.32, 2.74)
UK Biobank 13344 0.94 (0.88, 1.01)
HUNT2 12617 0.96 (0.90, 1.03)
FINRISK 4567 0.97 (0.86, 1.11)
GOYA Males 394 1.03 (0.66, 1.61)
ALSPAC Mothers 881 1.08 (0.87, 1.33)
ALSPAC Children 274 1.14 (0.77, 1.67)
NSHD 565 1.09 (0.80, 1.48)
Dan-Monica10 926 1.09 (0.79, 1.50)
SHIP TREND 214 1.13 (0.64, 2.00)
MIDSPAN 539 1.10 (0.78, 1.55)
Health2008 121 1.14 (0.49, 2.63)
COPSAC2000 131 1.41 (0.76, 2.60)
Subtotal (I-squared = 0.0%, p = 0.747) 0.96 (0.92, 1.00)
Ever smokers
SHIP 2599 0.80 (0.64, 1.01)
KORA 468 0.86 (0.60, 1.23)
Whitehall II 1081 0.86 (0.66, 1.11)
Health2006 1780 0.87 (0.72, 1.05)
1958 BC 2574 0.92 (0.79, 1.08)
SHIP TREND 565 0.96 (0.68, 1.35)
FINRISK 8673 0.98 (0.89, 1.07)
NSHD 1751 0.98 (0.83, 1.17)
UK Biobank 51640 0.98 (0.95, 1.02)
HUNT2 23858 0.99 (0.94, 1.04)
Dan-Monica10 1440 1.03 (0.80, 1.32)
ALSPAC Mothers 2119 1.04 (0.91, 1.19)
GOYA Males 611 1.05 (0.73, 1.51)
ALSPAC Children 274 1.14 (0.77, 1.67)
The 1936 Cohort 356 1.06 (0.57, 1.96)
Health2008 339 1.07 (0.68, 1.67)
MIDSPAN 1117 1.11 (0.88, 1.39)
Subtotal (I-squared = 0.0%, p = 0.888) 0.98 (0.96, 1.00)
.3
.6
1
2
3
Odds ratio per smoking-increasing allele

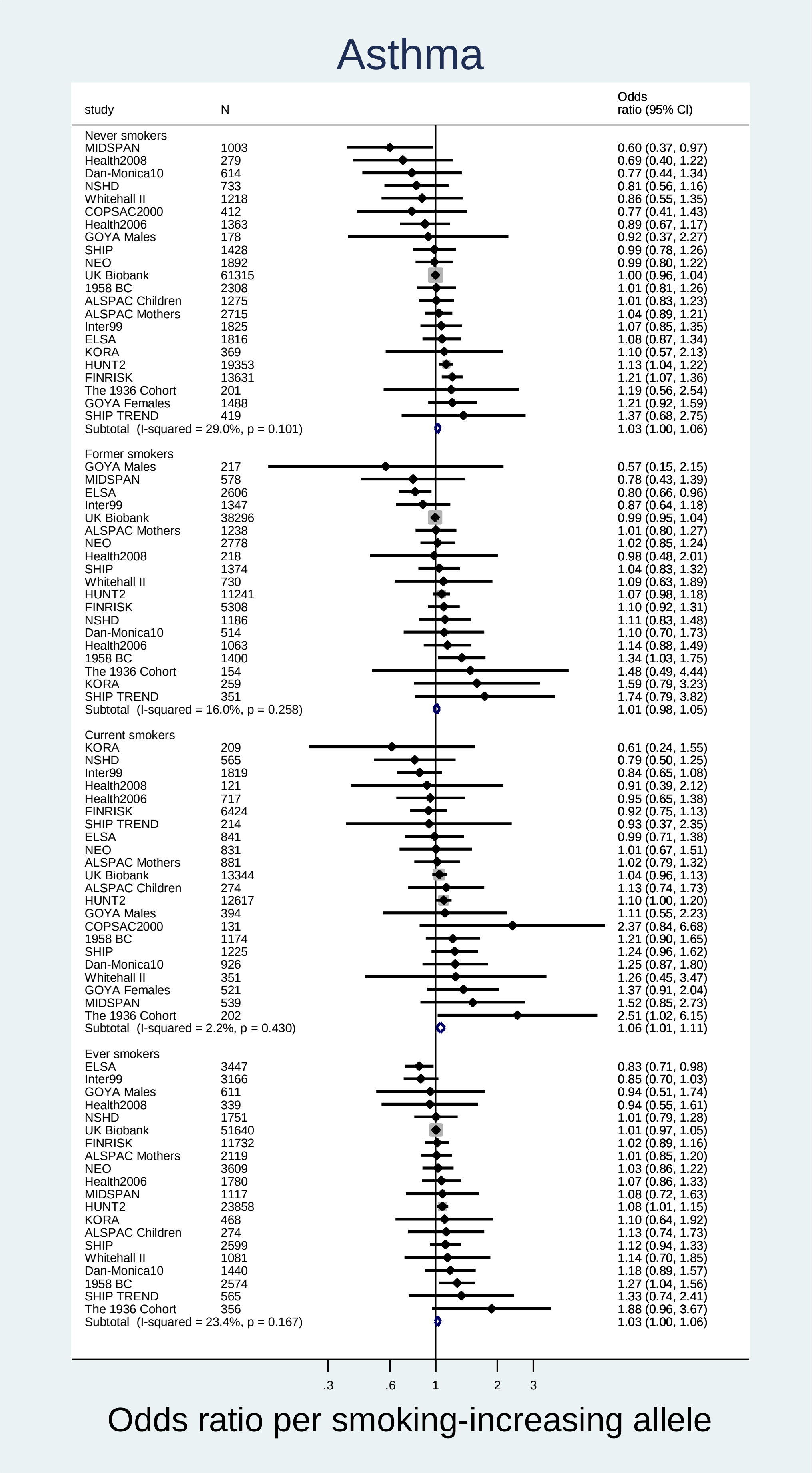
Asthma
study N Odds ratio (95% CI)
Never smokers
MIDSPAN 1003 0.60 (0.37, 0.97)
Health2008 279 0.69 (0.40, 1.22)
Dan-Monica10 614 0.77 (0.44, 1.34)
NSHD 733 0.81 (0.56, 1.16)
Whitehall II 1218 0.86 (0.55, 1.35)
COPSAC2000 412 0.77 (0.41, 1.43)
Health2006 1363 0.89 (0.67, 1.17)
GOYA Males 178 0.92 (0.37, 2.27)
SHIP 1428 0.99 (0.78, 1.26)
NEO 1892 0.99 (0.80, 1.22)
UK Biobank 61315 1.00 (0.96, 1.04)
1958 BC 2308 1.01 (0.81, 1.26)
ALSPAC Children 1275 1.01 (0.83, 1.23)
ALSPAC Mothers 2715 1.04 (0.89, 1.21)
Inter99 1825 1.07 (0.85, 1.35)
ELSA 1816 1.08 (0.87, 1.34)
KORA 369 1.10 (0.57, 2.13)
HUNT2 19353 1.13 (1.04, 1.22)
FINRISK 13631 1.21 (1.07, 1.36)
The 1936 Cohort 201 1.19 (0.56, 2.54)
GOYA Females 1488 1.21 (0.92, 1.59)
SHIP TREND 419 1.37 (0.68, 2.75)
Subtotal (I-squared = 29.0%, p = 0.101) 1.03 (1.00, 1.06)
Former smokers
GOYA Males 217 0.57 (0.15, 2.15)
MIDSPAN 578 0.78 (0.43, 1.39)
ELSA 2606 0.80 (0.66, 0.96)
Inter99 1347 0.87 (0.64, 1.18)
UK Biobank 38296 0.99 (0.95, 1.04)
ALSPAC Mothers 1238 1.01 (0.80, 1.27)
NEO 2778 1.02 (0.85, 1.24)
Health2008 218 0.98 (0.48, 2.01)
SHIP 1374 1.04 (0.83, 1.32)
Whitehall II 730 1.09 (0.63, 1.89)
HUNT2 11241 1.07 (0.98, 1.18)
FINRISK 5308 1.10 (0.92, 1.31)
NSHD 1186 1.11 (0.83, 1.48)
Dan-Monica10 514 1.10 (0.70, 1.73)
Health2006 1063 1.14 (0.88, 1.49)
1958 BC 1400 1.34 (1.03, 1.75)
The 1936 Cohort 154 1.48 (0.49, 4.44)
KORA 259 1.59 (0.79, 3.23)
SHIP TREND 351 1.74 (0.79, 3.82)
Subtotal (I-squared = 16.0%, p = 0.258) 1.01 (0.98, 1.05)
Current smokers
KORA 209 0.61 (0.24, 1.55)
NSHD 565 0.79 (0.50, 1.25)
Inter99 1819 0.84 (0.65, 1.08)
Health2008 121 0.91 (0.39, 2.12)
Health2006 717 0.95 (0.65, 1.38)
FINRISK 6424 0.92 (0.75, 1.13)
SHIP TREND 214 0.93 (0.37, 2.35)
ELSA 841 0.99 (0.71, 1.38)
NEO 831 1.01 (0.67, 1.51)
ALSPAC Mothers 881 1.02 (0.79, 1.32)
UK Biobank 13344 1.04 (0.96, 1.13)
ALSPAC Children 274 1.13 (0.74, 1.73)
HUNT2 12617 1.10 (1.00, 1.20)
GOYA Males 394 1.11 (0.55, 2.23)
COPSAC2000 131 2.37 (0.84, 6.68)
1958 BC 1174 1.21 (0.90, 1.65)
SHIP 1225 1.24 (0.96, 1.62)
Dan-Monica10 926 1.25 (0.87, 1.80)
Whitehall II 351 1.26 (0.45, 3.47)
GOYA Females 521 1.37 (0.91, 2.04)
MIDSPAN 539 1.52 (0.85, 2.73)
The 1936 Cohort 202 2.51 (1.02, 6.15)
Subtotal (I-squared = 2.2%, p = 0.430) 1.06 (1.01, 1.11)
Ever smokers
ELSA 3447 0.83 (0.71, 0.98)
Inter99 3166 0.85 (0.70, 1.03)
GOYA Males 611 0.94 (0.51, 1.74)
Health2008 339 0.94 (0.55, 1.61)
NSHD 1751 1.01 (0.79, 1.28)
UK Biobank 51640 1.01 (0.97, 1.05)
FINRISK 11732 1.02 (0.89, 1.16)
ALSPAC Mothers 2119 1.01 (0.85, 1.20)
NEO 3609 1.03 (0.86, 1.22)
Health2006 1780 1.07 (0.86, 1.33)
MIDSPAN 1117 1.08 (0.72, 1.63)
HUNT2 23858 1.08 (1.01, 1.15)
KORA 468 1.10 (0.64, 1.92)
ALSPAC Children 274 1.13 (0.74, 1.73)
SHIP 2599 1.12 (0.94, 1.33)
Whitehall II 1081 1.14 (0.70, 1.85)
Dan-Monica10 1440 1.18 (0.89, 1.57)
1958 BC 2574 1.27 (1.04, 1.56)
SHIP TREND 565 1.33 (0.74, 2.41)
The 1936 Cohort 356 1.88 (0.96, 3.67)
Subtotal (I-squared = 23.4%, p = 0.167) 1.03 (1.00, 1.06)
.3 .6 1 2 3
Odds ratio per smoking-increasing allele

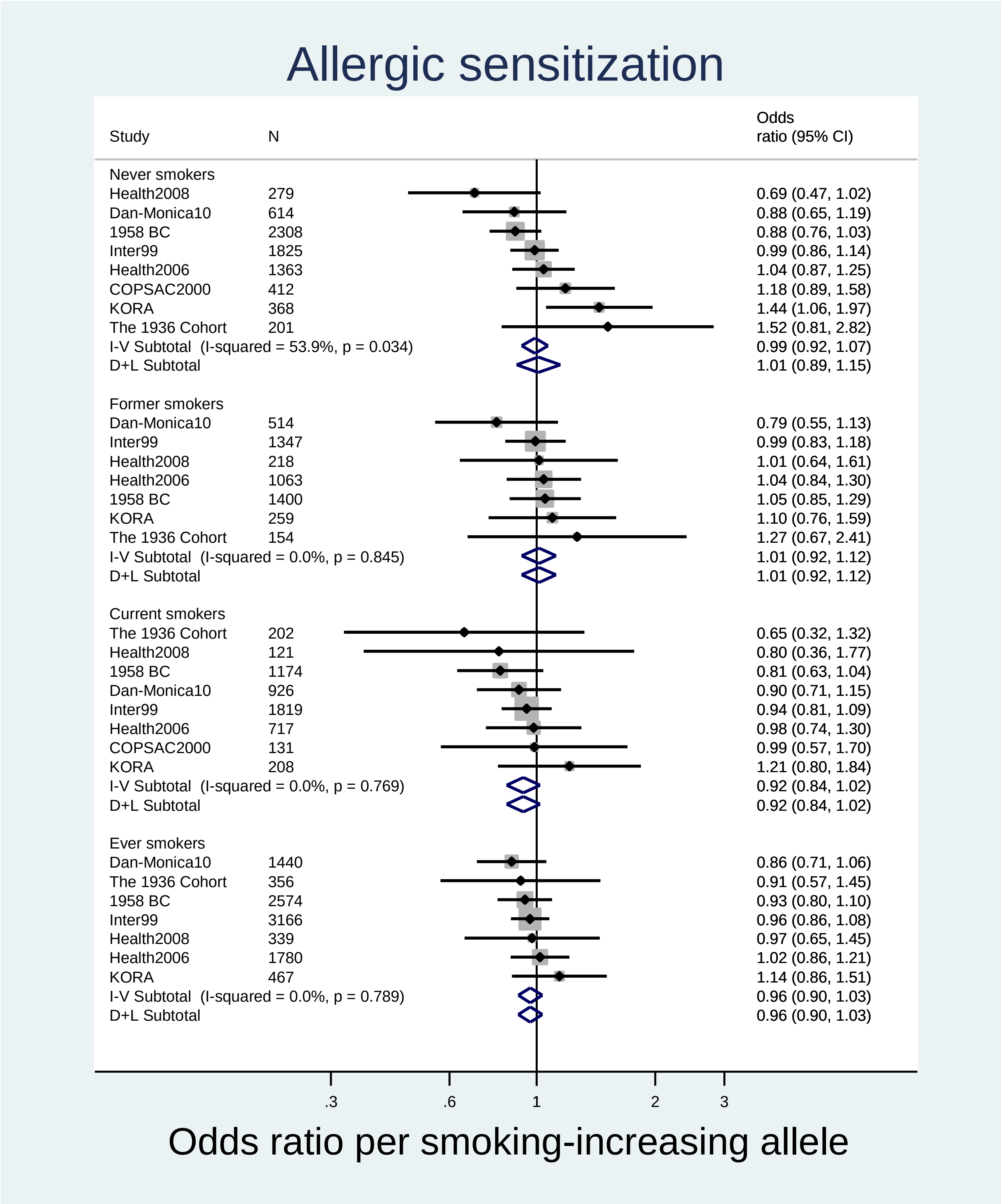
Allergic sensitization
Study
N
Odds ratio (95% CI)
Never smokers
Health2008 279 0.69 (0.47, 1.02)
Dan-Monica10 614 0.88 (0.65, 1.19)
1958 BC 2308 0.88 (0.76, 1.03)
Inter99 1825 0.99 (0.86, 1.14)
Health2006 1363 1.04 (0.87, 1.25)
COPSAC2000 412 1.18 (0.89, 1.58)
KORA 368 1.44 (1.06, 1.97)
The 1936 Cohort 201 1.52 (0.81, 2.82)
I-V Subtotal (I-squared = 53.9%, p = 0.034) 0.99 (0.92, 1.07)
D+L Subtotal 1.01 (0.89, 1.15)
Former smokers
Dan-Monica10 514 0.79 (0.55, 1.13)
Inter99 1347 0.99 (0.83, 1.18)
Health2008 218 1.01 (0.64, 1.61)
Health2006 1063 1.04 (0.84, 1.30)
1958 BC 1400 1.05 (0.85, 1.29)
KORA 259 1.10 (0.76, 1.59)
The 1936 Cohort 154 1.27 (0.67, 2.41)
I-V Subtotal (I-squared = 0.0%, p = 0.845) 1.01 (0.92, 1.12)
D+L Subtotal 1.01 (0.92, 1.12)
Current smokers
The 1936 Cohort 202 0.65 (0.32, 1.32)
Health2008 121 0.80 (0.36, 1.77)
1958 BC 1174 0.81 (0.63, 1.04)
Dan-Monica10 926 0.90 (0.71, 1.15)
Inter99 1819 0.94 (0.81, 1.09)
Health2006 717 0.98 (0.74, 1.30)
COPSAC2000 131 0.99 (0.57, 1.70)
KORA 208 1.21 (0.80, 1.84)
I-V Subtotal (I-squared = 0.0%, p = 0.769) 0.92 (0.84, 1.02)
D+L Subtotal 0.92 (0.84, 1.02)
Ever smokers
Dan-Monica10 1440 0.86 (0.71, 1.06)
The 1936 Cohort 356 0.91 (0.57, 1.45)
1958 BC 2574 0.93 (0.80, 1.10)
Inter99 3166 0.96 (0.86, 1.08)
Health2008 339 0.97 (0.65, 1.45)
Health2006 1780 1.02 (0.86, 1.21)
KORA 467 1.14 (0.86, 1.51)
I-V Subtotal (I-squared = 0.0%, p = 0.789) 0.96 (0.90, 1.03)
D+L Subtotal 0.96 (0.90, 1.03)
.3
.6
1
2
3
Odds ratio per smoking-increasing allele

Figure S8

Age- and sex- adjusted association of genotype and smoking heaviness.

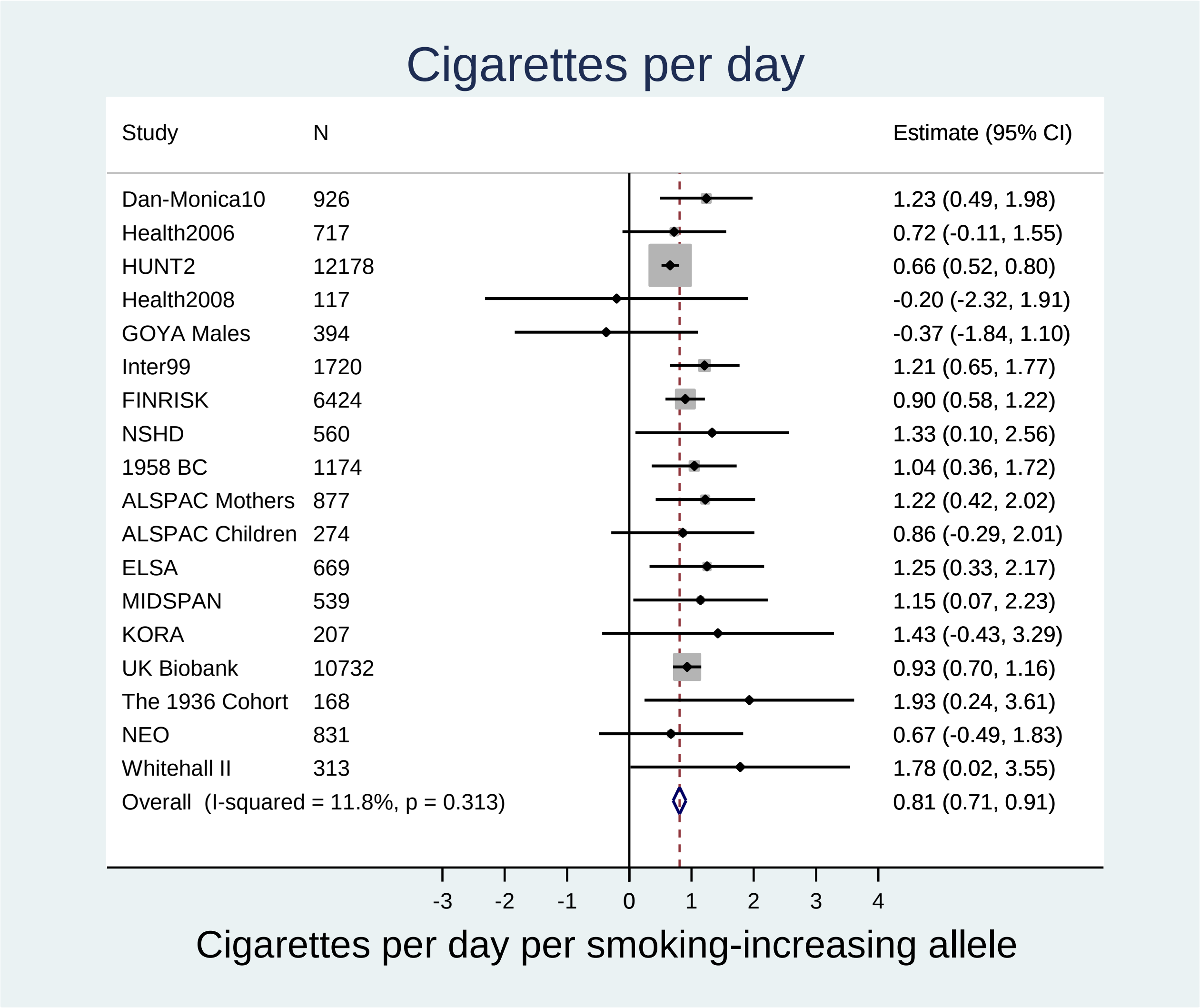

Crude analyses

Figure S9-S11

Crude association of smoking status with hay fever, asthma and allergic sensitization.

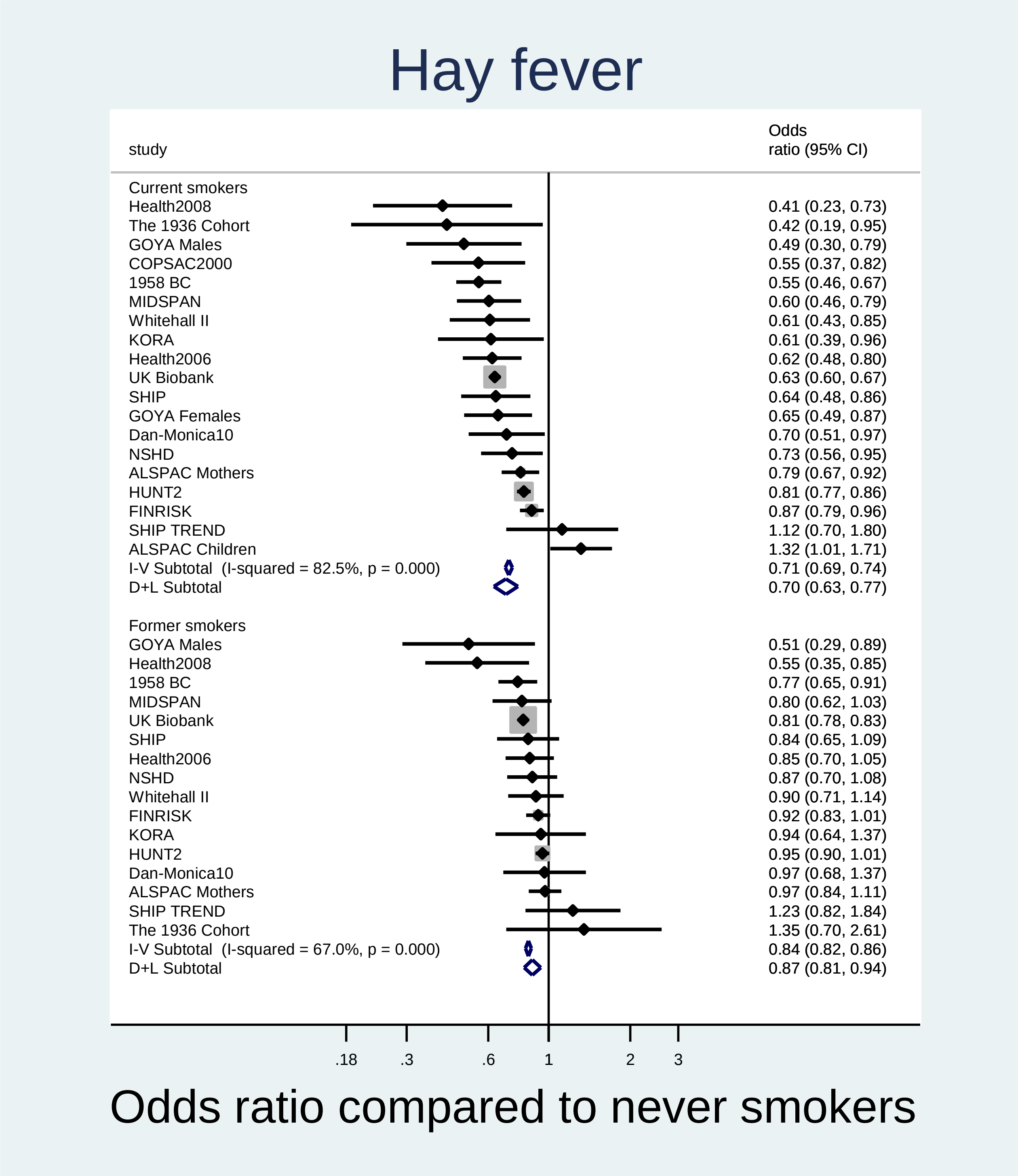

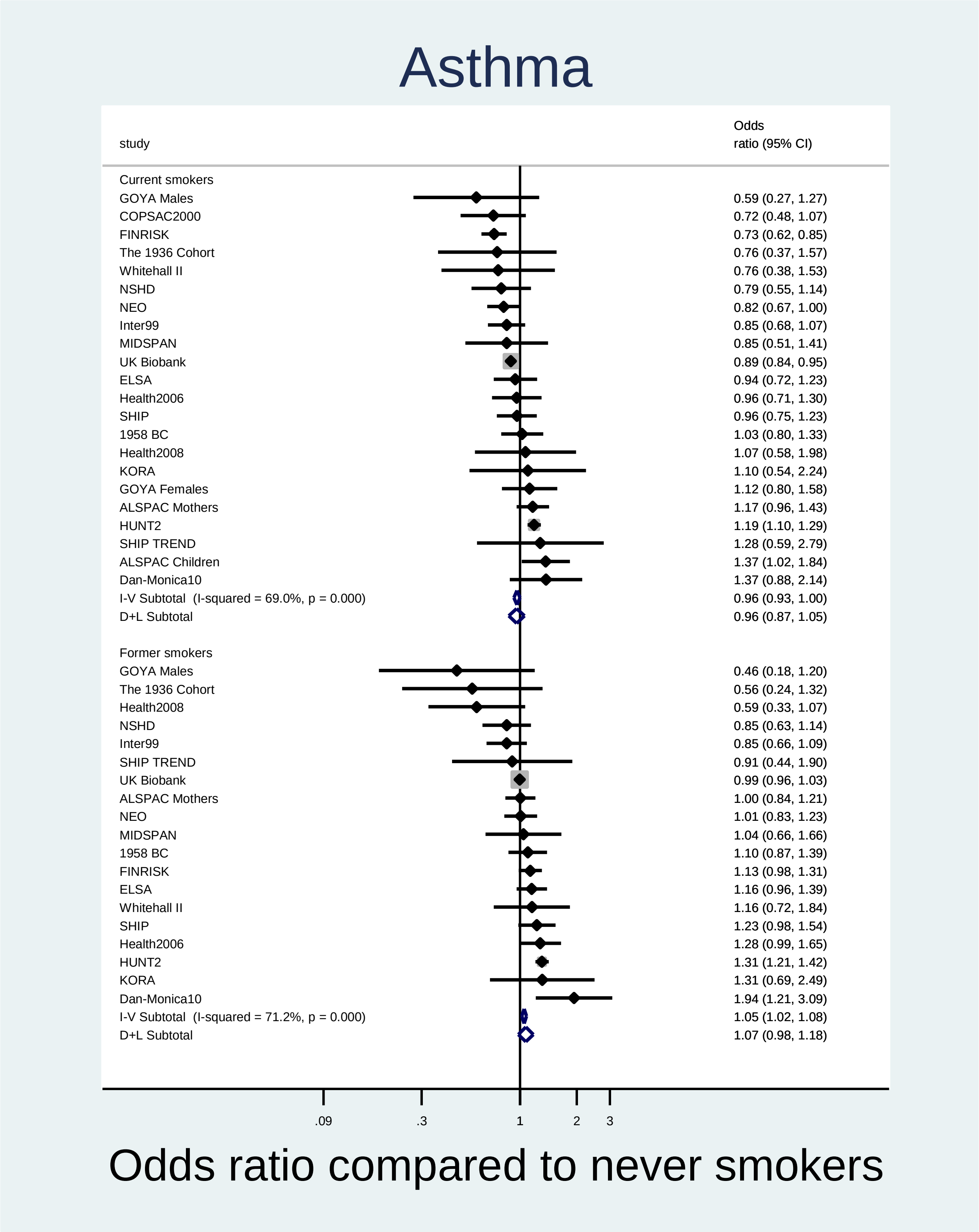
Asthma
study
Odds ratio (95% CI)
Current smokers
GOYA Males 0.59 (0.27, 1.27)
COPSAC2000 0.72 (0.48, 1.07)
FINRISK 0.73 (0.62, 0.85)
The 1936 Cohort 0.76 (0.37, 1.57)
Whitehall II 0.76 (0.38, 1.53)
NSHD 0.79 (0.55, 1.14)
NEO 0.82 (0.67, 1.00)
Inter99 0.85 (0.68, 1.07)
MIDSPAN 0.85 (0.51, 1.41)
UK Biobank 0.89 (0.84, 0.95)
ELSA 0.94 (0.72, 1.23)
Health2006 0.96 (0.71, 1.30)
SHIP 0.96 (0.75, 1.23)
1958 BC 1.03 (0.80, 1.33)
Health2008 1.07 (0.58, 1.98)
KORA 1.10 (0.54, 2.24)
GOYA Females 1.12 (0.80, 1.58)
ALSPAC Mothers 1.17 (0.96, 1.43)
HUNT2 1.19 (1.10, 1.29)
SHIP TREND 1.28 (0.59, 2.79)
ALSPAC Children 1.37 (1.02, 1.84)
Dan-Monica10 1.37 (0.88, 2.14)
I-V Subtotal (I-squared = 69.0%, p = 0.000) 0.96 (0.93, 1.00)
D+L Subtotal 0.96 (0.87, 1.05)
Former smokers
GOYA Males 0.46 (0.18, 1.20)
The 1936 Cohort 0.56 (0.24, 1.32)
Health2008 0.59 (0.33, 1.07)
NSHD 0.85 (0.63, 1.14)
Inter99 0.85 (0.66, 1.09)
SHIP TREND 0.91 (0.44, 1.90)
UK Biobank 0.99 (0.96, 1.03)
ALSPAC Mothers 1.00 (0.84, 1.21)
NEO 1.01 (0.83, 1.23)
MIDSPAN 1.04 (0.66, 1.66)
1958 BC 1.10 (0.87, 1.39)
FINRISK 1.13 (0.98, 1.31)
ELSA 1.16 (0.96, 1.39)
Whitehall II 1.16 (0.72, 1.84)
SHIP 1.23 (0.98, 1.54)
Health2006 1.28 (0.99, 1.65)
HUNT2 1.31 (1.21, 1.42)
KORA 1.31 (0.69, 2.49)
Dan-Monica10 1.94 (1.21, 3.09)
I-V Subtotal (I-squared = 71.2%, p = 0.000) 1.05 (1.02, 1.08)
D+L Subtotal 1.07 (0.98, 1.18)
.09
.3
1
2
3
Odds ratio compared to never smokers

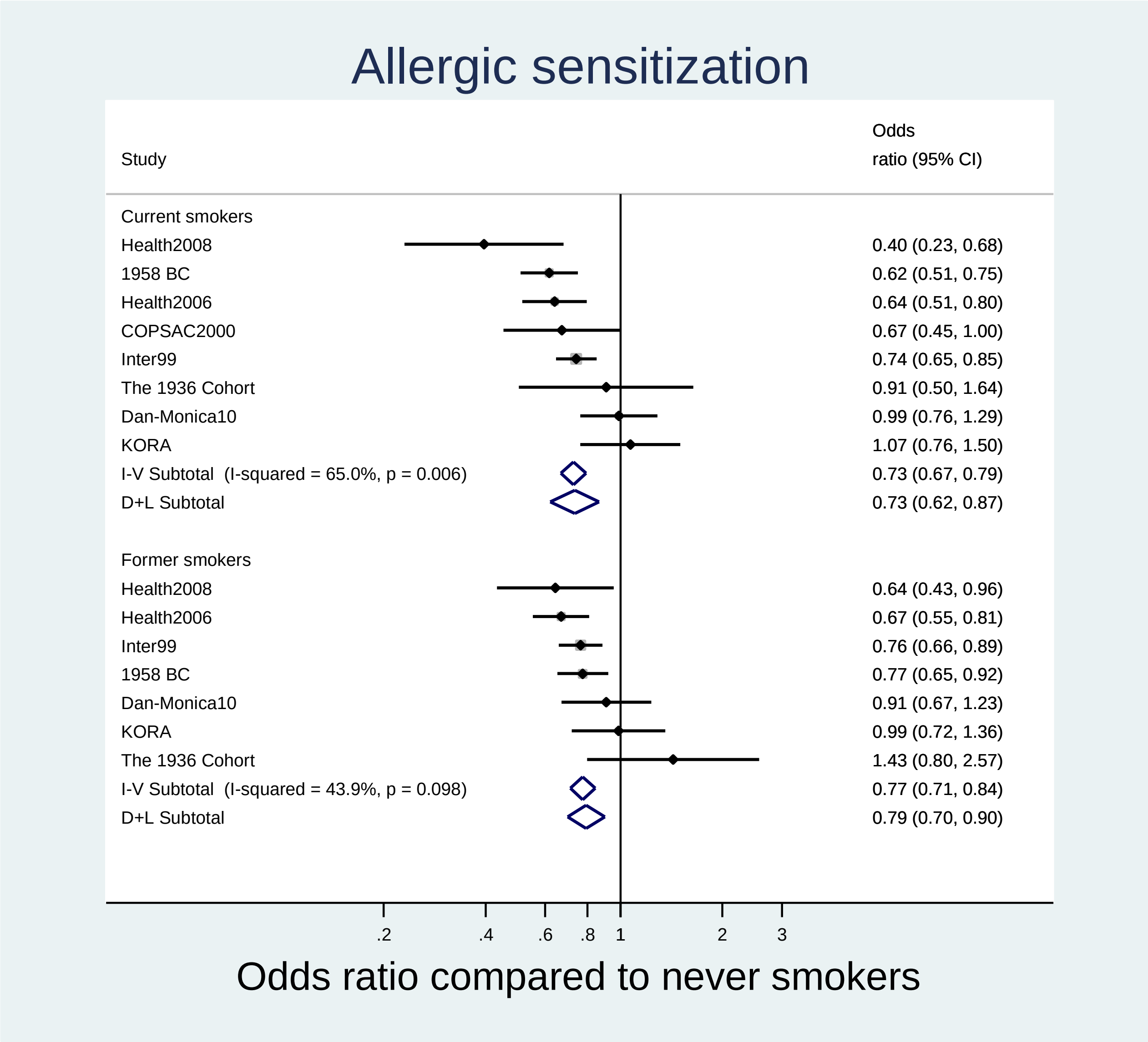

Allergic sensitization
Study
Odds
ratio (95% CI)
Current smokers
Health2008 0.40 (0.23, 0.68)
1958 BC 0.62 (0.51, 0.75)
Health2006 0.64 (0.51, 0.80)
COPSAC2000 0.67 (0.45, 1.00)
Inter99 0.74 (0.65, 0.85)
The 1936 Cohort 0.91 (0.50, 1.64)
Dan-Monica10 0.99 (0.76, 1.29)
KORA 1.07 (0.76, 1.50)
I-V Subtotal (I-squared = 65.0%, p = 0.006) 0.73 (0.67, 0.79)
D+L Subtotal 0.73 (0.62, 0.87)
Former smokers
Health2008 0.64 (0.43, 0.96)
Health2006 0.67 (0.55, 0.81)
Inter99 0.76 (0.66, 0.89)
1958 BC 0.77 (0.65, 0.92)
Dan-Monica10 0.91 (0.67, 1.23)
KORA 0.99 (0.72, 1.36)
The 1936 Cohort 1.43 (0.80, 2.57)
I-V Subtotal (I-squared = 43.9%, p = 0.098) 0.77 (0.71, 0.84)
D+L Subtotal 0.79 (0.70, 0.90)
.2
.4
.6
.8
1
2
3
Odds ratio compared to never smokers

Figure S12

Crude association of smoking heaviness with hay fever, asthma and allergic sensitization

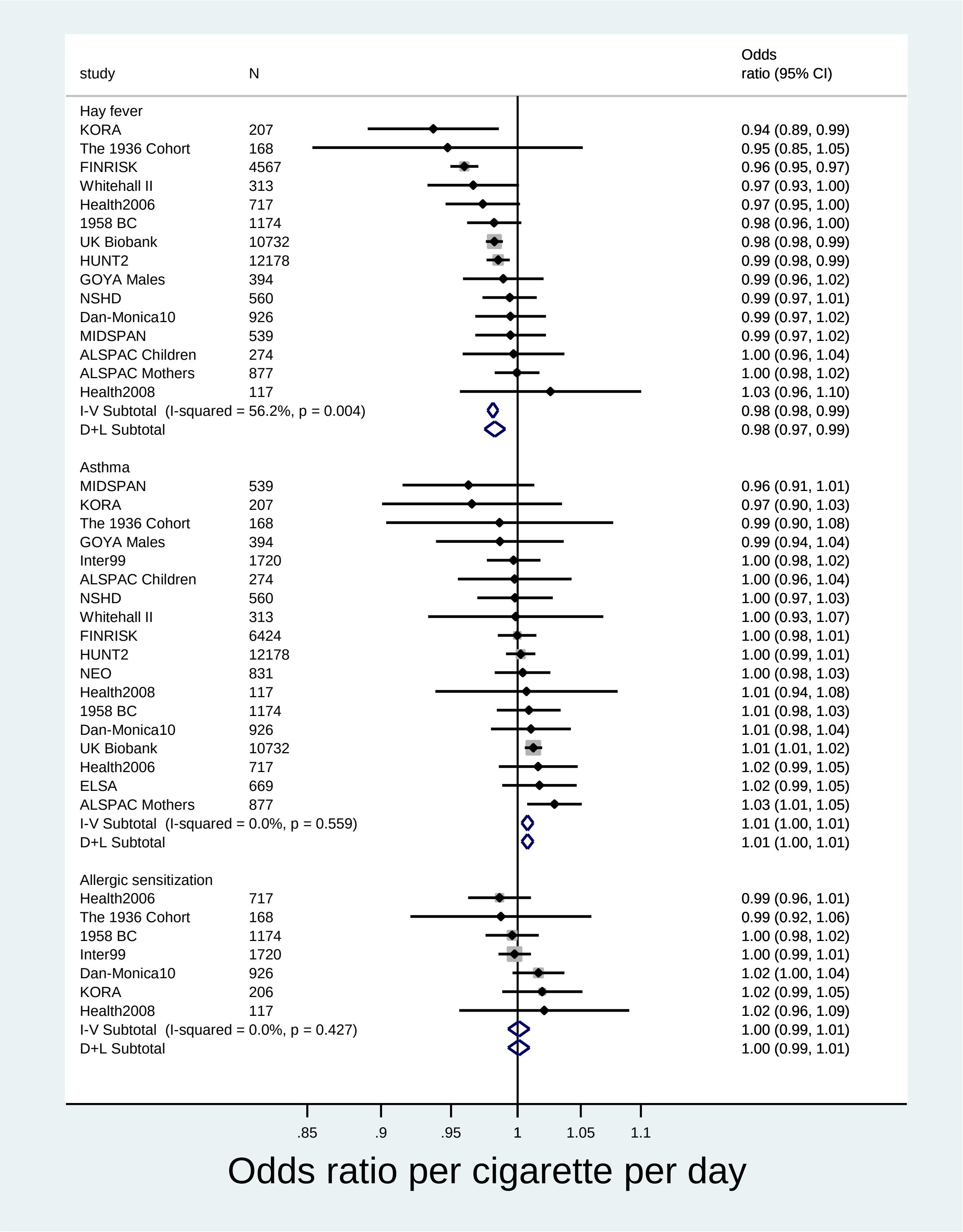

Figure S13-S15

Mendelian randomization analysis of the crude associations of rs1051730/rs16969968 and hay fever (N=208,365), asthma (N=231,013) and allergic sensitization (N=17,623).

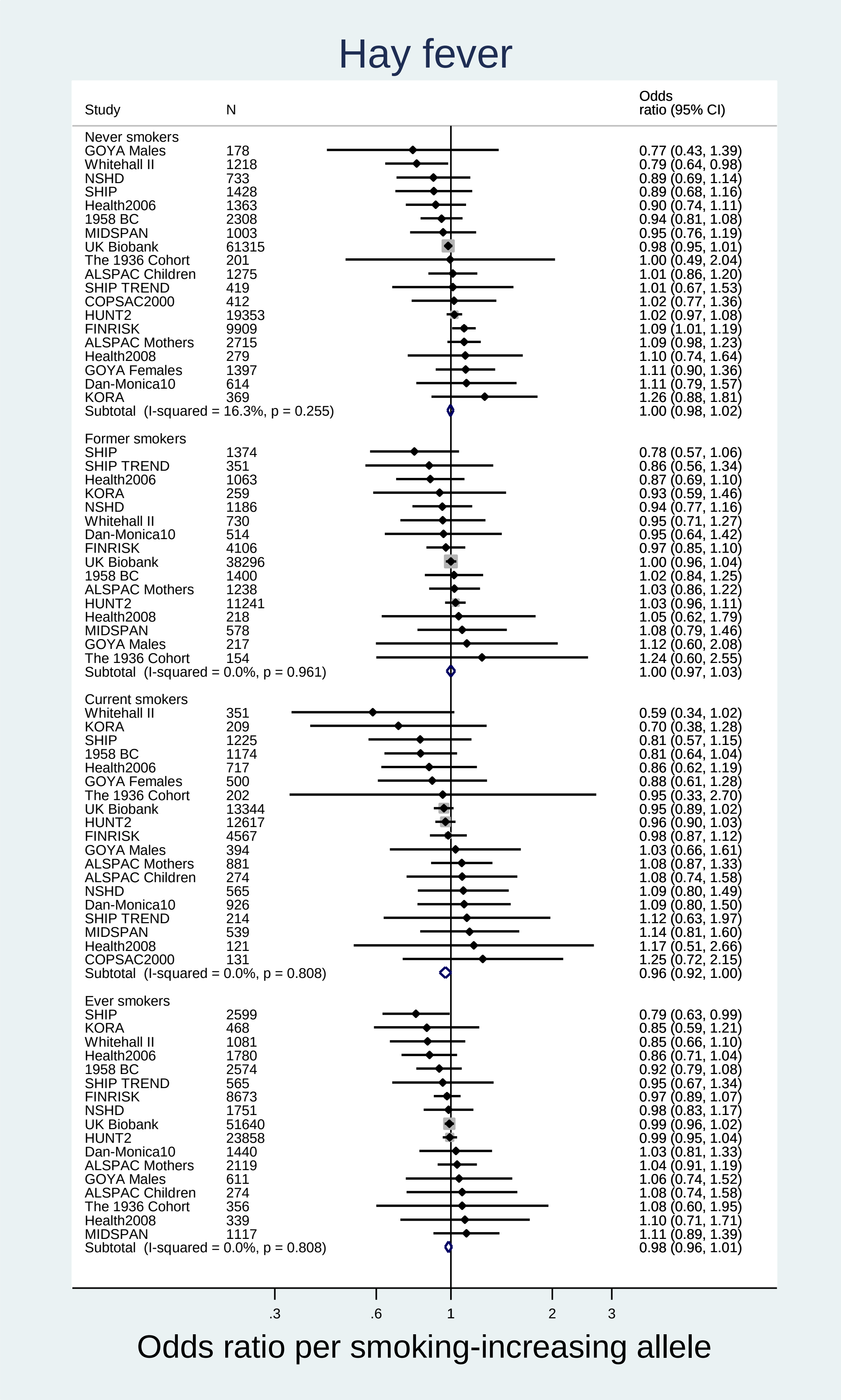
Hay fever
Study
N
Odds
ratio (95% CI)
Never smokers
GOYA Males 178 0.77 (0.43, 1.39)
Whitehall II 1218 0.79 (0.64, 0.98)
NSHD 733 0.89 (0.69, 1.14)
SHIP 1428 0.89 (0.68, 1.16)
Health2006 1363 0.90 (0.74, 1.11)
1958 BC 2308 0.94 (0.81, 1.08)
MIDSPAN 1003 0.95 (0.76, 1.19)
UK Biobank 61315 0.98 (0.95, 1.01)
The 1936 Cohort 201 1.00 (0.49, 2.04)
ALSPAC Children 1275 1.01 (0.86, 1.20)
SHIP TREND 419 1.01 (0.67, 1.53)
COPSAC2000 412 1.02 (0.77, 1.36)
HUNT2 19353 1.02 (0.97, 1.08)
FINRISK 9909 1.09 (1.01, 1.19)
ALSPAC Mothers 2715 1.09 (0.98, 1.23)
Health2008 279 1.10 (0.74, 1.64)
GOYA Females 1397 1.11 (0.90, 1.36)
Dan-Monica10 614 1.11 (0.79, 1.57)
KORA 369 1.26 (0.88, 1.81)
Subtotal (I-squared = 16.3%, p = 0.255) 1.00 (0.98, 1.02)
Former smokers
SHIP 1374 0.78 (0.57, 1.06)
SHIP TREND 351 0.86 (0.56, 1.34)
Health2006 1063 0.87 (0.69, 1.10)
KORA 259 0.93 (0.59, 1.46)
NSHD 1186 0.94 (0.77, 1.16)
Whitehall II 730 0.95 (0.71, 1.27)
Dan-Monica10 514 0.95 (0.64, 1.42)
FINRISK 4106 0.97 (0.85, 1.10)
UK Biobank 38296 1.00 (0.96, 1.04)
1958 BC 1400 1.02 (0.84, 1.25)
ALSPAC Mothers 1238 1.03 (0.86, 1.22)
HUNT2 11241 1.03 (0.96, 1.11)
Health2008 218 1.05 (0.62, 1.79)
MIDSPAN 578 1.08 (0.79, 1.46)
GOYA Males 217 1.12 (0.60, 2.08)
The 1936 Cohort 154 1.24 (0.60, 2.55)
Subtotal (I-squared = 0.0%, p = 0.961) 1.00 (0.97, 1.03)
Current smokers
Whitehall II 351 0.59 (0.34, 1.02)
KORA 209 0.70 (0.38, 1.28)
SHIP 1225 0.81 (0.57, 1.15)
1958 BC 1174 0.81 (0.64, 1.04)
Health2006 717 0.86 (0.62, 1.19)
GOYA Females 500 0.88 (0.61, 1.28)
The 1936 Cohort 202 0.95 (0.33, 2.70)
UK Biobank 13344 0.95 (0.89, 1.02)
HUNT2 12617 0.96 (0.90, 1.03)
FINRISK 4567 0.98 (0.87, 1.12)
GOYA Males 394 1.03 (0.66, 1.61)
ALSPAC Mothers 881 1.08 (0.87, 1.33)
ALSPAC Children 274 1.08 (0.74, 1.58)
NSHD 565 1.09 (0.80, 1.49)
Dan-Monica10 926 1.09 (0.80, 1.50)
SHIP TREND 214 1.12 (0.63, 1.97)
MIDSPAN 539 1.14 (0.81, 1.60)
Health2008 121 1.17 (0.51, 2.66)
COPSAC2000 131 1.25 (0.72, 2.15)
Subtotal (I-squared = 0.0%, p = 0.808) 0.96 (0.92, 1.00)
Ever smokers
SHIP 2599 0.79 (0.63, 0.99)
KORA 468 0.85 (0.59, 1.21)
Whitehall II 1081 0.85 (0.66, 1.10)
Health2006 1780 0.86 (0.71, 1.04)
1958 BC 2574 0.92 (0.79, 1.08)
SHIP TREND 565 0.95 (0.67, 1.34)
FINRISK 8673 0.97 (0.89, 1.07)
NSHD 1751 0.98 (0.83, 1.17)
UK Biobank 51640 0.99 (0.96, 1.02)
HUNT2 23858 0.99 (0.95, 1.04)
Dan-Monica10 1440 1.03 (0.81, 1.33)
ALSPAC Mothers 2119 1.04 (0.91, 1.19)
GOYA Males 611 1.06 (0.74, 1.52)
ALSPAC Children 274 1.08 (0.74, 1.58)
The 1936 Cohort 356 1.08 (0.60, 1.95)
Health2008 339 1.10 (0.71, 1.71)
MIDSPAN 1117 1.11 (0.89, 1.39)
Subtotal (I-squared = 0.0%, p = 0.808) 0.98 (0.96, 1.01)
.3
.6
1
2
3
Odds ratio per smoking-increasing allele

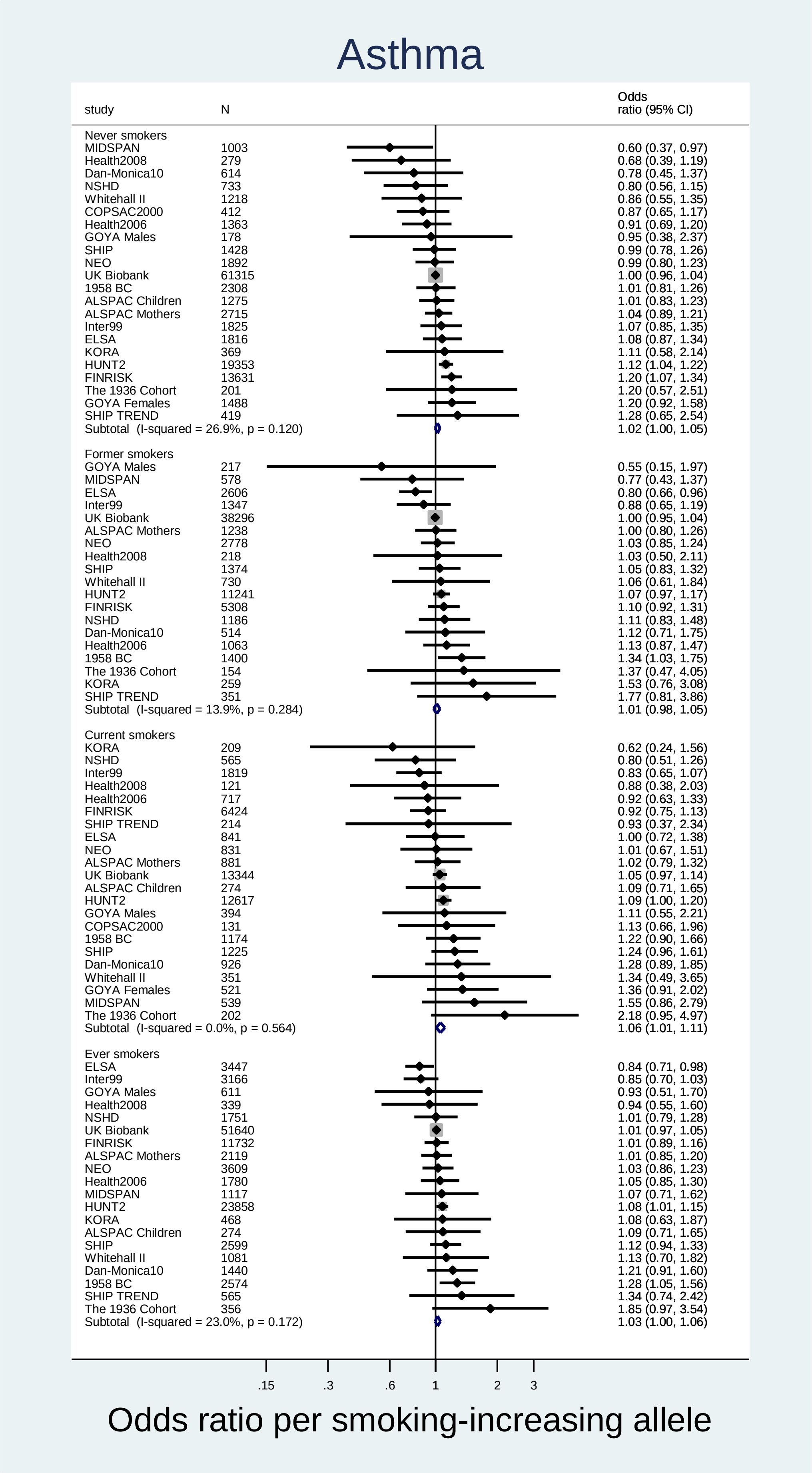
Asthma
study
N
Odds ratio (95% CI)
Never smokers
MIDSPAN 1003 0.60 (0.37, 0.97)
Health2008 279 0.68 (0.39, 1.19)
Dan-Monica10 614 0.78 (0.45, 1.37)
NSHD 733 0.80 (0.56, 1.15)
Whitehall II 1218 0.86 (0.55, 1.35)
COPSAC2000 412 0.87 (0.65, 1.17)
Health2006 1363 0.91 (0.69, 1.20)
GOYA Males 178 0.95 (0.38, 2.37)
SHIP 1428 0.99 (0.78, 1.26)
NEO 1892 0.99 (0.80, 1.23)
UK Biobank 61315 1.00 (0.96, 1.04)
1958 BC 2308 1.01 (0.81, 1.26)
ALSPAC Children 1275 1.01 (0.83, 1.23)
ALSPAC Mothers 2715 1.04 (0.89, 1.21)
Inter99 1825 1.07 (0.85, 1.35)
ELSA 1816 1.08 (0.87, 1.34)
KORA 369 1.11 (0.58, 2.14)
HUNT2 19353 1.12 (1.04, 1.22)
FINRISK 13631 1.20 (1.07, 1.34)
The 1936 Cohort 201 1.20 (0.57, 2.51)
GOYA Females 1488 1.20 (0.92, 1.58)
SHIP TREND 419 1.28 (0.65, 2.54)
Subtotal (I-squared = 26.9%, p = 0.120) 1.02 (1.00, 1.05)
Former smokers
GOYA Males 217 0.55 (0.15, 1.97)
MIDSPAN 578 0.77 (0.43, 1.37)
ELSA 2606 0.80 (0.66, 0.96)
Inter99 1347 0.88 (0.65, 1.19)
UK Biobank 38296 1.00 (0.95, 1.04)
ALSPAC Mothers 1238 1.00 (0.80, 1.26)
NEO 2778 1.03 (0.85, 1.24)
Health2008 218 1.03 (0.50, 2.11)
SHIP 1374 1.05 (0.83, 1.32)
Whitehall II 730 1.06 (0.61, 1.84)
HUNT2 11241 1.07 (0.97, 1.17)
FINRISK 5308 1.10 (0.92, 1.31)
NSHD 1186 1.11 (0.83, 1.48)
Dan-Monica10 514 1.12 (0.71, 1.75)
Health2006 1063 1.13 (0.87, 1.47)
1958 BC 1400 1.34 (1.03, 1.75)
The 1936 Cohort 154 1.37 (0.47, 4.05)
KORA 259 1.53 (0.76, 3.08)
SHIP TREND 351 1.77 (0.81, 3.86)
Subtotal (I-squared = 13.9%, p = 0.284) 1.01 (0.98, 1.05)
Current smokers
KORA 209 0.62 (0.24, 1.56)
NSHD 565 0.80 (0.51, 1.26)
Inter99 1819 0.83 (0.65, 1.07)
Health2008 121 0.88 (0.38, 2.03)
Health2006 717 0.92 (0.63, 1.33)
FINRISK 6424 0.92 (0.75, 1.13)
SHIP TREND 214 0.93 (0.37, 2.34)
ELSA 841 1.00 (0.72, 1.38)
NEO 831 1.01 (0.67, 1.51)
ALSPAC Mothers 881 1.02 (0.79, 1.32)
UK Biobank 13344 1.05 (0.97, 1.14)
ALSPAC Children 274 1.09 (0.71, 1.65)
HUNT2 12617 1.09 (1.00, 1.20)
GOYA Males 394 1.11 (0.55, 2.21)
COPSAC2000 131 1.13 (0.66, 1.96)
1958 BC 1174 1.22 (0.90, 1.66)
SHIP 1225 1.24 (0.96, 1.61)
Dan-Monica10 926 1.28 (0.89, 1.85)
Whitehall II 351 1.34 (0.49, 3.65)
GOYA Females 521 1.36 (0.91, 2.02)
MIDSPAN 539 1.55 (0.86, 2.79)
The 1936 Cohort 202 2.18 (0.95, 4.97)
Subtotal (I-squared = 0.0%, p = 0.564) 1.06 (1.01, 1.11)
Ever smokers
ELSA 3447 0.84 (0.71, 0.98)
Inter99 3166 0.85 (0.70, 1.03)
GOYA Males 611 0.93 (0.51, 1.70)
Health2008 339 0.94 (0.55, 1.60)
NSHD 1751 1.01 (0.79, 1.28)
UK Biobank 51640 1.01 (0.97, 1.05)
FINRISK 11732 1.01 (0.89, 1.16)
ALSPAC Mothers 2119 1.01 (0.85, 1.20)
NEO 3609 1.03 (0.86, 1.23)
Health2006 1780 1.05 (0.85, 1.30)
MIDSPAN 1117 1.07 (0.71, 1.62)
HUNT2 23858 1.08 (1.01, 1.15)
KORA 468 1.08 (0.63, 1.87)
ALSPAC Children 274 1.09 (0.71, 1.65)
SHIP 2599 1.12 (0.94, 1.33)
Whitehall II 1081 1.13 (0.70, 1.82)
Dan-Monica10 1440 1.21 (0.91, 1.60)
1958 BC 2574 1.28 (1.05, 1.56)
SHIP TREND 565 1.34 (0.74, 2.42)
The 1936 Cohort 356 1.85 (0.97, 3.54)
Subtotal (I-squared = 23.0%, p = 0.172) 1.03 (1.00, 1.06)
.15
.3
.6
1
2
3
Odds ratio per smoking-increasing allele

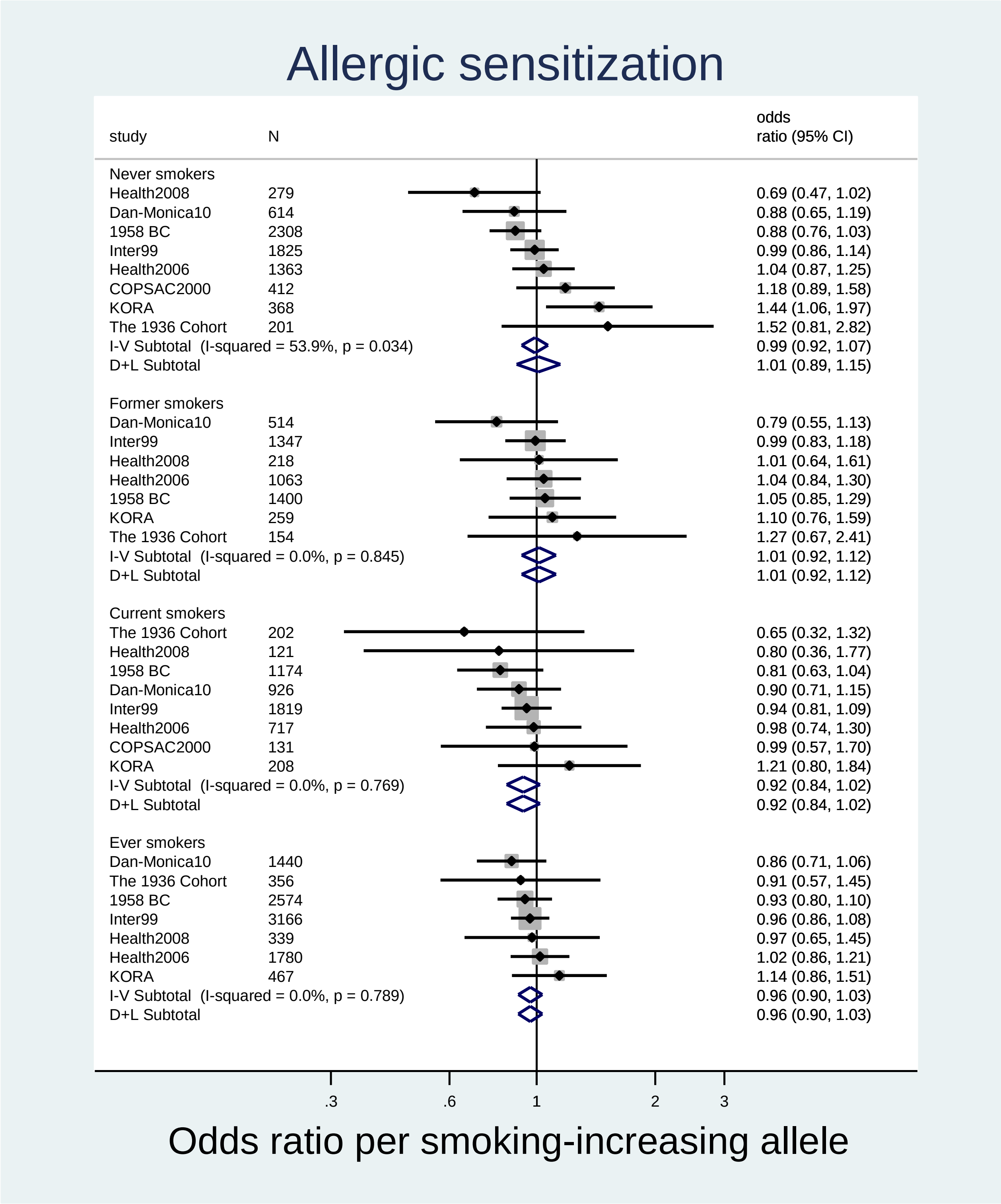
Allergic sensitization
study
N
odds ratio (95% CI)
Never smokers
Health2008 279 0.69 (0.47, 1.02)
Dan-Monica10 614 0.88 (0.65, 1.19)
1958 BC 2308 0.88 (0.76, 1.03)
Inter99 1825 0.99 (0.86, 1.14)
Health2006 1363 1.04 (0.87, 1.25)
COPSAC2000 412 1.18 (0.89, 1.58)
KORA 368 1.44 (1.06, 1.97)
The 1936 Cohort 201 1.52 (0.81, 2.82)
I-V Subtotal (I-squared = 53.9%, p = 0.034) 0.99 (0.92, 1.07)
D+L Subtotal 1.01 (0.89, 1.15)
Former smokers
Dan-Monica10 514 0.79 (0.55, 1.13)
Inter99 1347 0.99 (0.83, 1.18)
Health2008 218 1.01 (0.64, 1.61)
Health2006 1063 1.04 (0.84, 1.30)
1958 BC 1400 1.05 (0.85, 1.29)
KORA 259 1.10 (0.76, 1.59)
The 1936 Cohort 154 1.27 (0.67, 2.41)
I-V Subtotal (I-squared = 0.0%, p = 0.845) 1.01 (0.92, 1.12)
D+L Subtotal 1.01 (0.92, 1.12)
Current smokers
The 1936 Cohort 202 0.65 (0.32, 1.32)
Health2008 121 0.80 (0.36, 1.77)
1958 BC 1174 0.81 (0.63, 1.04)
Dan-Monica10 926 0.90 (0.71, 1.15)
Inter99 1819 0.94 (0.81, 1.09)
Health2006 717 0.98 (0.74, 1.30)
COPSAC2000 131 0.99 (0.57, 1.70)
KORA 208 1.21 (0.80, 1.84)
I-V Subtotal (I-squared = 0.0%, p = 0.769) 0.92 (0.84, 1.02)
D+L Subtotal 0.92 (0.84, 1.02)
Ever smokers
Dan-Monica10 1440 0.86 (0.71, 1.06)
The 1936 Cohort 356 0.91 (0.57, 1.45)
1958 BC 2574 0.93 (0.80, 1.10)
Inter99 3166 0.96 (0.86, 1.08)
Health2008 339 0.97 (0.65, 1.45)
Health2006 1780 1.02 (0.86, 1.21)
KORA 467 1.14 (0.86, 1.51)
I-V Subtotal (I-squared = 0.0%, p = 0.789) 0.96 (0.90, 1.03)
D+L Subtotal 0.96 (0.90, 1.03)
.3
.6
1
2
3
Odds ratio per smoking-increasing allele

Figure S16

Crude association of genotype and smoking heaviness.

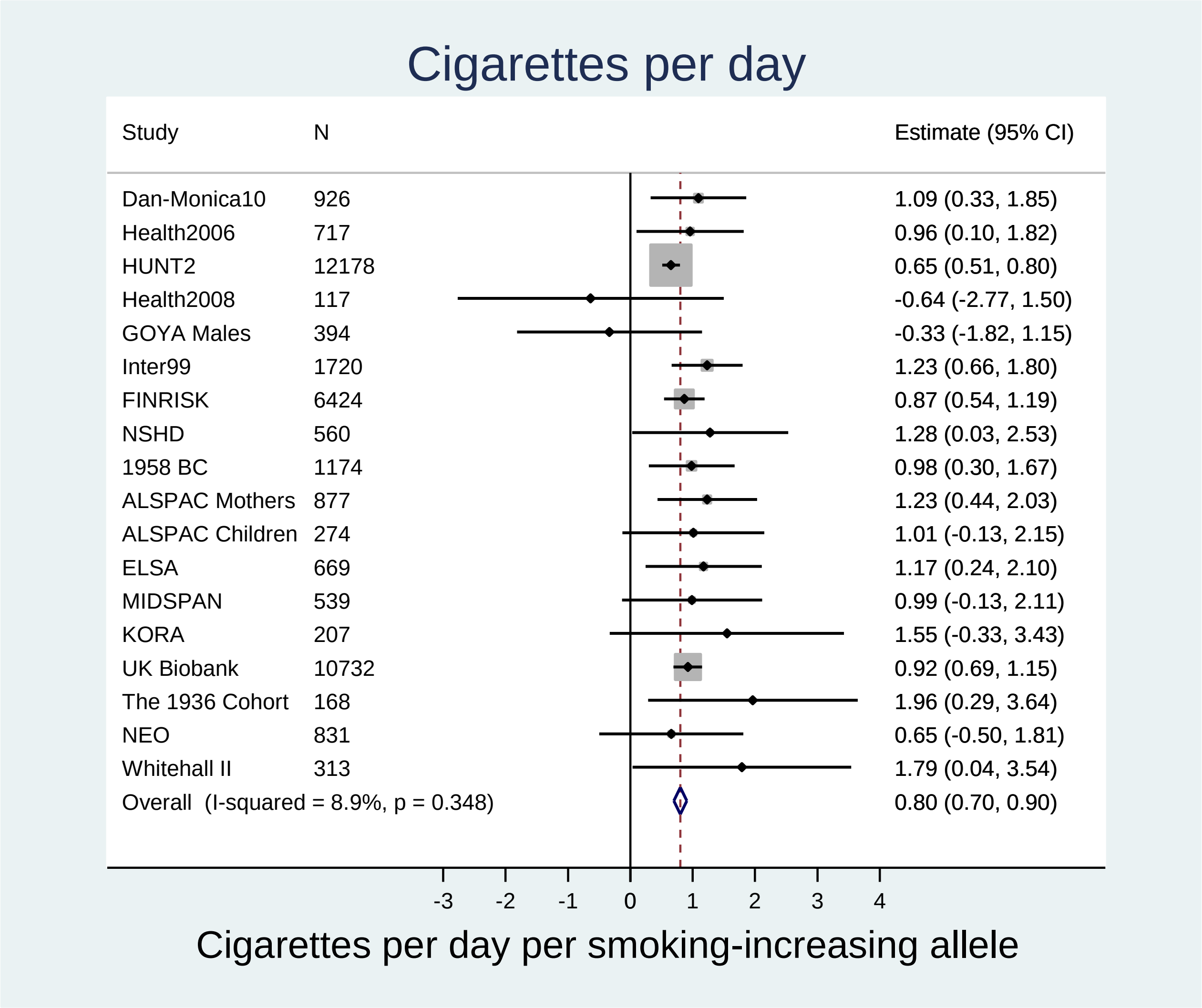

Additional analyses

Figure S17-S18

Mendelian randomization analysis of the age- and sex- adjusted associations of rs1051730/rs16969968 and hay fever and asthma, excluding the UK Biobank data.

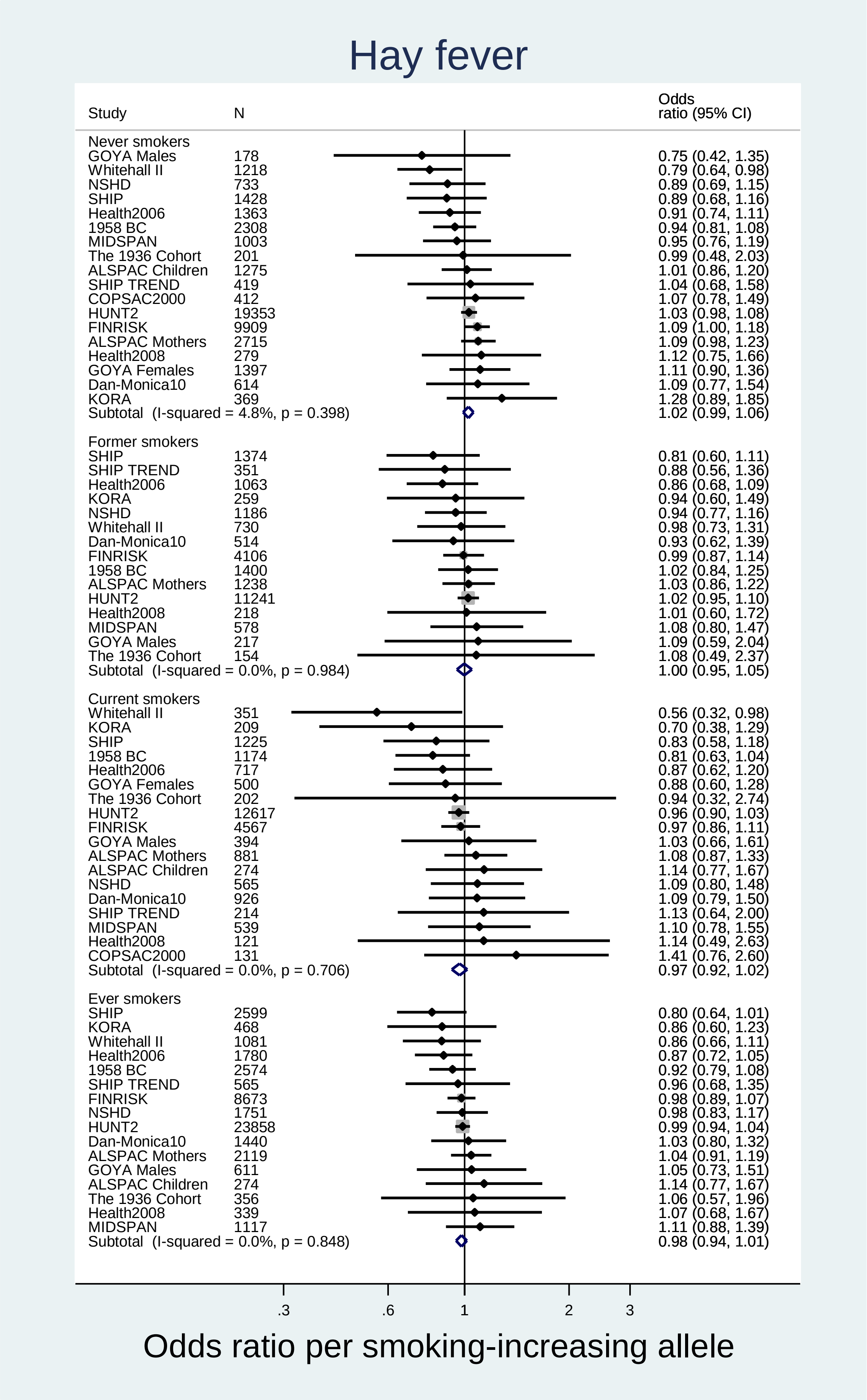
Hay fever
Study N Odds ratio (95% CI)
Never smokers
GOYA Males 178 0.75 (0.42, 1.35)
Whitehall II 1218 0.79 (0.64, 0.98)
NSHD 733 0.89 (0.69, 1.15)
SHIP 1428 0.89 (0.68, 1.16)
Health2006 1363 0.91 (0.74, 1.11)
1958 BC 2308 0.94 (0.81, 1.08)
MIDSPAN 1003 0.95 (0.76, 1.19)
The 1936 Cohort 201 0.99 (0.48, 2.03)
ALSPAC Children 1275 1.01 (0.86, 1.20)
SHIP TREND 419 1.04 (0.68, 1.58)
COPSAC2000 412 1.07 (0.78, 1.49)
HUNT2 19353 1.03 (0.98, 1.08)
FINRISK 9909 1.09 (1.00, 1.18)
ALSPAC Mothers 2715 1.09 (0.98, 1.23)
Health2008 279 1.12 (0.75, 1.66)
GOYA Females 1397 1.11 (0.90, 1.36)
Dan-Monica10 614 1.09 (0.77, 1.54)
KORA 369 1.28 (0.89, 1.85)
Subtotal (I-squared = 4.8%, p = 0.398) 1.02 (0.99, 1.06)
Former smokers
SHIP 1374 0.81 (0.60, 1.11)
SHIP TREND 351 0.88 (0.56, 1.36)
Health2006 1063 0.86 (0.68, 1.09)
KORA 259 0.94 (0.60, 1.49)
NSHD 1186 0.94 (0.77, 1.16)
Whitehall II 730 0.98 (0.73, 1.31)
Dan-Monica10 514 0.93 (0.62, 1.39)
FINRISK 4106 0.99 (0.87, 1.14)
1958 BC 1400 1.02 (0.84, 1.25)
ALSPAC Mothers 1238 1.03 (0.86, 1.22)
HUNT2 11241 1.02 (0.95, 1.10)
Health2008 218 1.01 (0.60, 1.72)
MIDSPAN 578 1.08 (0.80, 1.47)
GOYA Males 217 1.09 (0.59, 2.04)
The 1936 Cohort 154 1.08 (0.49, 2.37)
Subtotal (I-squared = 0.0%, p = 0.984) 1.00 (0.95, 1.05)
Current smokers
Whitehall II 351 0.56 (0.32, 0.98)
KORA 209 0.70 (0.38, 1.29)
SHIP 1225 0.83 (0.58, 1.18)
1958 BC 1174 0.81 (0.63, 1.04)
Health2006 717 0.87 (0.62, 1.20)
GOYA Females 500 0.88 (0.60, 1.28)
The 1936 Cohort 202 0.94 (0.32, 2.74)
HUNT2 12617 0.96 (0.90, 1.03)
FINRISK 4567 0.97 (0.86, 1.11)
GOYA Males 394 1.03 (0.66, 1.61)
ALSPAC Mothers 881 1.08 (0.87, 1.33)
ALSPAC Children 274 1.14 (0.77, 1.67)
NSHD 565 1.09 (0.80, 1.48)
Dan-Monica10 926 1.09 (0.79, 1.50)
SHIP TREND 214 1.13 (0.64, 2.00)
MIDSPAN 539 1.10 (0.78, 1.55)
Health2008 121 1.14 (0.49, 2.63)
COPSAC2000 131 1.41 (0.76, 2.60)
Subtotal (I-squared = 0.0%, p = 0.706) 0.97 (0.92, 1.02)
Ever smokers
SHIP 2599 0.80 (0.64, 1.01)
KORA 468 0.86 (0.60, 1.23)
Whitehall II 1081 0.86 (0.66, 1.11)
Health2006 1780 0.87 (0.72, 1.05)
1958 BC 2574 0.92 (0.79, 1.08)
SHIP TREND 565 0.96 (0.68, 1.35)
FINRISK 8673 0.98 (0.89, 1.07)
NSHD 1751 0.98 (0.83, 1.17)
HUNT2 23858 0.99 (0.94, 1.04)
Dan-Monica10 1440 1.03 (0.80, 1.32)
ALSPAC Mothers 2119 1.04 (0.91, 1.19)
GOYA Males 611 1.05 (0.73, 1.51)
ALSPAC Children 274 1.14 (0.77, 1.67)
The 1936 Cohort 356 1.06 (0.57, 1.96)
Health2008 339 1.07 (0.68, 1.67)
MIDSPAN 1117 1.11 (0.88, 1.39)
Subtotal (I-squared = 0.0%, p = 0.848) 0.98 (0.94, 1.01)
.3
.6
1
2
3
Odds ratio per smoking-increasing allele

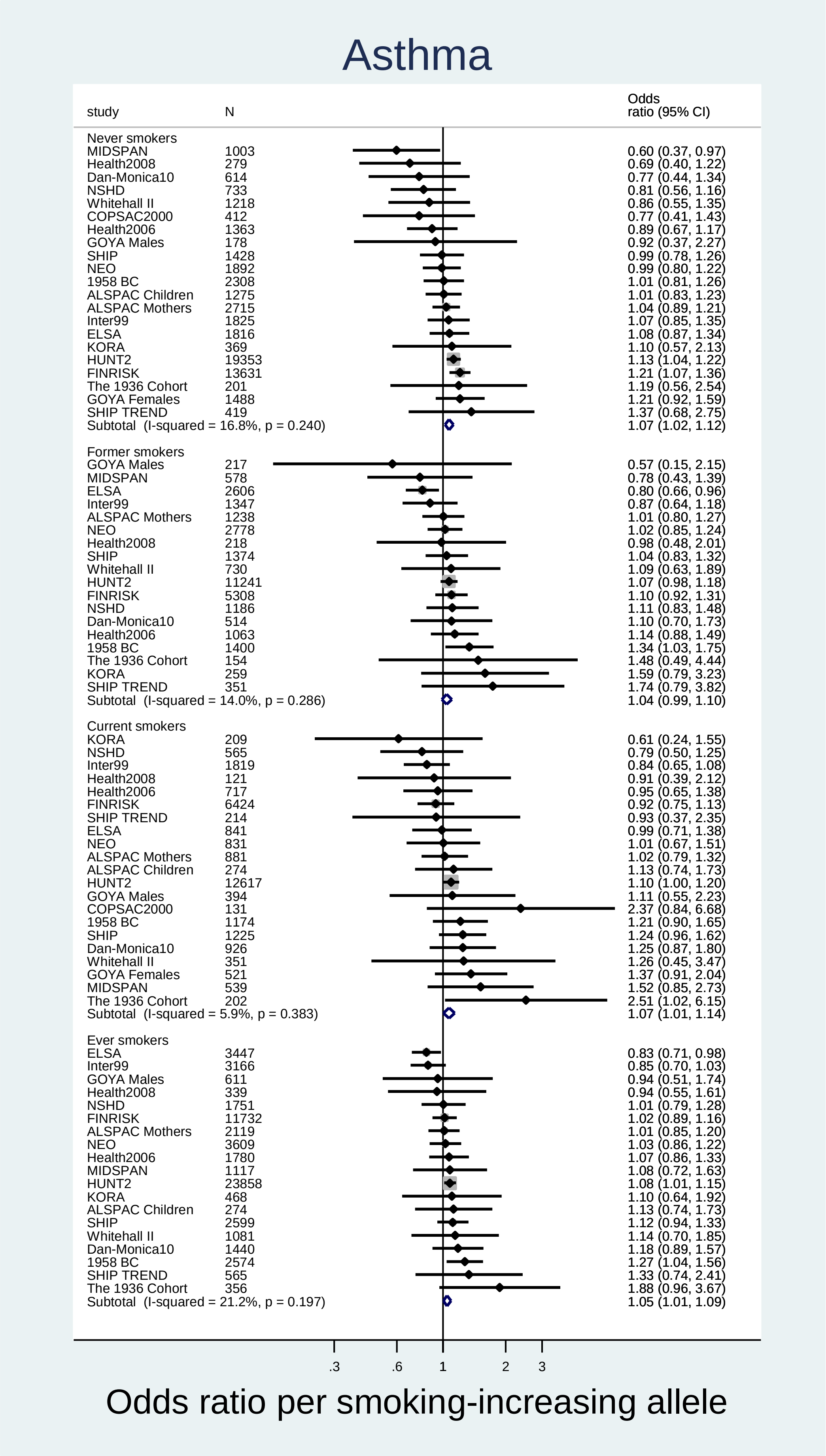
Asthma
study
N
Odds ratio (95% CI)
Never smokers
MIDSPAN 1003 0.60 (0.37, 0.97)
Health2008 279 0.69 (0.40, 1.22)
Dan-Monica10 614 0.77 (0.44, 1.34)
NSHD 733 0.81 (0.56, 1.16)
Whitehall II 1218 0.86 (0.55, 1.35)
COPSAC2000 412 0.77 (0.41, 1.43)
Health2006 1363 0.89 (0.67, 1.17)
GOYA Males 178 0.92 (0.37, 2.27)
SHIP 1428 0.99 (0.78, 1.26)
NEO 1892 0.99 (0.80, 1.22)
1958 BC 2308 1.01 (0.81, 1.26)
ALSPAC Children 1275 1.01 (0.83, 1.23)
ALSPAC Mothers 2715 1.04 (0.89, 1.21)
Inter99 1825 1.07 (0.85, 1.35)
ELSA 1816 1.08 (0.87, 1.34)
KORA 369 1.10 (0.57, 2.13)
HUNT2 19353 1.13 (1.04, 1.22)
FINRISK 13631 1.21 (1.07, 1.36)
The 1936 Cohort 201 1.19 (0.56, 2.54)
GOYA Females 1488 1.21 (0.92, 1.59)
SHIP TREND 419 1.37 (0.68, 2.75)
Subtotal (I-squared = 16.8%, p = 0.240) 1.07 (1.02, 1.12)
Former smokers
GOYA Males 217 0.57 (0.15, 2.15)
MIDSPAN 578 0.78 (0.43, 1.39)
ELSA 2606 0.80 (0.66, 0.96)
Inter99 1347 0.87 (0.64, 1.18)
ALSPAC Mothers 1238 1.01 (0.80, 1.27)
NEO 2778 1.02 (0.85, 1.24)
Health2008 218 0.98 (0.48, 2.01)
SHIP 1374 1.04 (0.83, 1.32)
Whitehall II 730 1.09 (0.63, 1.89)
HUNT2 11241 1.07 (0.98, 1.18)
FINRISK 5308 1.10 (0.92, 1.31)
NSHD 1186 1.11 (0.83, 1.48)
Dan-Monica10 514 1.10 (0.70, 1.73)
Health2006 1063 1.14 (0.88, 1.49)
1958 BC 1400 1.34 (1.03, 1.75)
The 1936 Cohort 154 1.48 (0.49, 4.44)
KORA 259 1.59 (0.79, 3.23)
SHIP TREND 351 1.74 (0.79, 3.82)
Subtotal (I-squared = 14.0%, p = 0.286) 1.04 (0.99, 1.10)
Current smokers
KORA 209 0.61 (0.24, 1.55)
NSHD 565 0.79 (0.50, 1.25)
Inter99 1819 0.84 (0.65, 1.08)
Health2008 121 0.91 (0.39, 2.12)
Health2006 717 0.95 (0.65, 1.38)
FINRISK 6424 0.92 (0.75, 1.13)
SHIP TREND 214 0.93 (0.37, 2.35)
ELSA 841 0.99 (0.71, 1.38)
NEO 831 1.01 (0.67, 1.51)
ALSPAC Mothers 881 1.02 (0.79, 1.32)
ALSPAC Children 274 1.13 (0.74, 1.73)
HUNT2 12617 1.10 (1.00, 1.20)
GOYA Males 394 1.11 (0.55, 2.23)
COPSAC2000 131 2.37 (0.84, 6.68)
1958 BC 1174 1.21 (0.90, 1.65)
SHIP 1225 1.24 (0.96, 1.62)
Dan-Monica10 926 1.25 (0.87, 1.80)
Whitehall II 351 1.26 (0.45, 3.47)
GOYA Females 521 1.37 (0.91, 2.04)
MIDSPAN 539 1.52 (0.85, 2.73)
The 1936 Cohort 202 2.51 (1.02, 6.15)
Subtotal (I-squared = 5.9%, p = 0.383) 1.07 (1.01, 1.14)
Ever smokers
ELSA 3447 0.83 (0.71, 0.98)
Inter99 3166 0.85 (0.70, 1.03)
GOYA Males 611 0.94 (0.51, 1.74)
Health2008 339 0.94 (0.55, 1.61)
NSHD 1751 1.01 (0.79, 1.28)
FINRISK 11732 1.02 (0.89, 1.16)
ALSPAC Mothers 2119 1.01 (0.85, 1.20)
NEO 3609 1.03 (0.86, 1.22)
Health2006 1780 1.07 (0.86, 1.33)
MIDSPAN 1117 1.08 (0.72, 1.63)
HUNT2 23858 1.08 (1.01, 1.15)
KORA 468 1.10 (0.64, 1.92)
ALSPAC Children 274 1.13 (0.74, 1.73)
SHIP 2599 1.12 (0.94, 1.33)
Whitehall II 1081 1.14 (0.70, 1.85)
Dan-Monica10 1440 1.18 (0.89, 1.57)
1958 BC 2574 1.27 (1.04, 1.56)
SHIP TREND 565 1.33 (0.74, 2.41)
The 1936 Cohort 356 1.88 (0.96, 3.67)
Subtotal (I-squared = 21.2%, p = 0.197) 1.05 (1.01, 1.09)
.3
.6
1
2
3
Odds ratio per smoking-increasing allele

Figure S19-S20

Age- and sex- adjusted association of the rs1051730/rs16969968 SNP with hay fever and asthma in selected samples of the UK Biobank data.

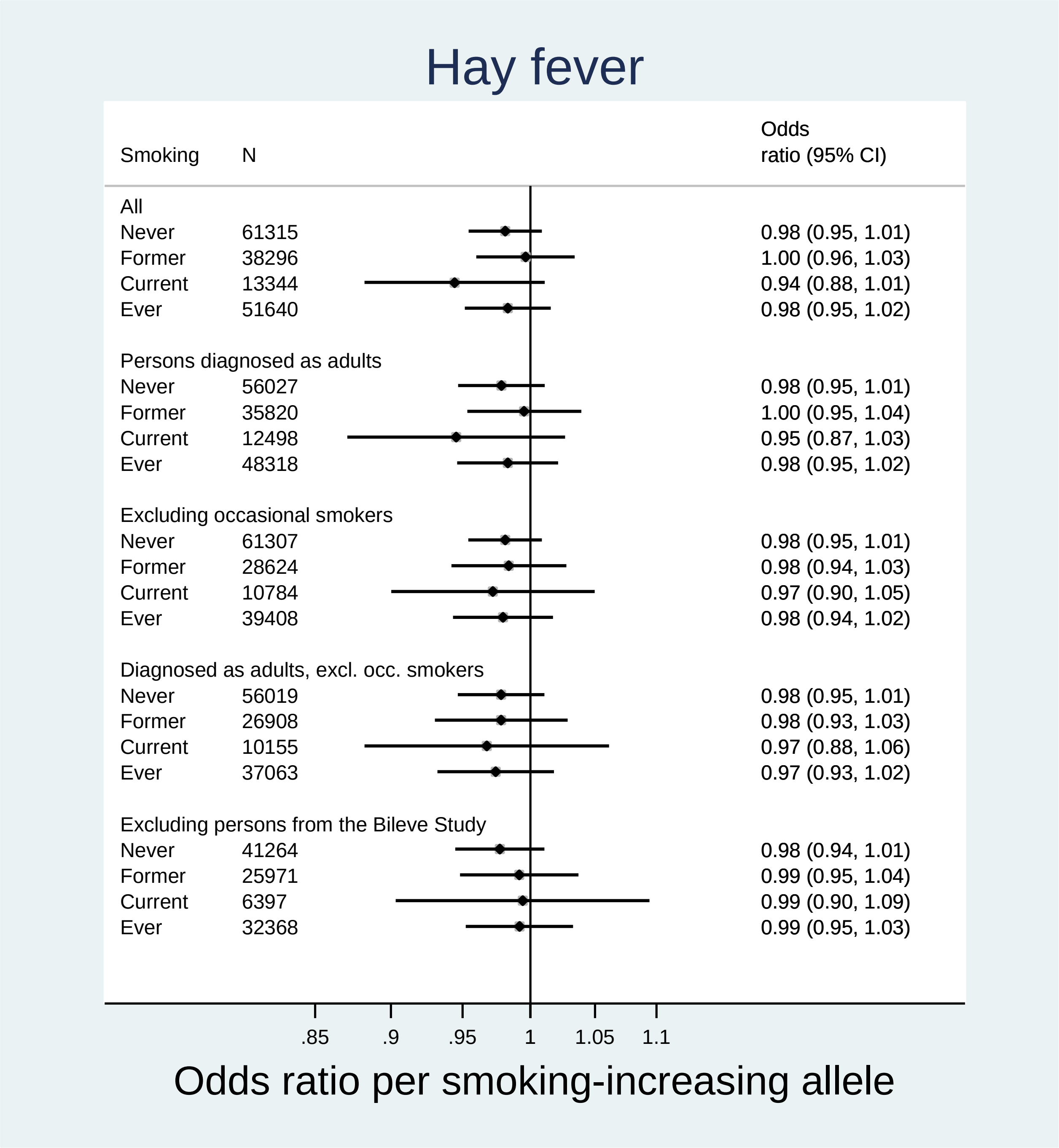

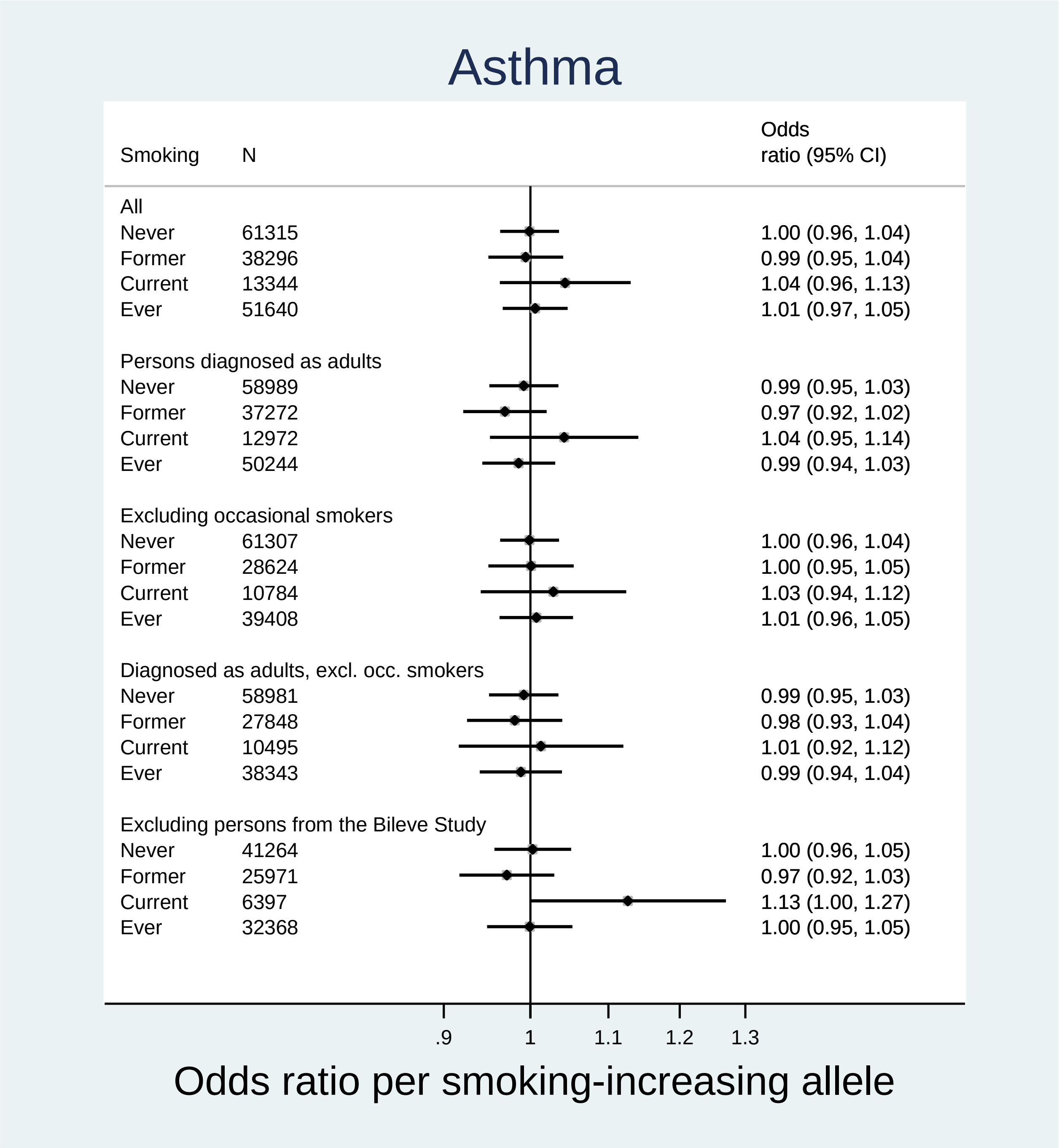
Asthma
Smoking
N
Odds ratio (95% CI)
All
Never 61315 1.00 (0.96, 1.04)
Former 38296 0.99 (0.95, 1.04)
Current 13344 1.04 (0.96, 1.13)
Ever 51640 1.01 (0.97, 1.05)
Persons diagnosed as adults
Never 58989 0.99 (0.95, 1.03)
Former 37272 0.97 (0.92, 1.02)
Current 12972 1.04 (0.95, 1.14)
Ever 50244 0.99 (0.94, 1.03)
Excluding occasional smokers
Never 61307 1.00 (0.96, 1.04)
Former 28624 1.00 (0.95, 1.05)
Current 10784 1.03 (0.94, 1.12)
Ever 39408 1.01 (0.96, 1.05)
Diagnosed as adults, excl. occ. smokers
Never 58981 0.99 (0.95, 1.03)
Former 27848 0.98 (0.93, 1.04)
Current 10495 1.01 (0.92, 1.12)
Ever 38343 0.99 (0.94, 1.04)
Excluding persons from the Bileve Study
Never 41264 1.00 (0.96, 1.05)
Former 25971 0.97 (0.92, 1.03)
Current 6397 1.13 (1.00, 1.27)
Ever 32368 1.00 (0.95, 1.05)
.9
1
1.1
1.2
1.3
Odds ratio per smoking-increasing allele

Figure S21-S22

Age- and sex- adjusted association of the rs1051730/rs16969968 SNP with hay fever and asthma, excluding the ALSPAC Mothers.

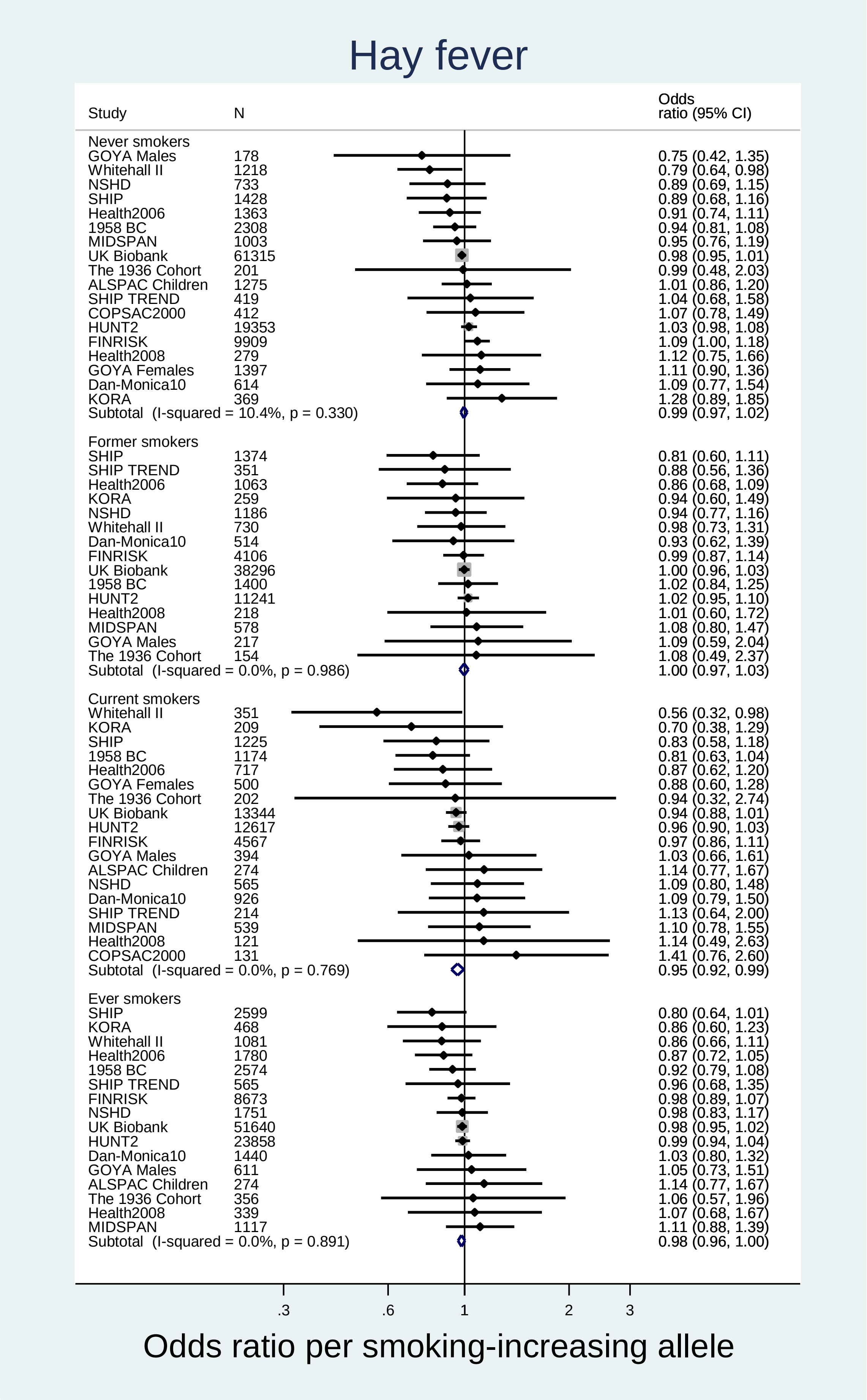
Hay fever
Study
N
Odds ratio (95% CI)
Never smokers
GOYA Males 178 0.75 (0.42, 1.35)
Whitehall II 1218 0.79 (0.64, 0.98)
NSHD 733 0.89 (0.69, 1.15)
SHIP 1428 0.89 (0.68, 1.16)
Health2006 1363 0.91 (0.74, 1.11)
1958 BC 2308 0.94 (0.81, 1.08)
MIDSPAN 1003 0.95 (0.76, 1.19)
UK Biobank 61315 0.98 (0.95, 1.01)
The 1936 Cohort 201 0.99 (0.48, 2.03)
ALSPAC Children 1275 1.01 (0.86, 1.20)
SHIP TREND 419 1.04 (0.68, 1.58)
COPSAC2000 412 1.07 (0.78, 1.49)
HUNT2 19353 1.03 (0.98, 1.08)
FINRISK 9909 1.09 (1.00, 1.18)
Health2008 279 1.12 (0.75, 1.66)
GOYA Females 1397 1.11 (0.90, 1.36)
Dan-Monica10 614 1.09 (0.77, 1.54)
KORA 369 1.28 (0.89, 1.85)
Subtotal (I-squared = 10.4%, p = 0.330) 0.99 (0.97, 1.02)
Former smokers
SHIP 1374 0.81 (0.60, 1.11)
SHIP TREND 351 0.88 (0.56, 1.36)
Health2006 1063 0.86 (0.68, 1.09)
KORA 259 0.94 (0.60, 1.49)
NSHD 1186 0.94 (0.77, 1.16)
Whitehall II 730 0.98 (0.73, 1.31)
Dan-Monica10 514 0.93 (0.62, 1.39)
FINRISK 4106 0.99 (0.87, 1.14)
UK Biobank 38296 1.00 (0.96, 1.03)
1958 BC 1400 1.02 (0.84, 1.25)
HUNT2 11241 1.02 (0.95, 1.10)
Health2008 218 1.01 (0.60, 1.72)
MIDSPAN 578 1.08 (0.80, 1.47)
GOYA Males 217 1.09 (0.59, 2.04)
The 1936 Cohort 154 1.08 (0.49, 2.37)
Subtotal (I-squared = 0.0%, p = 0.986) 1.00 (0.97, 1.03)
Current smokers
Whitehall II 351 0.56 (0.32, 0.98)
KORA 209 0.70 (0.38, 1.29)
SHIP 1225 0.83 (0.58, 1.18)
1958 BC 1174 0.81 (0.63, 1.04)
Health2006 717 0.87 (0.62, 1.20)
GOYA Females 500 0.88 (0.60, 1.28)
The 1936 Cohort 202 0.94 (0.32, 2.74)
UK Biobank 13344 0.94 (0.88, 1.01)
HUNT2 12617 0.96 (0.90, 1.03)
FINRISK 4567 0.97 (0.86, 1.11)
GOYA Males 394 1.03 (0.66, 1.61)
ALSPAC Children 274 1.14 (0.77, 1.67)
NSHD 565 1.09 (0.80, 1.48)
Dan-Monica10 926 1.09 (0.79, 1.50)
SHIP TREND 214 1.13 (0.64, 2.00)
MIDSPAN 539 1.10 (0.78, 1.55)
Health2008 121 1.14 (0.49, 2.63)
COPSAC2000 131 1.41 (0.76, 2.60)
Subtotal (I-squared = 0.0%, p = 0.769) 0.95 (0.92, 0.99)
Ever smokers
SHIP 2599 0.80 (0.64, 1.01)
KORA 468 0.86 (0.60, 1.23)
Whitehall II 1081 0.86 (0.66, 1.11)
Health2006 1780 0.87 (0.72, 1.05)
1958 BC 2574 0.92 (0.79, 1.08)
SHIP TREND 565 0.96 (0.68, 1.35)
FINRISK 8673 0.98 (0.89, 1.07)
NSHD 1751 0.98 (0.83, 1.17)
UK Biobank 51640 0.98 (0.95, 1.02)
HUNT2 23858 0.99 (0.94, 1.04)
Dan-Monica10 1440 1.03 (0.80, 1.32)
GOYA Males 611 1.05 (0.73, 1.51)
ALSPAC Children 274 1.14 (0.77, 1.67)
The 1936 Cohort 356 1.06 (0.57, 1.96)
Health2008 339 1.07 (0.68, 1.67)
MIDSPAN 1117 1.11 (0.88, 1.39)
Subtotal (I-squared = 0.0%, p = 0.891) 0.98 (0.96, 1.00)
.3
.6
1
2
3
Odds ratio per smoking-increasing allele

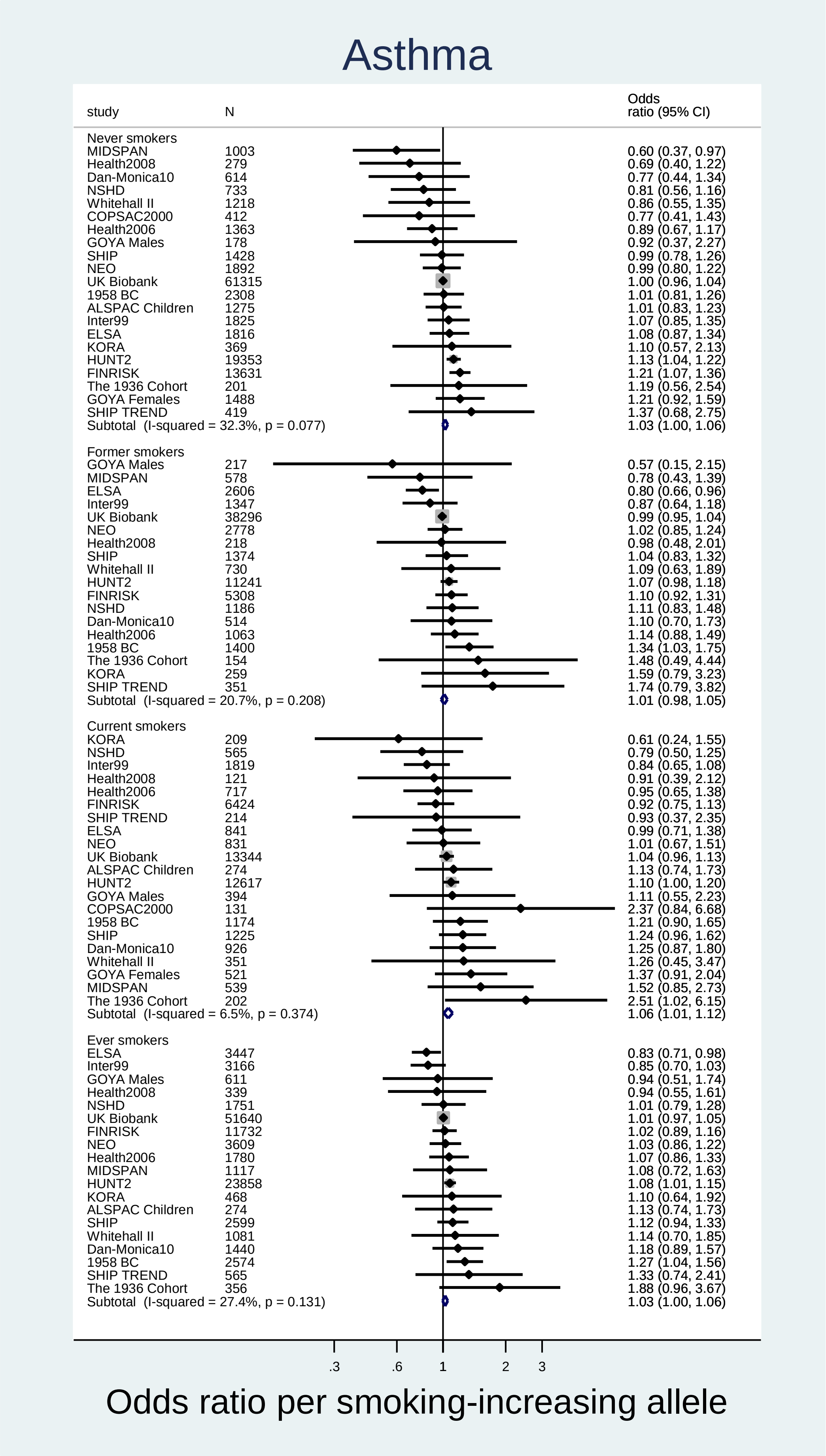
Asthma
study N Odds ratio (95% CI)
Never smokers
MIDSPAN 1003 0.60 (0.37, 0.97)
Health2008 279 0.69 (0.40, 1.22)
Dan-Monica10 614 0.77 (0.44, 1.34)
NSHD 733 0.81 (0.56, 1.16)
Whitehall II 1218 0.86 (0.55, 1.35)
COPSAC2000 412 0.77 (0.41, 1.43)
Health2006 1363 0.89 (0.67, 1.17)
GOYA Males 178 0.92 (0.37, 2.27)
SHIP 1428 0.99 (0.78, 1.26)
NEO 1892 0.99 (0.80, 1.22)
UK Biobank 61315 1.00 (0.96, 1.04)
1958 BC 2308 1.01 (0.81, 1.26)
ALSPAC Children 1275 1.01 (0.83, 1.23)
Inter99 1825 1.07 (0.85, 1.35)
ELSA 1816 1.08 (0.87, 1.34)
KORA 369 1.10 (0.57, 2.13)
HUNT2 19353 1.13 (1.04, 1.22)
FINRISK 13631 1.21 (1.07, 1.36)
The 1936 Cohort 201 1.19 (0.56, 2.54)
GOYA Females 1488 1.21 (0.92, 1.59)
SHIP TREND 419 1.37 (0.68, 2.75)
Subtotal (I-squared = 32.3%, p = 0.077) 1.03 (1.00, 1.06)
Former smokers
GOYA Males 217 0.57 (0.15, 2.15)
MIDSPAN 578 0.78 (0.43, 1.39)
ELSA 2606 0.80 (0.66, 0.96)
Inter99 1347 0.87 (0.64, 1.18)
UK Biobank 38296 0.99 (0.95, 1.04)
NEO 2778 1.02 (0.85, 1.24)
Health2008 218 0.98 (0.48, 2.01)
SHIP 1374 1.04 (0.83, 1.32)
Whitehall II 730 1.09 (0.63, 1.89)
HUNT2 11241 1.07 (0.98, 1.18)
FINRISK 5308 1.10 (0.92, 1.31)
NSHD 1186 1.11 (0.83, 1.48)
Dan-Monica10 514 1.10 (0.70, 1.73)
Health2006 1063 1.14 (0.88, 1.49)
1958 BC 1400 1.34 (1.03, 1.75)
The 1936 Cohort 154 1.48 (0.49, 4.44)
KORA 259 1.59 (0.79, 3.23)
SHIP TREND 351 1.74 (0.79, 3.82)
Subtotal (I-squared = 20.7%, p = 0.208) 1.01 (0.98, 1.05)
Current smokers
KORA 209 0.61 (0.24, 1.55)
NSHD 565 0.79 (0.50, 1.25)
Inter99 1819 0.84 (0.65, 1.08)
Health2008 121 0.91 (0.39, 2.12)
Health2006 717 0.95 (0.65, 1.38)
FINRISK 6424 0.92 (0.75, 1.13)
SHIP TREND 214 0.93 (0.37, 2.35)
ELSA 841 0.99 (0.71, 1.38)
NEO 831 1.01 (0.67, 1.51)
UK Biobank 13344 1.04 (0.96, 1.13)
ALSPAC Children 274 1.13 (0.74, 1.73)
HUNT2 12617 1.10 (1.00, 1.20)
GOYA Males 394 1.11 (0.55, 2.23)
COPSAC2000 131 2.37 (0.84, 6.68)
1958 BC 1174 1.21 (0.90, 1.65)
SHIP 1225 1.24 (0.96, 1.62)
Dan-Monica10 926 1.25 (0.87, 1.80)
Whitehall II 351 1.26 (0.45, 3.47)
GOYA Females 521 1.37 (0.91, 2.04)
MIDSPAN 539 1.52 (0.85, 2.73)
The 1936 Cohort 202 2.51 (1.02, 6.15)
Subtotal (I-squared = 6.5%, p = 0.374) 1.06 (1.01, 1.12)
Ever smokers
ELSA 3447 0.83 (0.71, 0.98)
Inter99 3166 0.85 (0.70, 1.03)
GOYA Males 611 0.94 (0.51, 1.74)
Health2008 339 0.94 (0.55, 1.61)
NSHD 1751 1.01 (0.79, 1.28)
UK Biobank 51640 1.01 (0.97, 1.05)
FINRISK 11732 1.02 (0.89, 1.16)
NEO 3609 1.03 (0.86, 1.22)
Health2006 1780 1.07 (0.86, 1.33)
MIDSPAN 1117 1.08 (0.72, 1.63)
HUNT2 23858 1.08 (1.01, 1.15)
KORA 468 1.10 (0.64, 1.92)
ALSPAC Children 274 1.13 (0.74, 1.73)
SHIP 2599 1.12 (0.94, 1.33)
Whitehall II 1081 1.14 (0.70, 1.85)
Dan-Monica10 1440 1.18 (0.89, 1.57)
1958 BC 2574 1.27 (1.04, 1.56)
SHIP TREND 565 1.33 (0.74, 2.41)
The 1936 Cohort 356 1.88 (0.96, 3.67)
Subtotal (I-squared = 27.4%, p = 0.131) 1.03 (1.00, 1.06)
.3
.6
1
2
3
Odds ratio per smoking-increasing allele

Figure S23-S24

Age- and sex- adjusted association of the rs1051730/rs16969968 SNP with hay fever and asthma, excluding the ALSPAC Children.

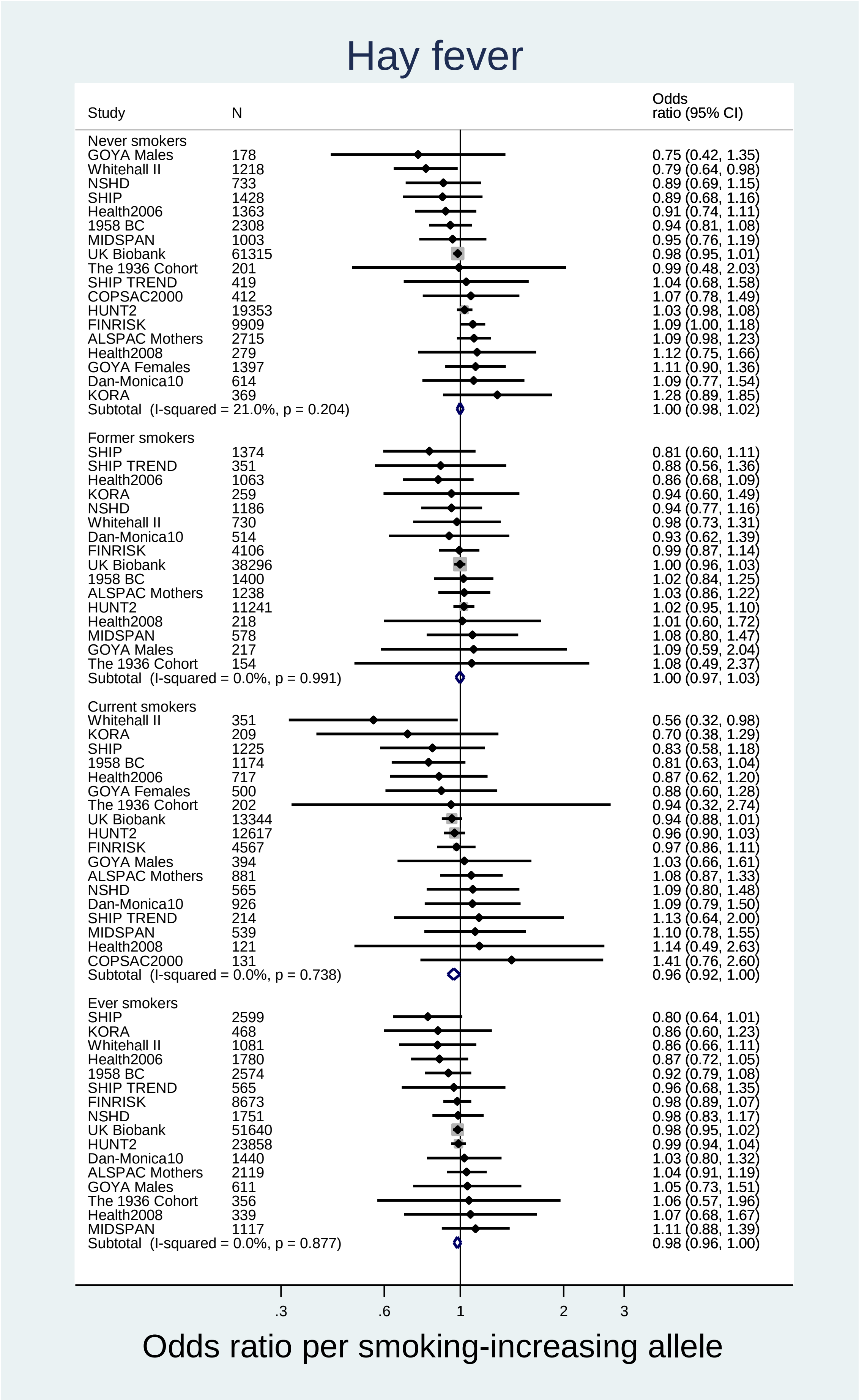
Hay fever
Study N Odds ratio (95% CI)
Never smokers
GOYA Males 178 0.75 (0.42, 1.35)
Whitehall II 1218 0.79 (0.64, 0.98)
NSHD 733 0.89 (0.69, 1.15)
SHIP 1428 0.89 (0.68, 1.16)
Health2006 1363 0.91 (0.74, 1.11)
1958 BC 2308 0.94 (0.81, 1.08)
MIDSPAN 1003 0.95 (0.76, 1.19)
UK Biobank 61315 0.98 (0.95, 1.01)
The 1936 Cohort 201 0.99 (0.48, 2.03)
SHIP TREND 419 1.04 (0.68, 1.58)
COPSAC2000 412 1.07 (0.78, 1.49)
HUNT2 19353 1.03 (0.98, 1.08)
FINRISK 9909 1.09 (1.00, 1.18)
ALSPAC Mothers 2715 1.09 (0.98, 1.23)
Health2008 279 1.12 (0.75, 1.66)
GOYA Females 1397 1.11 (0.90, 1.36)
Dan-Monica10 614 1.09 (0.77, 1.54)
KORA 369 1.28 (0.89, 1.85)
Subtotal (I-squared = 21.0%, p = 0.204) 1.00 (0.98, 1.02)
Former smokers
SHIP 1374 0.81 (0.60, 1.11)
SHIP TREND 351 0.88 (0.56, 1.36)
Health2006 1063 0.86 (0.68, 1.09)
KORA 259 0.94 (0.60, 1.49)
NSHD 1186 0.94 (0.77, 1.16)
Whitehall II 730 0.98 (0.73, 1.31)
Dan-Monica10 514 0.93 (0.62, 1.39)
FINRISK 4106 0.99 (0.87, 1.14)
UK Biobank 38296 1.00 (0.96, 1.03)
1958 BC 1400 1.02 (0.84, 1.25)
ALSPAC Mothers 1238 1.03 (0.86, 1.22)
HUNT2 11241 1.02 (0.95, 1.10)
Health2008 218 1.01 (0.60, 1.72)
MIDSPAN 578 1.08 (0.80, 1.47)
GOYA Males 217 1.09 (0.59, 2.04)
The 1936 Cohort 154 1.08 (0.49, 2.37)
Subtotal (I-squared = 0.0%, p = 0.991) 1.00 (0.97, 1.03)
Current smokers
Whitehall II 351 0.56 (0.32, 0.98)
KORA 209 0.70 (0.38, 1.29)
SHIP 1225 0.83 (0.58, 1.18)
1958 BC 1174 0.81 (0.63, 1.04)
Health2006 717 0.87 (0.62, 1.20)
GOYA Females 500 0.88 (0.60, 1.28)
The 1936 Cohort 202 0.94 (0.32, 2.74)
UK Biobank 13344 0.94 (0.88, 1.01)
HUNT2 12617 0.96 (0.90, 1.03)
FINRISK 4567 0.97 (0.86, 1.11)
GOYA Males 394 1.03 (0.66, 1.61)
ALSPAC Mothers 881 1.08 (0.87, 1.33)
NSHD 565 1.09 (0.80, 1.48)
Dan-Monica10 926 1.09 (0.79, 1.50)
SHIP TREND 214 1.13 (0.64, 2.00)
MIDSPAN 539 1.10 (0.78, 1.55)
Health2008 121 1.14 (0.49, 2.63)
COPSAC2000 131 1.41 (0.76, 2.60)
Subtotal (I-squared = 0.0%, p = 0.738) 0.96 (0.92, 1.00)
Ever smokers
SHIP 2599 0.80 (0.64, 1.01)
KORA 468 0.86 (0.60, 1.23)
Whitehall II 1081 0.86 (0.66, 1.11)
Health2006 1780 0.87 (0.72, 1.05)
1958 BC 2574 0.92 (0.79, 1.08)
SHIP TREND 565 0.96 (0.68, 1.35)
FINRISK 8673 0.98 (0.89, 1.07)
NSHD 1751 0.98 (0.83, 1.17)
UK Biobank 51640 0.98 (0.95, 1.02)
HUNT2 23858 0.99 (0.94, 1.04)
Dan-Monica10 1440 1.03 (0.80, 1.32)
ALSPAC Mothers 2119 1.04 (0.91, 1.19)
GOYA Males 611 1.05 (0.73, 1.51)
The 1936 Cohort 356 1.06 (0.57, 1.96)
Health2008 339 1.07 (0.68, 1.67)
MIDSPAN 1117 1.11 (0.88, 1.39)
Subtotal (I-squared = 0.0%, p = 0.877) 0.98 (0.96, 1.00)
.3
.6
1
2
3
Odds ratio per smoking-increasing allele

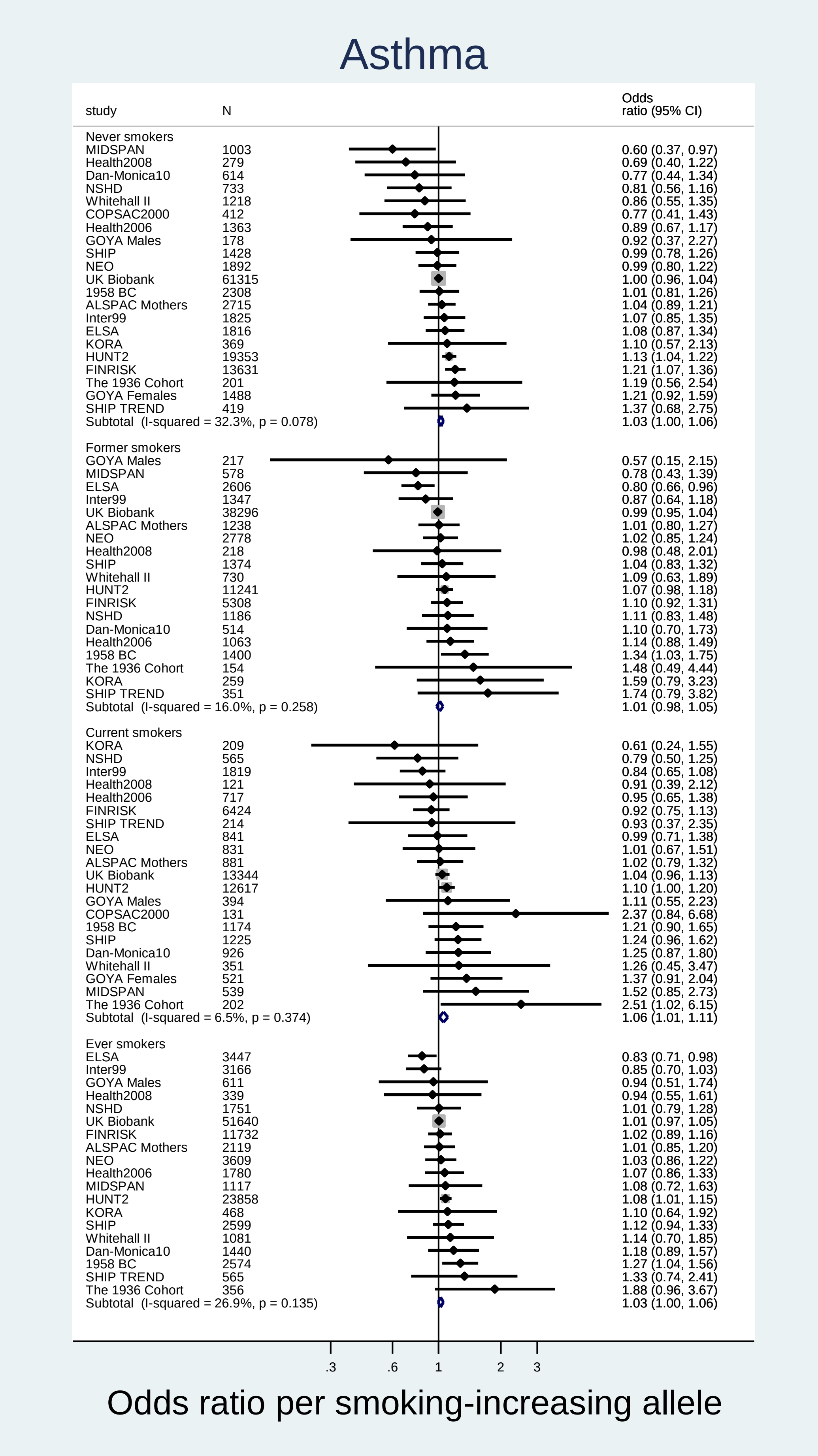
Asthma
study N Odds ratio (95% CI)
Never smokers
MIDSPAN 1003 0.60 (0.37, 0.97)
Health2008 279 0.69 (0.40, 1.22)
Dan-Monica10 614 0.77 (0.44, 1.34)
NSHD 733 0.81 (0.56, 1.16)
Whitehall II 1218 0.86 (0.55, 1.35)
COPSAC2000 412 0.77 (0.41, 1.43)
Health2006 1363 0.89 (0.67, 1.17)
GOYA Males 178 0.92 (0.37, 2.27)
SHIP 1428 0.99 (0.78, 1.26)
NEO 1892 0.99 (0.80, 1.22)
UK Biobank 61315 1.00 (0.96, 1.04)
1958 BC 2308 1.01 (0.81, 1.26)
ALSPAC Mothers 2715 1.04 (0.89, 1.21)
Inter99 1825 1.07 (0.85, 1.35)
ELSA 1816 1.08 (0.87, 1.34)
KORA 369 1.10 (0.57, 2.13)
HUNT2 19353 1.13 (1.04, 1.22)
FINRISK 13631 1.21 (1.07, 1.36)
The 1936 Cohort 201 1.19 (0.56, 2.54)
GOYA Females 1488 1.21 (0.92, 1.59)
SHIP TREND 419 1.37 (0.68, 2.75)
Subtotal (I-squared = 32.3%, p = 0.078) 1.03 (1.00, 1.06)
Former smokers
GOYA Males 217 0.57 (0.15, 2.15)
MIDSPAN 578 0.78 (0.43, 1.39)
ELSA 2606 0.80 (0.66, 0.96)
Inter99 1347 0.87 (0.64, 1.18)
UK Biobank 38296 0.99 (0.95, 1.04)
ALSPAC Mothers 1238 1.01 (0.80, 1.27)
NEO 2778 1.02 (0.85, 1.24)
Health2008 218 0.98 (0.48, 2.01)
SHIP 1374 1.04 (0.83, 1.32)
Whitehall II 730 1.09 (0.63, 1.89)
HUNT2 11241 1.07 (0.98, 1.18)
FINRISK 5308 1.10 (0.92, 1.31)
NSHD 1186 1.11 (0.83, 1.48)
Dan-Monica10 514 1.10 (0.70, 1.73)
Health2006 1063 1.14 (0.88, 1.49)
1958 BC 1400 1.34 (1.03, 1.75)
The 1936 Cohort 154 1.48 (0.49, 4.44)
KORA 259 1.59 (0.79, 3.23)
SHIP TREND 351 1.74 (0.79, 3.82)
Subtotal (I-squared = 16.0%, p = 0.258) 1.01 (0.98, 1.05)
Current smokers
KORA 209 0.61 (0.24, 1.55)
NSHD 565 0.79 (0.50, 1.25)
Inter99 1819 0.84 (0.65, 1.08)
Health2008 121 0.91 (0.39, 2.12)
Health2006 717 0.95 (0.65, 1.38)
FINRISK 6424 0.92 (0.75, 1.13)
SHIP TREND 214 0.93 (0.37, 2.35)
ELSA 841 0.99 (0.71, 1.38)
NEO 831 1.01 (0.67, 1.51)
ALSPAC Mothers 881 1.02 (0.79, 1.32)
UK Biobank 13344 1.04 (0.96, 1.13)
HUNT2 12617 1.10 (1.00, 1.20)
GOYA Males 394 1.11 (0.55, 2.23)
COPSAC2000 131 2.37 (0.84, 6.68)
1958 BC 1174 1.21 (0.90, 1.65)
SHIP 1225 1.24 (0.96, 1.62)
Dan-Monica10 926 1.25 (0.87, 1.80)
Whitehall II 351 1.26 (0.45, 3.47)
GOYA Females 521 1.37 (0.91, 2.04)
MIDSPAN 539 1.52 (0.85, 2.73)
The 1936 Cohort 202 2.51 (1.02, 6.15)
Subtotal (I-squared = 6.5%, p = 0.374) 1.06 (1.01, 1.11)
Ever smokers
ELSA 3447 0.83 (0.71, 0.98)
Inter99 3166 0.85 (0.70, 1.03)
GOYA Males 611 0.94 (0.51, 1.74)
Health2008 339 0.94 (0.55, 1.61)
NSHD 1751 1.01 (0.79, 1.28)
UK Biobank 51640 1.01 (0.97, 1.05)
FINRISK 11732 1.02 (0.89, 1.16)
ALSPAC Mothers 2119 1.01 (0.85, 1.20)
NEO 3609 1.03 (0.86, 1.22)
Health2006 1780 1.07 (0.86, 1.33)
MIDSPAN 1117 1.08 (0.72, 1.63)
HUNT2 23858 1.08 (1.01, 1.15)
KORA 468 1.10 (0.64, 1.92)
SHIP 2599 1.12 (0.94, 1.33)
Whitehall II 1081 1.14 (0.70, 1.85)
Dan-Monica10 1440 1.18 (0.89, 1.57)
1958 BC 2574 1.27 (1.04, 1.56)
SHIP TREND 565 1.33 (0.74, 2.41)
The 1936 Cohort 356 1.88 (0.96, 3.67)
Subtotal (I-squared = 26.9%, p = 0.135) 1.03 (1.00, 1.06)
.3
.6
1
2
3
Odds ratio per smoking-increasing allele

References

1 Power C, Elliott J. Cohort profile: 1958 British birth cohort (National Child Development Study). Int J Epidemiol 2006 Feb;**35**(1):34-41.

2 Sawcer S, Hellenthal G, Pirinen M, Spencer CC, Patsopoulos NA, Moutsianas L, et al. Genetic risk and a primary role for cell-mediated immune mechanisms in multiple sclerosis. Nature 2011 Aug 11;**476**(7359):214-9.

3 Barrett JC, Clayton DG, Concannon P, Akolkar B, Cooper JD, Erlich HA, et al. Genome-wide association study and meta-analysis find that over 40 loci affect risk of type 1 diabetes. Nat Genet 2009 Jun;**41**(6):703-7.

4 Boyd A, Golding J, Macleod J, Lawlor DA, Fraser A, Henderson J, et al. Cohort Profile: the 'children of the 90s'--the index offspring of the Avon Longitudinal Study of Parents and Children. Int J Epidemiol 2013 Feb;**42**(1):111-27.

5 Fraser A, Macdonald-Wallis C, Tilling K, Boyd A, Golding J, Davey SG, et al. Cohort Profile: the Avon Longitudinal Study of Parents and Children: ALSPAC mothers cohort. Int J Epidemiol 2013 Feb;**42**(1):97-110.

6 Freathy RM, Ring SM, Shields B, Galobardes B, Knight B, Weedon MN, et al. A common genetic variant in the 15q24 nicotinic acetylcholine receptor gene cluster (CHRNA5-CHRNA3-CHRNB4) is associated with a reduced ability of women to quit smoking in pregnancy. Hum Mol Genet 2009 Aug 1;**18**(15):2922-7.

7 Bisgaard H. The Copenhagen Prospective Study on Asthma in Childhood (COPSAC): design, rationale, and baseline data from a longitudinal birth cohort study. Ann Allergy Asthma Immunol 2004 Oct;**93**(4):381-9.

8 Rasmussen SL, Torp-Pedersen C, Borch-Johnsen K, Ibsen H. Normal values for ambulatory blood pressure and differences between casual blood pressure and ambulatory blood pressure: results from a Danish population survey. J Hypertens 1998 Oct;**16**(10):1415-24.

9 Skaaby T, Husemoen LL, Thuesen BH, Hammer-Helmich L, Linneberg A. Atopy and cause-specific mortality. Clin Exp Allergy 2014 Nov;**44**(11):1361-70.

10 Skaaby T, Husemoen LL, Roswall N, Thuesen BH, Linneberg A. Atopy and development of cancer: A population-based prospective study. JACI: In practice 2014.

11 Skaaby T, Husemoen LL, Thuesen BH, Jeppesen J, Linneberg A. The association of atopy with incidence of ischemic heart disease, stroke, and diabetes. Endocrine 2014 Jun 12.

12 Skaaby T, Husemoen LL, Thuesen BH, Fenger RV, Linneberg A. Specific IgE positivity against inhalant allergens and development of autoimmune disease. Autoimmunity 2015 Aug;**48**(5):282-8.

13 Linneberg A, Husemoen LL, Nielsen NH, Madsen F, Frolund L, Johansen N. Screening for allergic respiratory disease in the general population with the ADVIA Centaur Allergy Screen Assay. Allergy 2006 Mar;**61**(3):344-8.

14 Steptoe A, Breeze E, Banks J, Nazroo J. Cohort profile: the English longitudinal study of ageing. Int J Epidemiol 2013 Dec;**42**(6):1640-8.

15 Vartiainen E, Laatikainen T, Peltonen M, Juolevi A, Mannisto S, Sundvall J, et al. Thirty-five-year trends in cardiovascular risk factors in Finland. Int J Epidemiol 2010 Apr;**39**(2):504-18.

16 Paternoster L, Evans DM, Nohr EA, Holst C, Gaborieau V, Brennan P, et al. Genome-wide population-based association study of extremely overweight young adults--the GOYA study. PLoS One 2011;**6**(9):e24303.

17 Nohr EA, Timpson NJ, Andersen CS, Davey SG, Olsen J, Sorensen TI. Severe obesity in young women and reproductive health: the Danish National Birth Cohort. PLoS One 2009;**4**(12):e8444.

18 Thuesen BH, Cerqueira C, Aadahl M, Ebstrup JF, Toft U, Thyssen JP, et al. Cohort Profile: the Health2006 cohort, research centre for prevention and health. Int J Epidemiol 2014 Apr;**43**(2):568-75.

19 Petersen AB, Gudmann P, Milvang-Gronager P, Morkeberg R, Bogestrand S, Linneberg A, et al. Performance evaluation of a specific IgE assay developed for the ADVIA centaur immunoassay system. Clin Biochem 2004 Oct;**37**(10):882-92.

20 Aadahl M, Zacho M, Linneberg A, Thuesen BH, Jorgensen T. Comparison of the Danish step test and the watt-max test for estimation of maximal oxygen uptake: the Health2008 study. Eur J Prev Cardiol 2013 Dec;**20**(6):1088-94.

21 Byberg S, Hansen AL, Christensen DL, Vistisen D, Aadahl M, Linneberg A, et al. Sleep duration and sleep quality are associated differently with alterations of glucose homeostasis. Diabet Med 2012 Sep;**29**(9):e354-e360.

22 Osler M, Linneberg A, Glumer C, Jorgensen T. The cohorts at the Research Centre for Prevention and Health, formerly 'The Glostrup Population Studies'. Int J Epidemiol 2011 Jun;**40**(3):602-10.

23 Gabrielsen ME, Romundstad P, Langhammer A, Krokan HE, Skorpen F. Association between a 15q25 gene variant, nicotine-related habits, lung cancer and COPD among 56,307 individuals from the HUNT study in Norway. Eur J Hum Genet 2013 Nov;**21**(11):1293-9.

24 Jorgensen T, Jacobsen RK, Toft U, Aadahl M, Glumer C, Pisinger C. Effect of screening and lifestyle counselling on incidence of ischaemic heart disease in general population: Inter99 randomised trial. BMJ 2014;**348**:g3617.

25 Thuesen BH, Husemoen LL, Ovesen L, Jorgensen T, Fenger M, Gilderson G, et al. Atopy, asthma, and lung function in relation to folate and vitamin B(12) in adults. Allergy 2010 Nov;**65**(11):1446-54.

26 Bothig S. WHO MONICA Project: objectives and design. Int J Epidemiol 1989;**18**(3 Suppl 1):S29-S37.

27 Schafer T, Ruhdorfer S, Weigl L, Wessner D, Heinrich J, Wichmann HE, et al. School education and allergic sensitization in adults. Allergy 2001 Dec;**56**(12):1206-10.

28 Wichmann HE, Gieger C, Illig T. KORA-gen--resource for population genetics, controls and a broad spectrum of disease phenotypes. Gesundheitswesen 2005 Aug;**67 Suppl 1**:S26-S30.

29 Hart CL, MacKinnon PL, Watt GC, Upton MN, McConnachie A, Hole DJ, et al. The Midspan studies. Int J Epidemiol 2005 Feb;**34**(1):28-34.

30 Upton MN, McConnachie A, McSharry C, Hart CL, Smith GD, Gillis CR, et al. Intergenerational 20 year trends in the prevalence of asthma and hay fever in adults: the Midspan family study surveys of parents and offspring. BMJ 2000 Jul 8;**321**(7253):88-92.

31 Munafo MR, Timofeeva MN, Morris RW, Prieto-Merino D, Sattar N, Brennan P, et al. Association between genetic variants on chromosome 15q25 locus and objective measures of tobacco exposure. J Natl Cancer Inst 2012 May 16;**104**(10):740-8.

32 Tyrrell J, Huikari V, Christie JT, Cavadino A, Bakker R, Brion MJ, et al. Genetic variation in the 15q25 nicotinic acetylcholine receptor gene cluster (CHRNA5-CHRNA3-CHRNB4) interacts with maternal self-reported smoking status during pregnancy to influence birth weight. Hum Mol Genet 2012 Dec 15;**21**(24):5344-58.

33 Taylor AE, Morris RW, Fluharty ME, Bjorngaard JH, Asvold BO, Gabrielsen ME, et al. Stratification by smoking status reveals an association of CHRNA5-A3-B4 genotype with body mass index in never smokers. PLoS Genet 2014 Dec;**10**(12):e1004799.

34 Morris RW, Taylor AE, Fluharty ME, Bjorngaard JH, Asvold BO, Elvestad GM, et al. Heavier smoking may lead to a relative increase in waist circumference: evidence for a causal relationship from a Mendelian randomisation meta-analysis. The CARTA consortium. BMJ Open 2015;**5**(8):e008808.

35 Linneberg A, Jacobsen RK, Skaaby T, Taylor AE, Fluharty ME, Jeppesen JL, et al. Effect of Smoking on Blood Pressure and Resting Heart Rate: A Mendelian Randomization Meta-Analysis in the CARTA Consortium. Circ Cardiovasc Genet 2015 Dec;**8**(6):832-41.

36 de MR, den HM, Rabelink TJ, Smit JW, Romijn JA, Jukema JW, et al. The Netherlands Epidemiology of Obesity (NEO) study: study design and data collection. Eur J Epidemiol 2013 Jun;**28**(6):513-23.

37 Wadsworth M, Kuh D, Richards M, Hardy R. Cohort Profile: The 1946 National Birth Cohort (MRC National Survey of Health and Development). Int J Epidemiol 2006 Feb;**35**(1):49-54.

38 Kuh D, Pierce M, Adams J, Deanfield J, Ekelund U, Friberg P, et al. Cohort profile: updating the cohort profile for the MRC National Survey of Health and Development: a new clinic-based data collection for ageing research. Int J Epidemiol 2011 Feb;**40**(1):e1-e9.

39 Rousseau K, Vinall LE, Butterworth SL, Hardy RJ, Holloway J, Wadsworth ME, et al. MUC7 haplotype analysis: results from a longitudinal birth cohort support protective effect of the MUC7*5 allele on respiratory function. Ann Hum Genet 2006 Jul;**70**(Pt 4):417-27.

40 Clennell S, Kuh D, Guralnik JM, Patel KV, Mishra GD. Characterisation of smoking behaviour across the life course and its impact on decline in lung function and all-cause mortality: evidence from a British birth cohort. J Epidemiol Community Health 2008 Dec;**62**(12):1051-6.

41 Black S, Teixeira AS, Loh AX, Vinall L, Holloway JW, Hardy R, et al. Contribution of functional variation in the IL13 gene to allergy, hay fever and asthma in the NSHD longitudinal 1946 birth cohort. Allergy 2009 Aug;**64**(8):1172-8.

42 Marchini J, Howie B, Myers S, McVean G, Donnelly P. A new multipoint method for genome-wide association studies by imputation of genotypes. Nat Genet 2007 Jul;**39**(7):906-13.

43 Delaneau O, Marchini J. Integrating sequence and array data to create an improved 1000 Genomes Project haplotype reference panel. Nat Commun 2014;**5**:3934.

44 Sudlow C, Gallacher J, Allen N, Beral V, Burton P, Danesh J, et al. UK biobank: an open access resource for identifying the causes of a wide range of complex diseases of middle and old age. PLoS Med 2015 Mar;**12**(3):e1001779.

45 Wain LV, Shrine N, Miller S, Jackson VE, Ntalla I, Artigas MS, et al. Novel insights into the genetics of smoking behaviour, lung function, and chronic obstructive pulmonary disease (UK BiLEVE): a genetic association study in UK Biobank. Lancet Respir Med 2015 Oct;**3**(10):769-81.

46 Voight BF, Kang HM, Ding J, Palmer CD, Sidore C, Chines PS, et al. The metabochip, a custom genotyping array for genetic studies of metabolic, cardiovascular, and anthropometric traits. PLoS Genet 2012;**8**(8):e1002793.

47 John U, Greiner B, Hensel E, Ludemann J, Piek M, Sauer S, et al. Study of Health In Pomerania (SHIP): a health examination survey in an east German region: objectives and design. Soz Praventivmed 2001;**46**(3):186-94.

48 Volzke H, Alte D, Schmidt CO, Radke D, Lorbeer R, Friedrich N, et al. Cohort profile: the study of health in Pomerania. Int J Epidemiol 2011 Apr;**40**(2):294-307.
